# Annexing isothermal nucleotide amplification enables probe-guided amplification and direct detection at ambient temperature

**DOI:** 10.1038/s41467-026-75361-6

**Published:** 2026-07-14

**Authors:** Ariana Human-McKinnon, Madisen Pyper, MiKenzee Frazier, Emily Plant, Harrison Piper, Tyler Archibald, Anindita Roy

**Affiliations:** Seek Labs Inc., Salt Lake City, UT 84103 USA

**Keywords:** Assay systems, Medical and clinical diagnostics, Bioanalytical chemistry

## Abstract

Isothermal nucleic acid amplification offers advantages over qPCR for decentralized diagnostics but remains constrained by rigid primer design, detection complexity, and equipment dependence. Recombinase-based systems operate at lower temperatures than other isothermal methods but require long primers and auxiliary enzymatic processing for detection. Here we introduce Annexing Isothermal Nucleotide Amplification (ANINA), a probe-guided recombinase-based framework integrating amplification and detection at ambient temperatures within a single lyophilized reaction. An Annexing Probe recruits short primers through spatial annexation, enabling efficient amplification at 25 °C within 30 minutes and direct lateral flow detection without auxiliary enzymatic processing. We show this framework detects attomolar WSSV and EBV DNA in abundant host gDNA, supports target-specific detection of DNA and RNA viruses and bacteria in contrived matrices, enables quantitative real-time detection comparable to qPCR, and facilitates a fully equipment-free 45-minute sample-to-answer workflow that detects early viral infection in a natural host model with qPCR-level sensitivity and improved performance over a commercial antigen test.

## Abstract (web summary)

Decentralized diagnostics are constrained by complex detection and strict primer design in isothermal amplification methods. Here, authors introduce ANINA, a probe-guided platform that amplifies and detects pathogens at ambient temperatures in one equipment-free reaction with qPCR-level sensitivity.

## Introduction

Reliable infectious disease diagnostics are central to surveillance, clinical decision-making, and outbreak response. Delayed detection worsens outcomes and increases healthcare burden, particularly for rapidly spreading pathogens. Current diagnostic solutions face persistent trade-offs between analytical performance, speed, affordability, and accessibility, particularly in low-resource and decentralized testing settings^1–4^. Laboratory-based diagnostics, such as qPCR, offer high-sensitivity and specificity but depend on centralized infrastructure, electricity, trained personnel, and complex supply chains, while rapid antigen-based tests are deployable and inexpensive but lack sensitivity in the early stages of infection^5,6^. The COVID-19 pandemic illustrated at a global scale how the diagnostic gap between molecular accuracy and real-world accessibility affects surveillance, outbreak containment, and timely treatment initiation^7–11^. Indeed, the pandemic highlighted the necessity for instrument-free, cost-effective molecular diagnostics that combine qPCR-level sensitivity with minimal infrastructure requirements and can be rapidly configured for various disease targets^5^.

Isothermal nucleic acid amplification technologies (NAATs), such as Loop-mediated Amplification^12^, Helicase Dependent Amplification^13^, and Recombinase Polymerase Amplification (RPA)^14^, reduce hardware requirements by eliminating thermocycling; however, they have alternative constraints. These include complex primer architectures, narrow design rules, and continued reliance on elevated incubation temperatures for optimal performance^15–18^. These limitations restrict assay flexibility and reduce robustness under decentralized conditions.

Among isothermal NAATs, RPA offers one of the lowest operating temperatures of 30–42 °C using recombinase-mediated strand invasion and primer hybridization^14,19–21^. However, RPA typically requires primers 30–36 nucleotides (nt) in length, with amplification declining below ~30 nt and often failing below 21 nt^14,22^. Unlike PCR, which enforces annealing stringency through thermal cycling, RPA lacks a robust mechanism to spatially regulate primer recruitment. As a result, long primers frequently generate background amplification and reduced signal-to-noise ratios, particularly at lower temperatures (< 35 °C). Attempts to address these constraints have included engineered recombinases^23^, primers with modified nucleotides such as LNAs or SAMRS^24^, probe-based detection strategies requiring auxiliary enzymes such as nfo-RPA^14,25^ and SHERLOCK^26,27^, and Strand-Invasion Based Amplification (SIBA)^22,28–30^, which utilizes both non-probe invasion oligonucleotides and separate detection probes. These approaches improve specificity but increase assay design complexity, total time-to-result, and manufacturing costs.

**Fig. 1:**
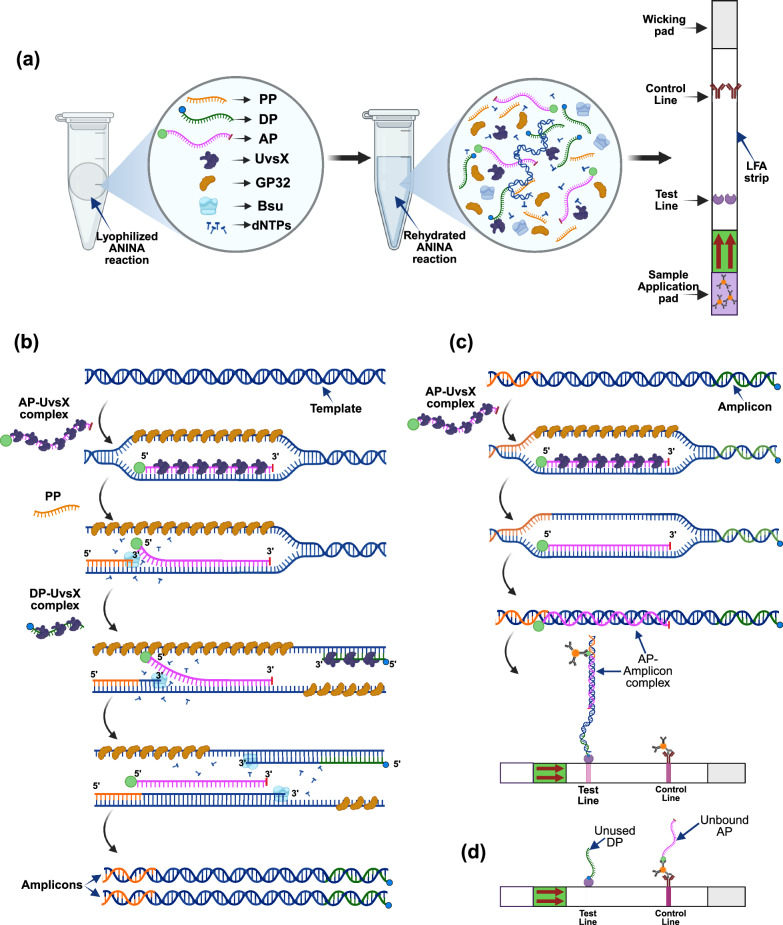
Schematic of Annexing Isothermal Nucleotide Amplification (ANINA) and direct endpoint detection. **a** Two-step process for ANINA amplification and lateral flow assay (LFA) readout. Lyophilized ANINA reactions contain Annexing Probe (AP), Probe-adjacent Primer (PP), Downstream Primer (DP), recombinase (UvsX), single-strand binding protein (GP32), strand-displacing DNA polymerase (Bsu), and other required reaction components in a shelf-stable format. The reaction is initiated by the addition of template eluted in rehydration buffer, incubated at ambient temperature for 15–30 min, and diluted directly into detection buffer for LFA readout. LFA strips contain anti-fluorescein gold nanoparticles (GNPs) in the sample pad, streptavidin at the test line, and secondary antibodies at the control line. **b** In the presence of target, the AP–recombinase complex invades the template and binds its complementary sequence. The bound AP spatially recruits the adjacent PP, enabling annexation-mediated primer loading. The biotin-labeled DP binds the opposite strand and strand-displacing polymerase extends both primers, which displaces the AP and generates biotinylated amplicons. Displaced APs participate in subsequent amplification cycles. **c** Upon depletion of free PP, fluorescein-labeled APs (FAM-AP) remain stably associated with double-stranded, biotinylated amplicons, forming detection-competent complexes. During LFA, AP-associated amplicons are captured at the test line via biotin–streptavidin interaction and produce a visible signal. **d** In the absence of target, no biotinylated amplicons are formed. Free APs migrate to the control line while primers alone bind weakly at the test line to provide dual-level specificity. Figure created in BioRender. Roy, A. (2026) https://BioRender.com/5jygk7z.

Here, we introduce Annexing Isothermal Nucleotide Amplification (ANINA), a probe-guided recombinase-based framework that integrates amplification and detection within a single lyophilized reaction (Fig. 1a) operating at a wide range of temperatures (20–45 °C) while staying between 5 and 30 min of assay time. Central to ANINA is a three-oligonucleotide architecture (Fig. 1b) consisting of an Annexing Probe (AP) with a 3′ polymerase block, a probe-adjacent primer (PP), and a downstream primer (DP). The PP and DP serve analogous functions to forward and reverse primers in other amplification systems, but their directionality is based on AP–primer spatial geometry. The AP, rather than being a passive reporter, actively participates in amplification through spatial annexation of the PP. The AP, designed to be long enough for efficient recombinase loading, interacts with recombinase (UvsX) and single-strand binding protein (GP32) to form a nucleoprotein complex that invades double-stranded DNA and binds its complementary sequence to generate a locally opened D-loop structure. We propose that this AP-mediated opening enables the adjacent PP to productively anneal, even when the PP itself is too short for efficient recombinase loading. The strand-displacing polymerase, Bsu, then extends the PP and displaces the non-extendible AP, allowing subsequent rounds of annexation. PP extension also exposes the complementary strand for efficient DP annealing and amplification progression. This spatial organization enables specific and efficient amplification under primer-length and temperature conditions poorly supported by conventional recombinase-based systems. Notably, ANINA supports efficient and specific amplification at 25 °C with PPs as short as 10 nt and DPs as short as 15 nt, lengths that are substantially below those typically supported in recombinase-based systems.

In addition to amplification control, APs remain associated with amplification products in a manner compatible with direct endpoint detection without any enzyme-mediated probe cleavage (Fig. 1c and Supplementary Fig. 1). This enables seamless integration of amplification with lateral flow detection and real-time fluorescence quantification within the same reaction framework.

In this study, we systematically characterize annexation by varying primer length, probe length, probe chemistry, and spatial positioning. We demonstrate multiplexed endpoint detection, quantitative real-time readout with ANINA (qANINA), and high analytical sensitivity and specificity across diverse nucleic acid targets and complex biological matrices. Finally, we validate ANINA in a natural host infection model using a fully instrument-free workflow, highlighting its alignment with the ASSURED framework for point-of-care diagnostics (affordable, sensitive, specific, user-friendly, rapid & robust, and deliverable)^1–4^.

## Results

### Annexing Probe enables short-primer amplification at 25 °C

**Fig. 2:**
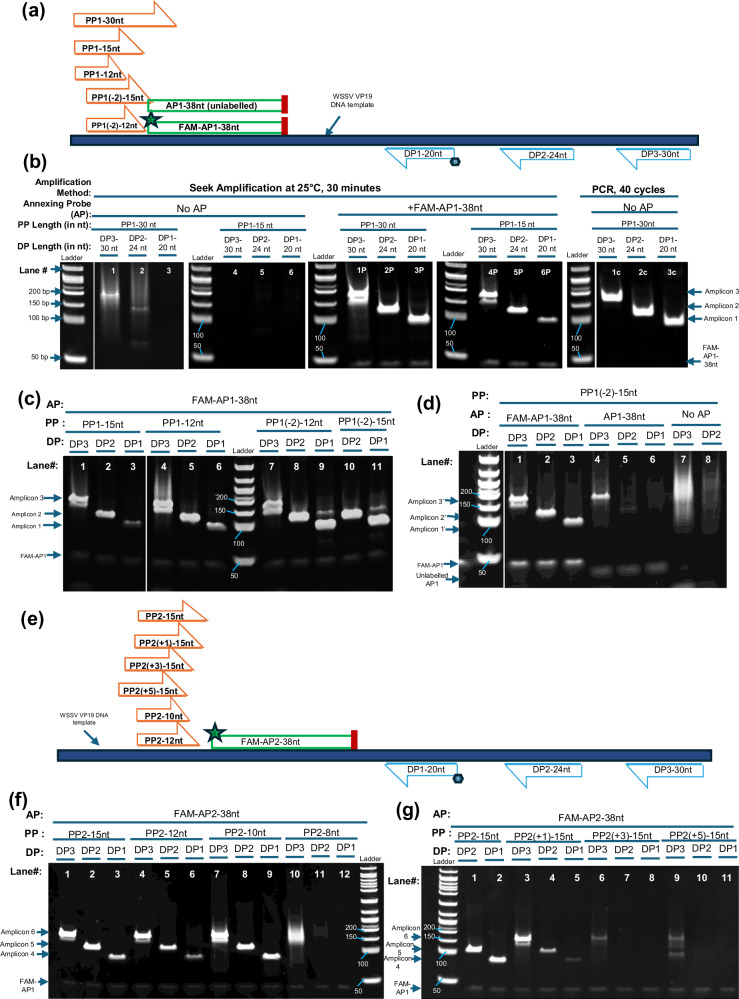
Annexing Probe enables recombinase-based amplification at 25 °C using primers far below conventional length constraints. **a**, **e** Schematics of two independent ANINA assay architectures targeting the WSSV VP19 locus, used for the corresponding PAGE analyses in (**b**–**d**) and (**f**, **g**), respectively. All reactions were performed at 25 °C for 30 min using WSSV gDNA (~10⁶ copies per reaction) extracted from infected shrimp. **a** First ANINA architecture comprising a 5′ fluorescein-labeled, 3′-blocked Annexing Probe (FAM–AP1–38 nt), probe-adjacent primer (PP1), and downstream primers (DP1–DP3), generating Amplicons 1–3 (97, 123, and 179 bp). PP variants of different lengths (30, 15, and 12 nt), a 3′-shifted PP variant (PP1(−2)−15 nt), and an unlabeled AP were also evaluated. **b** Amplification using 30 nt and 15 nt PP variants in the absence (−AP) or presence (+AP) of FAM–AP1–38 nt. Gel panels were assembled from multiple PAGE gels run under identical conditions within the same experiment. Panels 2 and 5 originate from the same gel. Marker lanes were repositioned for visual consistency, and brightness/contrast were adjusted uniformly within each gel section. **c** Amplification using shortened (15 nt, 12 nt) and 3′-shifted PP1 (PP1(−2)–15 nt) variants in the presence of FAM–AP1–38 nt. **d** Comparison of amplification using PP1(−2) in the presence of FAM-labeled AP, unlabeled AP, or no AP. Amplicons 1′–3′ are 2 bp shorter than Amplicons 1–3, respectively. **e** A second independent annexation architecture (FAM–AP2–38 nt with PP2) targeting a downstream VP19 region generating Amplicons 4–6 (81, 107, and 163 bp) with DP1–3. Progressively shorter PP2 variants (15–8 nt) and constant-length PP2 variants shifted toward the 5′ direction of the target sequence (+1, +2, +3) were also evaluated. **f** Amplification using progressively shorter PP2 variants in the presence of FAM–AP2–38 nt. **g** Amplification using constant-length, 5′-shifted PP2 variants demonstrating strong dependence of annexation efficiency on AP–PP spatial positioning independent of primer length. A 50 bp DNA ladder was used as the molecular size marker in all PAGE gels, and at least two reference sizes are indicated in each gel panel. Representative results are shown. Source data are provided as a Source Data file. All conditions were evaluated in at least two independent experimental runs with similar results. Reproducible secondary amplicon bands, most prominent with DP3, were target-dependent and absent from no-template controls.

To test whether spatially guided primer recruitment can overcome intrinsic primer-length and low-temperature constraints of recombinase-based amplification, we systematically evaluated the effect of an AP on amplification using PPs and DPs of varying length and position targeting the VP19 locus of White Spot Syndrome Virus (WSSV), *a* ~ 300 kb viral dsDNA genome (Fig. 2a). All reactions were performed at 25 °C for 30 min using gDNA from infected shrimp. Primer functionality was independently confirmed by PCR (Fig. 2b, right panel).

In the absence of an AP, amplification was restricted to longer primers that yielded weak or inconsistent products, while shorter primer combinations failed to amplify (Fig. 2b). In contrast, inclusion of an AP enabled robust and specific amplification across all tested primer-length combinations, including PPs as short as 15 nt paired with short DPs (20 nt). Amplification occurred despite the absence of probe guidance at the DP-binding site, indicating annexation of the PP is sufficient to initiate productive amplification.

To separate primer-length effects from spatial positioning, we evaluated shorter PP1 variants (15 and 12 nt) and a 3′-shifted variant, PP1(−2), which preserves primer length but alters AP–PP spacing. In the presence of AP, both 15 nt and 12 nt PPs supported efficient amplification with all DPs (Fig. 2c). The shifted PP1(−2) improved yield with the shortest DP, which is potentially because it has a substantially lower predicted secondary structure melting temperature than the PP1–15 nt primer (3.5 °C versus 19.7 °C; Table 1), indicating reduced intramolecular folding at 25 °C and greater primer accessibility for annealing and extension.

**Table 1:** WSSV VP19 ANINA oligonucleotide sequences and corresponding modification chemistries

Oligonucleotide Name	Sequence (with labels or modifications)	GC %	Tm (°C)	ΔG (kcal/mol)	2° Tm (°C)
**Annexing Probe (AP) sequences**					
AP1–38 nt	CTCTTTTTGATCGTTGCTATCTCAATTGGTATCCTCGT/3InvdT/	38.5	72.8	−0.4	28.1
FAM-AP1–38 nt	/56-FAM /CTCTTTTTGATCGTTGCTATCTCAATTGGTATCCTCGT/3InvdT/	38.5	72.8	−0.4	28.1
Btn-AP1–38 nt	/5Biosg /CTCTTTTTGATCGTTGCTATCTCAATTGGTATCCTCGT/3InvdT/	38.5	72.8	−0.4	28.1
FAM-AP1–34 nt	/56-FAM/CTCTTTTTGATCGTTGCTATCTCAATTGGTATCC/3InvdT/	37.1	70.9	−0.4	28.1
FAM-AP1–29 nt	/56-FAM/CTCTTTTTGATCGTTGCTATCTCAATTGG/3InvdT/	36.7	69.1	−0.4	29.9
FAM-AP2–38 nt	/56-FAM /CTATCTCAATTGGTATCCTCGTCCTGGCCGTCATGAAT/3InvdT/	46.2	74.8	−0.4	29.5
ROX-AP2–38 nt	/56-ROXN /CTATCTCAATTGGTATCCTCGTCCTGGCCGTCATGAAT/3InvdT/	46.2	74.8	−0.4	29.5
Cy5-AP2–38 nt	/5Cy5 /CTATCTCAATTGGTATCCTCGTCCTGGCCGTCATGAAT/3InvdT/	46.2	74.8	−0.4	29.5
**Probe-adjacent Primer (PP) sequences**					
PP1–15 nt	TGGCTCGCATGGGTC	66.7	65.5	0.3	19.7
PP1–10 nt	TGGCTCGCAT	60.0	51.2	0.3	19.7
PP1–12 nt	TGGCTCGCATGG	66.7	58.6	0.3	19.7
PP1–14 nt	TGGCTCGCATGGGT	64.3	64.9	0.3	19.7
PP1–20 nt	TGGCTCGCATGGGTCTCTTT	55.0	69.8	0.3	19.7
PP1–25 nt	TGGCTCGCATGGGTCTCTTTTTGAT	48.0	71.7	0.3	19.7
PP1–30 nt	TGGCTCGCATGGGTCTCTTTTTGATCGTTG	50.0	74.0	0.3	19.7
PP1–35 nt	TGGCTCGCATGGGTCTCTTTTTGATCGTTGCTATC	48.6	75.2	−1.7	36.6
PP1(−2)–15 nt	GCTCGCATGGGTCTC	66.7	63.0	0.9	3.5
PP1(−2)–12 nt	GCTCGCATGGGT	66.7	58.6	0.9	3.5
PP1(−4)–15 nt	TCGCATGGGTCTCTT	53.3	61.3	0.9	7.5
PP1(−6)–15 nt	GCATGGGTCTCTTTT	46.7	57.5	0.9	7.5
PP1(−9)–15 nt	TGGGTCTCTTTTTGA	40.0	55.9	0.5	12.4
PP1(−11)–15 nt	GGTCTCTTTTTGATC	40.0	52.2	0.5	12.4
PP2–15 nt	CTTTTTGATCGTTGC	40.0	54.7	2.4	<0
PP2–12 nt	CTTTTTGATCGT	33.3	45.1	2.5	<0
PP2–10 nt	CTTTTTGATC	30.0	33.1	2.5	<0
PP2–8 nt	CTTTTTGA	25.0	21.8	2.5	<0
PP2(+1)–15 nt	TCTTTTTGATCGTTG	33.3	52.9	0.5	12.4
PP2(+3)–15 nt	TCTCTTTTTGATCGT	33.3	53.6	0.5	12.4
PP2(+5)–15 nt	GGTCTCTTTTTGATC	40.0	52.2	0.5	12.4
PP2(−2)–15 nt	TTTTGATCGTTGCTA	33.3	54.5	2.4	<0
PP2(−4)–15 nt	TTGATCGTTGCTATC	40.0	54.4	0.3	21.4
PP2(−6)–15 nt	GATCGTTGCTATCTC	46.7	54.2	0.4	20.5
PP2(−8)–15 nt	CGTTGCTATCTCAAT	40.0	54.4	0.3	20.1
PP2(−10)–15 nt	TTGCTATCTCAATTG	33.3	51.7	0.4	19.2
PP2–19 nt	CTTTTTGATCGTTGCTATC	36.8	58.9	0.3	21.4
PP2–24 nt	CTTTTTGATCGTTGCTATCTCAAT	33.3	64.1	−0.4	29.9
PP2–35 nt	CTTTTTGATCGTTGCTATCTCAATTGGTATCCTCG	40.0	71.1	−0.4	28.1
PP2–15 nt-G (1 nt 3′ mismatch)	CTTTTTGATCGTTGG	40.0	53.8	2.2	<0
PP2-15nt-A (1 nt 3′ mismatch)	CTTTTTGATCGTTGA	33.3	52.9	1.5	<0
PP2–15 nt-T (1 nt 3′ mismatch)	CTTTTTGATCGTTGT	33.3	53.3	2.4	<0
PP2–15 nt-CG (2 nt 3′ mismatch)	CTTTTTGATCGTTCG	40.0	54.0	0.6	16.2
PP2–15 nt-AT (2 nt 3′ mismatch)	CTTTTTGATCGTTAT	26.7	49.7	1.3	<0
PP2–15 nt-TA (2 nt 3′ mismatch)	CTTTTTGATCGTTTA	26.7	50.0	2.3	<0
**Downstream Primer (DP) sequences**					
DP1–20 nt	TCGCTGTCCTTCTTTGGTCC	55.0	67.6	−0.4	30.1
DP2–24 nt	CATCGGTGTCCTTATCAGTGTCAG	50.0	67.4	−0.4	28.9
DP3–30 nt	GGTCCTGTTCTTATATTTGTCCTCATCATC	40.0	67.9	0.6	15.6
Btn-DP1–20 nt	/5Biosg/TCGCTGTCCTTCTTTGGTCC	55.0	67.6	−0.4	30.1
Btn-DP2–24 nt	/5Biosg/CATCGGTGTCCTTATCAGTGTCAG	50.0	67.4	−0.4	28.9
FAM-DP2–24 nt	/56-FAM/CATCGGTGTCCTTATCAGTGTCAG	50.0	67.4	−0.4	28.9
DIG-DP2–24 nt	/5DigN/CATCGGTGTCCTTATCAGTGTCAG	50.0	67.4	−0.4	28.9
DP2–35 nt	CATCGGTGTCCTTATCAGTGTCAGAATCGCTGTCC	51.4	74.2	−2.4	44.3
DP2–30 nt	CATCGGTGTCCTTATCAGTGTCAGAATCGC	50.0	71.7	−0.4	28.9
DP2–20 nt	CATCGGTGTCCTTATCAGTG	50.0	63.5	0.1	23.4
DP2–18 nt	CATCGGTGTCCTTATCAG	50.0	60.5	0.3	20.5
DP2–15 nt	CATCGGTGTCCTTAT	46.7	55.9	0.3	20.5

To confirm AP dependence, reactions with PP1(−2) were repeated without AP or with an unlabeled AP. Reactions lacking AP produced nonspecific products, whereas the unlabeled AP partially restored specificity but at reduced yield compared to the 5′-FAM-labeled AP (Fig. 2d), indicating probe label contributes towards annexation efficiency.

To test sequence and architectural generality, we designed a second independent annexation set (AP2/PP2) targeting a distinct region of VP19 (Fig. 2e). Using this architecture, PP2 variants as short as 10 nt supported efficient amplification with all DPs, whereas an 8 nt primer failed (Fig. 2f). These results establish a lower functional primer-length limit for recombinase-based amplification well below previously reported thresholds.

Finally, to isolate spatial effects independent of primer length, we evaluated constant-length (15 nt) PP variants shifted progressively relative to the AP (Fig. 2e and Supplementary Fig. 2a). Increasing 5′ distance from the AP beyond 17 nt sharply reduced amplification efficiency with short DPs (Fig. 2g). In contrast, shortening primers while maintaining AP proximity preserved amplification (Fig. 2f). Progressive 3′ shifts retained amplification until AP–PP separation was reduced to 8 nt, beyond which efficiency declined (Supplementary Fig. 2b, c).

Together, these results indicate that annexation efficiency is governed primarily by the spatial distance between the 5′ ends of the AP and PP, rather than primer length alone. ANINA therefore enables efficient amplification from large genomic templates at 25 °C using primers as short as 10 nt, conditions not robustly accessible using standard recombinase-based methods.

### Annexing Probe enables direct attomolar endpoint detection

Probe-based detection strategies typically require hybridization to single-stranded amplicons or additional enzymatic or thermal steps to detect double-stranded amplicons^15,31,32^. Recombinase-mediated strand invasion away from free DNA ends is predicted to be topologically transient^33–35^, limiting stable association of recombinase-loaded probes with double-stranded amplicons. We therefore tested whether the AP could support direct endpoint detection using universal lateral flow assay (LFA) strips that capture tagged amplicons via anti-FAM gold nanoparticles and requires stable AP–amplicon association (Fig. 1c, d).

**Fig. 3:**
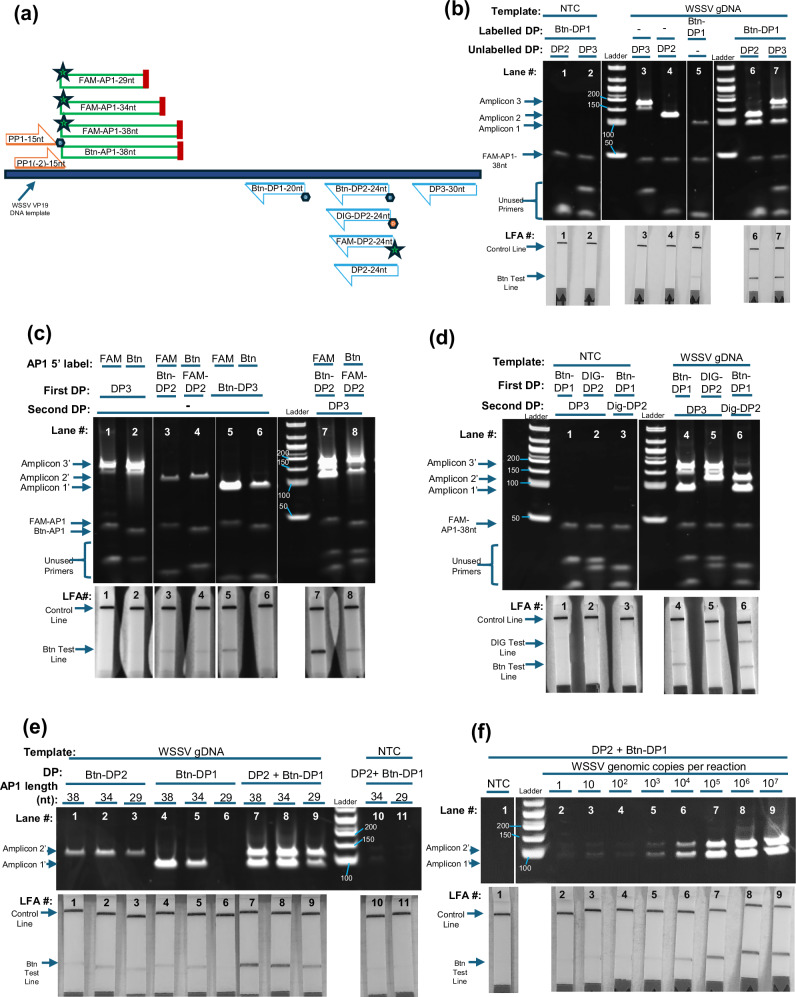
Annexing Probe enables direct endpoint detection of ANINA amplicons without auxiliary enzymatic processing. **a** Schematic of the ANINA architecture targeting the WSSV VP19 locus, showing positions of the 5′ FAM-labeled AP1–38 nt, PP1–15 nt, the 3′-shifted variant PP1(−2)–15 nt, and DPs (DP1–DP3). PP1–15 nt generates Amplicons 1–3 (97, 123, and 179 bp) with DP1–DP3, whereas PP1(−2)–15 nt generates Amplicons 1′–3′, which are 2 bp shorter than the corresponding Amplicons 1–3. Variants of AP length and labeling as well as labeled DPs were used as indicated. The 5′ labels evaluated were fluorescein (FAM), biotin (Btn), and digoxigenin (DIG). **b**–**f** Representative PAGE (top) and corresponding lateral flow assay (LFA; bottom) readouts of ANINA reactions performed at 25 °C for 30 min using gDNA from WSSV-infected shrimp or no-template controls (NTC). Panel designations indicate (**b**) single-plex detection using a 1 T LFA strip for detection of Btn-labeled amplicons associated with FAM-AP; **c** label swapping between AP and DP, with FAM and Btn exchanged between oligonucleotides; **d** duplexed detection of Btn- and DIG-labeled amplicons associated with FAM-AP using 2 T LFA strips containing separate Btn and DIG test lines; **e** probe-length dependence on amplification and detection; and **f** analytical sensitivity of WSSV detection (1–10^7^ copies per reaction). A 50 bp DNA ladder was used as the molecular size marker in all PAGE gels and at least two reference sizes are indicated in each gel panel. Representative results are shown. Source data are provided as a Source Data file. All amplification conditions were evaluated in at least two independent experimental runs with similar results.

We evaluated LFA detection of WSSV VP19 amplicons (Fig. 3a) following organic extraction to remove reaction proteins. Reactions containing a biotin-labeled DP produced clear LFA test lines only in the presence of template, whereas reactions lacking labeled primers showed no LFA signal despite robust gel amplification (Fig. 3b). Inclusion of an additional DP in semi-nested configuration increased LFA signal intensity, consistent with increased amplicon yield (Supplementary Fig. 1)^36^. These results indicate AP associates with amplification products in a detection-competent form that persists after protein removal.

We next examined the influence of probe labeling. Swapping fluorophore and affinity tags between AP and DP preserved detection logic but decreased yield in a primer-length–dependent manner (Fig. 3b). Exchanging 5′ FAM label of AP with ROX- and Cy5-labels supported amplification at 30 °C but significantly reduced specificity and yield at 25 °C (Supplementary Fig. 3a). Across conditions, FAM-labeled APs produced the most consistent amplification and detection. These findings indicate that probe label chemistry contributes directly to annexation efficiency.

Fluorescence imaging of gels confirmed FAM-labeled DPs generated fluorescent amplicons, whereas unlabeled DPs did not (Supplementary Fig. 3b). Under standard PAGE conditions, the AP migrated separately from the amplification products, and no fluorescent signal co-migrated with unlabeled amplicons, confirming the AP is neither extended nor incorporated into the product.

Because AP–amplicon associations persisted after organic extraction and enabled LFA detection, we analyzed amplicons under non-standard PAGE conditions designed to preserve higher-order structures^37–42^. Under these conditions, additional slower-migrating, fluorescence-positive bands were observed that were not prominent under standard UV visualization (Supplementary Fig. 3c). These observations are consistent with the formation of AP-associated higher-order structures that are stabilized under detection conditions but partially disrupted during conventional electrophoresis.

We next tested whether multiple labeled amplicons could be detected within a single reaction. Using digoxigenin- and biotin-labeled DPs in combination, we observed independent detection at two LFA test lines without cross-interference in template-positive reactions (Fig. 3d), demonstrating multiplex-compatible endpoint detection. We also examined the effect of AP length. APs of 38 and 34 nt supported robust amplification and LFA detection, whereas a 29 nt probe required inclusion of an additional DP to support efficient amplification with the shortest DP (Fig. 3e), consistent with known recombinase–oligonucleotide length dependencies. Finally, analytical sensitivity was assessed using serial dilutions of WSSV gDNA. Both PAGE and LFA detection were reliable at 1–10 copies per reaction (Fig. 3f), corresponding to attomolar sensitivity at 25 °C.

Together, these results demonstrate the APs form stable, detection-competent complexes with ANINA amplicons, enabling multiplexed, instrument-free endpoint detection with single-copy sensitivity and no auxiliary enzymatic processing.

### Annexation expands primer design space at ambient temperature

**Fig. 4:**
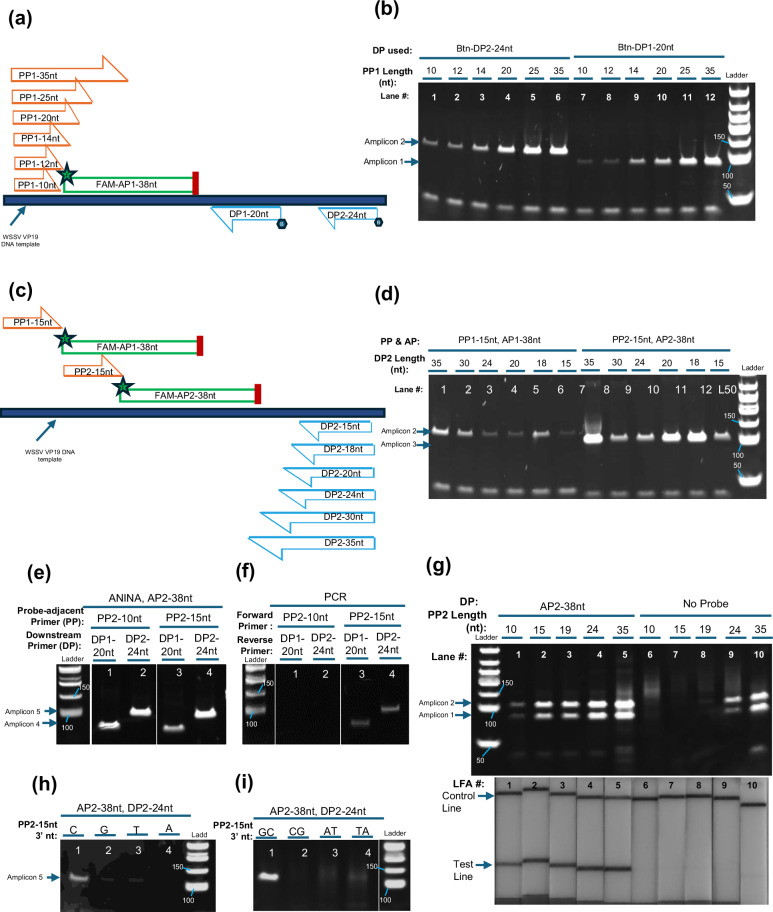
Annexation relaxes primer length and thermodynamic constraints in recombinase-based amplification. **a**, **c** Schematics of ANINA assay architectures used for the corresponding PAGE analysis of amplification reactions shown in (**b**, **d**), targeting the WSSV VP19 locus. ANINA reactions were performed at 25 °C for 30 min using WSSV gDNA extracted from infected shrimp. **a**, **b** Effect of probe-adjacent primer (PP) length on amplification in the presence of an Annexing Probe (AP1–38 nt). PP1 variants (10, 12, 14, 20, 25, and 35 nt) were evaluated with DP1–20 and DP2–24 nt, generating constant amplicon sizes for each DP architecture. **c**, **d** Effect of downstream primer (DP) length using two independent AP/PP architectures (AP1/PP1 and AP2/PP2). DP2 variants (35, 30, 24, 18, 15 nt) were tested while maintaining constant amplicon size. **e**, **f** Comparison of ANINA (**e**) and PCR (**f**) amplification using short, low GC%, low-melting-temperature PP (PP2–15 and PP2–10 nt). PCR reactions were performed using matched primers under standard cycling conditions. The gel image shown in (**e**) is reproduced from Fig. 2e. **g** Effect of PP length on amplification using two DPs (semi-nested design) in the presence or absence of AP2–38 nt. PP2 variants (10, 15, 19, 24, 35 nt) were evaluated with Btn-DP1–20 and DP2–24 nt. Top and bottom panels show PAGE analysis and corresponding LFA readout. **h**, **i** Effect of single or double 3′-terminal mismatches in PP2-15nt on amplification specificity. Lane 1 on both gel images used PP2-15nt with no mismatch to template. Lanes 2–4 contain primers carrying one or two 3′-terminal mismatches as indicated. A 50 bp DNA ladder was used as the molecular size marker in all PAGE gels and at least two reference sizes are indicated in each gel panel. Representative results are shown. Source data are provided as a Source Data file. All amplification conditions were evaluated in at least two independent experimental runs with similar results.

We next examined whether annexation broadly relaxes primer length and thermodynamic constraints that limit conventional recombinase-based amplification. Using the WSSV VP19 locus, we systematically varied PP and DP lengths while maintaining constant amplicon size (Fig. 4a–d). All reactions were performed at 25 °C using WSSV gDNA.

In the presence of an AP, efficient amplification was observed across a wide PP length range (10–35 nt) (Fig. 4a, b). Although amplification yield increased with PP length, specificity was preserved even for 10–14 nt primers. Endpoint LFA detection mirrored gel results, with reduced signal only at the shortest PP–DP combinations (Supplementary Fig. 4a). Using a commercial RPA formulation, we show detectable amplification with the short DP was restricted to longer PPs (≥ 30 nt), highlighting the intrinsic length constraints of conventional recombinase-based amplification at ambient temperatures are not unique to the formulation used in this study (Supplementary Fig. 4b).

To test whether ANINA also expands flexibility of DP length, DPs ranging from 15 to 35 nt (Fig. 4c) were evaluated while maintaining constant amplicon size using two independent AP–PP pairs (AP1/PP1 and AP2/PP2). Across both architectures, the AP supported specific amplification, including reactions where both primers were only 15 nt long (Fig. 4d). These results demonstrate annexation expands primer-length flexibility for both probe-adjacent and DPs and is not restricted to a specific sequence context.

Annexation also relaxes thermodynamic constraints. Efficient amplification was observed across primers spanning broad GC content and predicted melting temperatures (Tm) (Table 1 and Fig. 2a–d). To further challenge this paradigm, we paired a low-Tm 10 nt PP (30% GC; predicted Tm ~30 °C) with high-Tm DPs (~ 67 °C) (Fig. 4e, Table 1). These primers failed to amplify by PCR under standard cycling conditions while ANINA reactions produced clear and specific amplification at 25 °C using the same primer combinations (Fig. 4e, f).

We further evaluated PP length variants (10–35 nt) in semi-nested configurations using two DPs. In the presence of an AP, all PP lengths produced specific products detectable by both PAGE and LFA, whereas in its absence only longer primers amplified efficiently (Fig. 4g). Time- and temperature-dependence experiments showed amplification was detectable after 15 min at 25°C and remained robust across an operating temperature range of 20–40 °C in this experiment and under bench-top ambient conditions (~ 20–22 °C), with signal increasing with incubation time and temperature (Supplementary Fig. 5).

Finally, sequence specificity was assessed by introducing single- or double-nucleotide mismatches at the 3′ terminus of PP2–15 nt. Single mismatches substantially reduced amplification and double mismatches abolished detectable product formation (Fig. 4h, i), indicating annexation preserves stringent sequence discrimination at the primer extension site.

Together, these results show ANINA’s amplification performance is governed primarily by AP-guided primer recruitment, allowing both PPs and DPs to span design spaces that are not robustly supported by conventional PCR or isothermal assays.

### ANINA detects diverse pathogens in complex human matrices

**Fig. 5:**
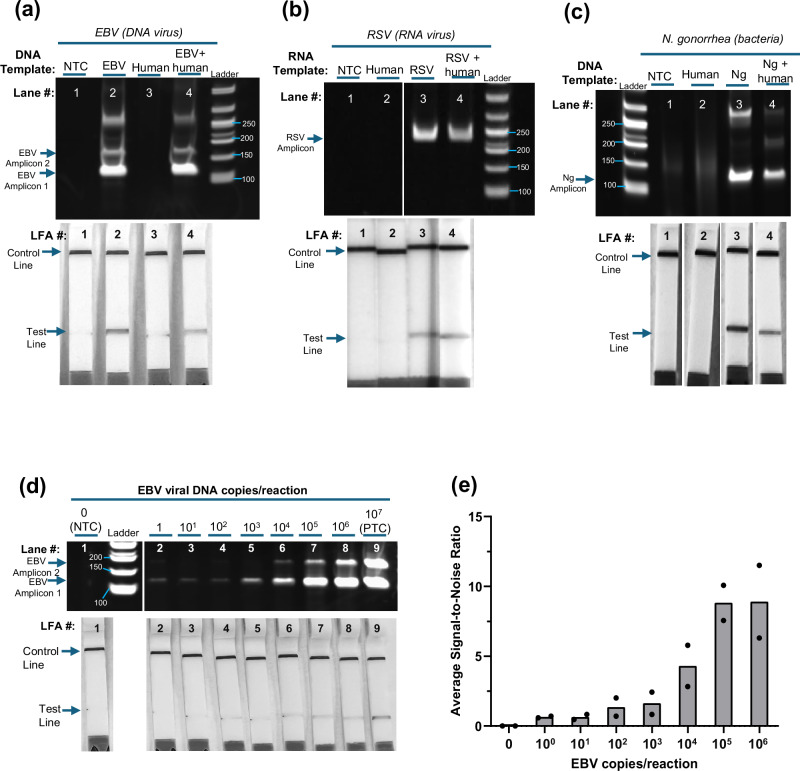
ANINA enables specific detection of DNA and RNA pathogens from complex human sample matrices. ANINA reactions were performed at 25 °C for 30 min using nucleic acids extracted from contrived human sample matrices spiked with the target pathogen. **a**–**c** Representative PAGE (top) and corresponding lateral flow assay (LFA; bottom) readouts of ANINA reactions targeting (i) Epstein–Barr virus (EBV), (ii) respiratory syncytial virus (RSV), and (iii) *Neisseria gonorrhoeae* (Ng). Human saliva was used as the background matrix for EBV and RSV and human urine for Ng. Each panel includes no-template controls (NTC), human nucleic acid–only controls, pathogen-only templates, and combined pathogen + human templates. The PAGE and LFA images shown in (**a**) and (**b**) are representative of at least three independent experiments with similar results. All amplification conditions shown in the representative images (**c**) were evaluated in at least two independent experimental runs with similar results. **d** Representative PAGE (top) and corresponding LFA (bottom) readouts showing analytical sensitivity of EBV detection (1–10^7^ copies per reaction) in the presence of human background DNA across two independent amplification experiments. **e** Quantification of LFA signal-to-noise ratios from (**d**), calculated by normalizing test-line intensity to the corresponding negative control. Bars represent the mean of two independent amplification experiments (*n* = 2), and dots indicate individual measurements. A 50 bp DNA ladder was used as the molecular size marker in all PAGE gels and at least two reference sizes are indicated in each gel panel. Source data are provided as a Source Data file.

To evaluate generalizability of the ANINA framework beyond WSSV, we applied ANINA to clinically relevant human pathogens spanning distinct nucleic acid classes: Epstein–Barr virus (EBV; dsDNA), respiratory syncytial virus (RSV; ssRNA), and *Neisseria gonorrhoeae* (bacterial DNA) (Fig. 5a–c). Target-specific APs, PPs, and DPs were designed for each organism (Table 2). EBV used two DPs (EBV Btn-DP1–24 nt, EBV DP2–29 nt).

**Table 2:** ANINA and qPCR oligonucleotide sequences and corresponding modification chemistries for EBV, *N. gonorrhoeae*, RSV, and WSSV (only qPCR) targets

Genomic Target	Oligo Name	Sequence (with labels or modifications)	GC %	Tm (°C)	ΔG (kcal/mol)	2° Tm (°C)
**ANINA: Annexing Probe (AP) sequences**						
EBV *BHRF1* (DNA, virus)	EBV AP-40 nt	/56-FAM /ACTTTGAGTCTGTTAGTTATATGTAGTTATTTATTTATCT/3invdT/	22.0	68.3	0.4	18.3
*N. gonorrhoeae* (Ng) TIGR00645 (DNA, bacteria)	Ng AP-33 nt	/56-FAM /TAAGTTTTTGAAATCGTTGAAGCATCTGGTCAT/3invdT/	33.3	70.8	−0.3	29.4
RSV A-Long strain, *M* gene (ssRNA, virus)	RSV AP-34 nt	/56-FAM/ TACATAAAGCCGCAAAGTCAATTCATAGTAGATC/3invdT/	35.3	69.8	1.0	8.2
**ANINA: Probe-adjacent Primer (PP) sequences**						
EBV *BHRF1* (DNA)	EBV PP-12 nt	CTTGCTGGACTG	58.3	51.6	1.8	<0
*N. gonorrhoeae* TIGR00645 (DNA)	Ng PP-25 nt	CGATTTGTGCCTATAAGTTTTTGAA	32.0	64.7	0.3	19.8
RSV A *M* gene (ssRNA)	RSV PP-15 nt	AAGGAGCATTCAAAT	33.3	54.3	−0.3	29.9
**ANINA: Downstream Primer (DP) sequences**						
EBV *BHRF1* (DNA)	EBV Btn-DP1–24 nt	/5Biosg/GACCCTGATTCATATAAAGTGCTG	41.7	64.7	0.2	20.4
	EBV DP2–29 nt	CTGTGTCACCCTTGCCTAATATCCTACTG	48.3	70.0	0.5	14.1
*N. gonorrhoeae* TIGR00645 (DNA)	Ng Btn-DP-20 nt	/5Biosg/GCAATCATAACCACATCAAT	35.0	61.0	2.1	<0
RSV A *M* gene (ssRNA)	RSV Btn-DP-25 nt	/5Biosg/GGTGAAGTGAAGAATGTAGGTAGAA	40.0	65.6	2.2	<0
**qPCR: Forward Primer (FP), Reverse Primer (RP), and Probe sequences**						
WSSV *VP28* (DNA)	FP	GACCAAGACCATCGAAACCC	55.0	62.5	1.3	<0
	RP	ACACATCAGTCATCTTGAAG	40.0	57.6	0.1	23.1
	Probe	/56-FAM /CCTCAGCAGTCACAGGAATGCGGAG/3BHQ_1/	60.0	69.7	−5.6	58.4
Shrimp *GAPDH* (DNA)	FP	ACCGCCAACATGTCGAAGAT	50.0	64.2	−0.5	32.1
	RP	GGGTTTCAGACACTTACCTCGG	54.5	64.1	−0.8	34.3
	Probe	/5HEX/CATCGGTCGCCTGGTGCTCCG/3BHQ_1/	71.4	70.6	−3.3	50.0
EBV *BHRF1* (DNA)	FP	CTTGCTGGACTGACTTTGAG	50.0	60.5	0.8	10.9
	RP	GACCCTGATTCATATAAAGTGCTG	41.7	61.3	0.5	15.9

For EBV and RSV, human saliva served as the background matrix; for *N. gonorrhoeae*, simulated urine was used. Pathogen-positive samples were generated by spiking viral or bacterial material into the respective matrices, followed by nucleic acid extraction alongside matched blank-matrix controls. RSV amplification incorporated reverse transcriptase (RT) for one-pot isothermal reverse transcription and recombinase-based amplification at 25 °C. Across all targets, robust and specific amplification was observed only in pathogen-containing samples, with no detectable products or lateral flow signal in no-template or human-only controls (Fig. 5a–c).

To assess analytical sensitivity under human matrix conditions, EBV-specific oligonucleotides (EBV PP-12 nt, EBV Btn-DP1–24 nt, EBV DP2–29 nt, EBV FAM-AP-40 nt) were lyophilized with reaction components to form ready-to-use beads. Processed saliva samples spiked with EBV across a dilution series (10⁷ copies to single-copy input per 50 µL reaction) were used to initiate amplification. Both PAGE and lateral flow analysis revealed reliable detection at 1–10 copies per reaction (Fig. 5d, e), corresponding to attomolar sensitivity at 25 °C.

Together, these findings demonstrate that ANINA supports sensitive and specific detection of DNA and RNA pathogens in contrived human matrices using a unified, ambient-temperature reaction architecture. Easy adaptation of the ANINA framework across a wide range of target types also underscores its generalizability and platform capability.

### Real-time fluorescence detection using qANINA

Given the specificity and rapid kinetics of ANINA, we next evaluated whether incorporation of a DNA-intercalating dye (EvaGreen) would enable real-time fluorescence monitoring (qANINA) without compromising amplification performance. Reactions using ANINA sets targeting EBV (EBV PP-12 nt, EBV DP1–24 nt, and EBV AP-40 nt; 107 bp amplicon) were run isothermally at 39 °C on a qPCR instrument without thermocycling, with fluorescence recorded every 10 s.

**Fig. 6:**
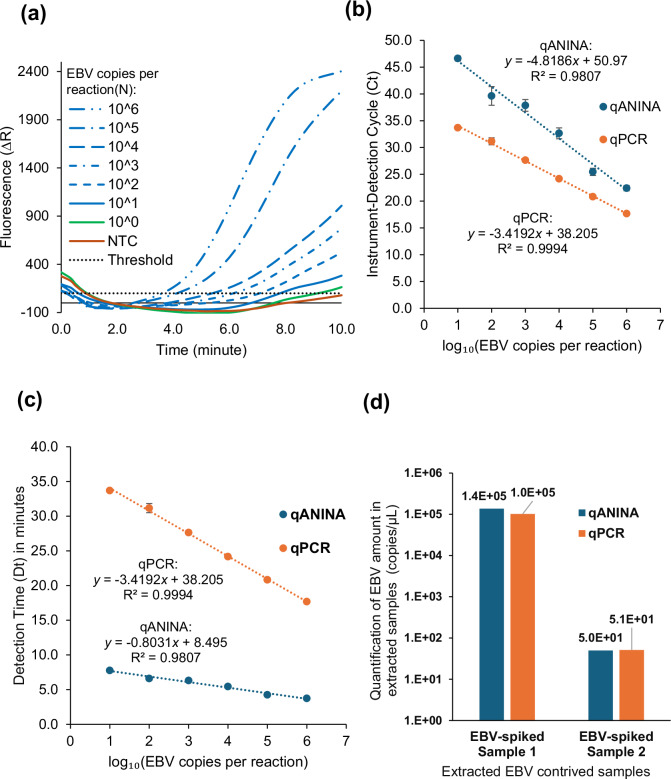
Quantitative real-time detection of EBV using qANINA compared with qPCR. qANINA reactions were performed by adding EvaGreen dye to EBV-specific ANINA reactions (107 bp target amplicon) and monitoring fluorescence isothermally at 39 °C on an Agilent AriaMx instrument, with signal acquisition every 10 s for 10 min. For comparison, dye-based qPCR was performed on the same instrument using identical EBV standards and a rapid-cycling SYBR Green qPCR master mix. **a** Real-time qANINA amplification curves for EBV genomic standards (10^0^–10⁶ copies per reaction) and no-template controls (NTC), plotted as normalized fluorescence (ΔR) versus time. Plot is representative of four independent amplification reactions performed for each input concentration. A fixed fluorescence threshold (ΔR = 100) was selected such that NTCs remained below threshold throughout the run. **b** Instrument-reported cycle threshold (Ct) versus log₁₀ EBV copy number for qANINA and qPCR. **c** Detection time (Dt) versus log₁₀ EBV copy number, where Dt is defined as the elapsed time required to cross the fixed fluorescence threshold. Points and error bars in (**b**, **c**) represent mean ± standard deviation from *n* = 4 separately assembled technical amplification reactions per EBV input concentration, prepared from the same EBV gDNA dilution. One data point for the 10-copy EBV standard was excluded from qANINA analysis as an outlier. Two data points for the 10-copy EBV standard were excluded from qPCR analysis because no amplification was detected. To keep standard-curve slope estimates comparable across methods, the single-copy (10^0^) EBV standard was excluded from slope calculation because it was only detected by qANINA but not by qPCR. Plots represent least-squares linear regression fits of Ct (**b**) or Dt (**c**) as a function of log₁₀(EBV copies per reaction). Regression equations and *R*² values are shown; *R*² indicates goodness of fit. Effective doubling time was calculated from the slope of the Dt versus log₁₀(EBV copies per reaction) regression as described in the “Methods”. qANINA and qPCR experiments were independently repeated two times with similar results. Quantification of EBV-spiked saliva samples using qANINA and qPCR standard curves. Values represent the mean of two technical amplification replicates per sample (*n* = 2). Source data are provided as a Source Data file.

qANINA produced robust, concentration-dependent amplification curves across six orders of magnitude of EBV gDNA input (10^0^–10⁶ copies per reaction), while no-template controls (NTCs) remained below threshold throughout the 10-min acquisition window (Fig. 6a). Using a fixed fluorescence threshold, instrument-reported cycle threshold (Ct) values exhibited a strong log-linear relationship with input copy number over the fitted shared range used for qANINA and qPCR comparison (*R*² = 0.98; *n* = 4) (Fig. 6b).

Because fluorescence sampling occurred at fixed 10-second intervals, Ct values correspond directly to elapsed time. Detection time (Dt), defined as the time required for average fluorescence to cross the fixed threshold^17^, scaled linearly with log₁₀ of EBV input (*R*² = 0.98), with signal detected within ~3–8 min across the tested range (Fig. 6c). For comparison, a dye-based rapid qPCR performed on the same instrument using identical standards (*R*² = 0.999, *n* = 4) required ~17–34 min to reach threshold across the same input range. Additionally, only qANINA was able to detect the single-copy EBV standard (all replicates) at ~9.3 min, demonstrating single-copy analytical sensitivity. Quantification of EBV-spiked saliva samples using qANINA closely matched qPCR-derived values, demonstrating accurate quantification in a complex biological matrix (Fig. 6d).

From the slope of the Dt relationship, the effective doubling time for EBV qANINA was ~14.5 s while that of the rapid qPCR assay was ~1 min (consistent with manufacturer claims). Similar detection-time scaling (~ 15.25 s doubling time) was observed for WSSV gDNA using independent ANINA probe-primer sets (Supplementary Fig. 6), indicating the observed kinetics reflect the annexation-based amplification mechanism rather than target-specific effects.

Thus, qANINA achieved detection approximately four-fold faster than the rapid qPCR assay at equivalent template concentrations. Together, these results demonstrate that qANINA enables rapid, quantitative detection with qPCR-like log-linearity under isothermal conditions (39 °C). By removing reliance on thermocycling, qANINA simplifies instrument needs for real-time and quantitative molecular assays and expands the application of ANINA to laboratories or point-of-care scenarios with access to electricity or battery-powered fluorimeters.

### Instrument-free ANINA detection in a natural host model

**Fig. 7:**
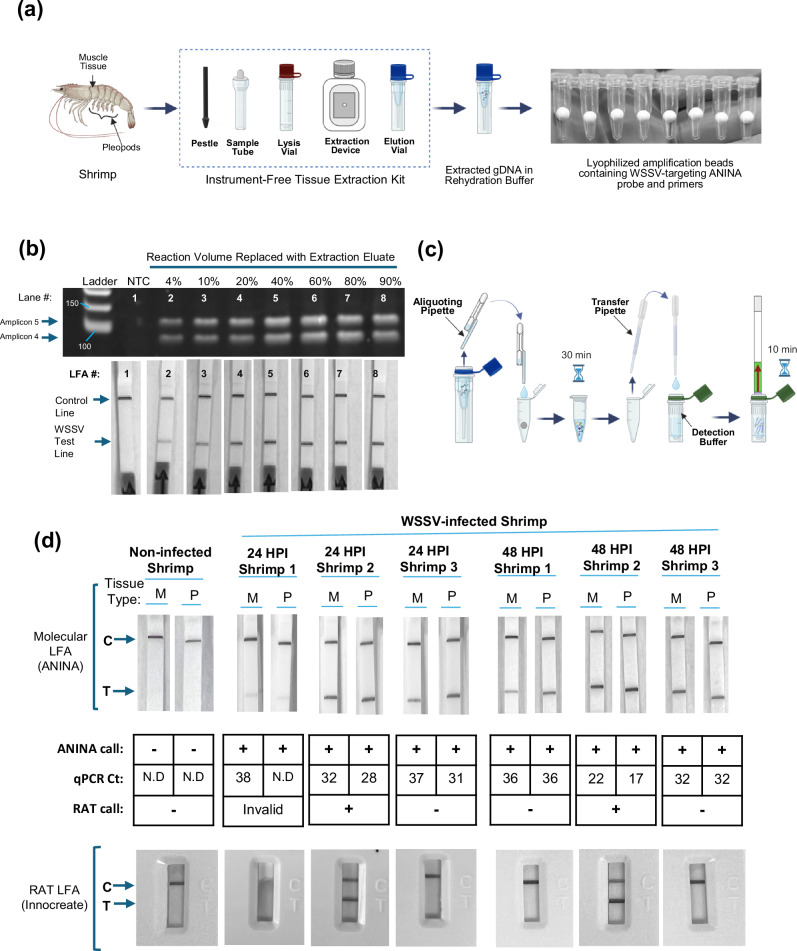
Instrument-free ANINA detection in a natural host model. **a** Schematic of shrimp sampling sites (muscle tissue and pleopods) and components of the instrument-free tissue extraction workflow. gDNA was eluted directly into ANINA pellet rehydration buffer and used to partially or fully rehydrate lyophilized ANINA beads containing WSSV-targeting oligonucleotides. **b** PAGE (top) and corresponding LFA (bottom) readouts from reactions rehydrated with increasing proportions of extracted WSSV-infected shrimp DNA, demonstrating direct coupling of simplified extraction to amplification and detection without increased nonspecific signal. Representative results are shown from two independent amplification experiments with similar results. A 50 bp DNA ladder was used as the molecular size marker in the PAGE gel and two reference sizes are indicated.  **c** Schematic of the integrated, instrument-free workflow. Tissue eluate (50 µL) was used to rehydrate a lyophilized WSSV bead, incubated for 30 min at ambient temperature, then diluted into detection buffer and applied to a LFA strip for visual readout after 10 min. **d** Representative LFA readouts from the ANINA molecular workflow (top) and a parallel rapid antigen test (RAT; bottom), alongside qualitative interpretation and qPCR results. Shown are the first of two independently processed technical replicates for each sample type. Invalid: control line absent; ND not detected by qPCR. LFA and gel images from all technical and biological replicates are shown in Supplementary Fig. 7. Corresponding qPCR results are provided in Supplementary Table 2. Source data are provided as a Source Data file. Schematics in (**a**) and (**c**) were created in BioRender. Roy, A. (2026) https://BioRender.com/5jygk7z. **a** additionally includes an original photograph of lyophilized amplification beads.

We next integrated lyophilized ANINA reagents with a 2-min, instrument-free tissue extraction workflow^43,44^ to establish a fully deployable sample-to-answer system (Fig. 7a). Shrimp tissue eluate was added directly to lyophilized WSSV-targeting ANINA beads, incubated at 25 °C for 30 min, then detected on lateral flow strips (Fig. 7a–c). Increasing proportions of eluate produced proportional increases in amplification yield and lateral flow signal without elevating nonspecific background, confirming compatibility between simplified extraction and ANINA assays (Fig. 7b).

We evaluated the integrated workflow using a natural WSSV infection model. Shrimp were infected via oral exposure to WSSV-positive tissue, recapitulating the natural route of transmission, and producing heterogeneous infection dynamics^45–48^. Pleopods, a preferred non-lethal sampling site^49^, and muscle tissue were collected from three shrimp each at 24 and 48 h post-infection (HPI) under field-mimicking conditions using only disposable components. The simplified workflow was used to process all the samples and directly rehydrate the lyophilized ANINA reactions, which were kept in ambient temperature (not incubated). After 30 min, the reaction was directly diluted into an optimized detection buffer without organic extraction and applied to LFA strips. The LFA results were visually recorded after 10 min, thereby keeping the total time under 45 min from sample input to test output.

To benchmark this molecular workflow against an established field test, the same shrimp were also tested using a WSSV rapid antigen test (Innocreate Biosciences®), which has been certified by World Organisation for Animal Health (WOAH) for field-based confirmatory diagnosis of WSSV clinical cases^50^. Infection status for both assays was interpreted blindly by visual assessment of test lines and subsequently confirmed by qPCR.

Consistent with heterogeneous infection dynamics, qPCR revealed a range of viral loads across samples (Supplementary Table 2), with four shrimp exhibiting low infection levels (< 10⁴ copies/µL) and two showing moderate-to-high infection (> 10⁴ copies/µL). ANINA detected all qPCR-positive samples, including low viral load specimens (20 copies per µL), with no false positives among non-infected shrimp controls (Fig. 7d and Supplementary Fig. 7a–d). In low-load samples where replicate qPCR measurements differed, ANINA produced more frequent positive calls, consistent with higher template input per reaction (Supplementary Fig. 7a–d and Supplementary Table 2). In contrast, the commercial rapid antigen test detected only moderate-to-high viral loads and failed to identify low-level infections (< 10⁴ copies/µL).

Additionally, in-silico cross-reactivity analysis of the WSSV ANINA oligonucleotide set against non-WSSV representative pathogens of common shrimp infectious diseases showed no coordinated complementarity across the complete set required for productive amplification (Supplementary Table 3). Only weak partial alignments with low query coverage and poor overall complementarity were observed for a subset of primers, whereas no similarity was identified for the AP, thereby supporting predicted assay specificity for WSSV.

Together, these data demonstrate that ANINA enables early, qPCR-comparable molecular detection within a rapid, fully instrument-free workflow suitable for field deployment.

## Discussion

Isothermal nucleic acid amplification methods offer advantages over qPCR for decentralized testing, yet broader adoption has been limited by trade-offs among specificity, assay complexity, equipment dependence, and operational robustness. Many approaches reintroduce workflow constraints that limit deployment in resource-limited environments where simplicity, stability, and interpretability are critical.

Here, we describe ANINA, a probe-guided recombinase-based framework that integrates amplification and detection within a single lyophilized reaction operating efficiently across a broad temperature range (20–45 °C) while staying between 5 and 30 min of assay time. By embedding spatial control of primer recruitment into the amplification mechanism, ANINA addresses key constraints of recombinase-based systems while expanding assay design flexibility.

A defining feature of ANINA is the active role of the AP. Rather than serving solely as a reporter, the AP organizes primer annealing through spatial annexation. Across multiple architectures, annexation enables efficient amplification using PPs as short as 10 nt and DPs as short as 15 nt, lengths substantially below those typically supported in recombinase-based systems. A single AP expands usable PP lengths from 10 to 35 nt and DP lengths from 15 to 35 nt while reducing dependence on primer thermodynamic parameters and retaining specificity at 25 °C within 30-min assay times. This likely occurs because PP extension generates a partially displaced amplicon intermediate that exposes the complementary strand for DP annealing without requiring AP-mediated recruitment.

Beyond amplification control, AP enables direct lateral flow detection without probe cleavage or auxiliary enzymatic processing, in contrast to other recombinase-based systems such as nfo-RPA and SHERLOCK (Table 3). This unified architecture supports detection of DNA viruses (EBV, WSSV), RNA viruses (RSV), and bacterial targets (*N. gonorrhoeae*) in the presence of abundant host genomic background, as well as multiplexed endpoint readout using orthogonally labeled primers. Unlike nfo-probes or SIBA-style invasion oligonucleotides, AP design is minimal and avoids reliance on internal modifications, requiring only recombinase-compatible length (29–40 nt), a 5′ detection label, and a 3′ block to prevent extension. Notably, AP label chemistry itself contributes to productive annexation beyond serving as a detection handle, although 5′ FAM-labeled APs consistently supported efficient amplification and lateral flow detection across all targets and temperature conditions. These features position ANINA as a generalizable amplification framework rather than a target-specific assay.

**Table 3:** Comparison of ANINA with representative recombinase-based isothermal amplification systems

Comparison Parameter	ANINA	RPA^14,25^	Nfo-RPA^12,23^	RPA + SHERLOCK^26,27^	SIBA^21,27–29^
**Assay architecture**	2 primers + 1 Annexing Probe (AP)	2 primers	2 primers + Nfo probe	2 primers + target-specific guide RNA + reporter probe	2 primers + Invasion oligonucleotide
**Operating temperature**^**a**^	20–45 °C	Typically 35–45 °C	Typically 35–45 °C	Typically 35–45 °C	Typically 40–45 °C
**Reaction time**^**a, b**^	5–30 min	5–30 min	10–30 min	~45–60 min	~30–60 min
**Primer length**	Supports 15–35 nt primers	Typically 30–35 nt primers	Same as RPA	Same as RPA	Supports 16–23 nt primers
**Direct endpoint LFA detection**	Yes	No	Yes	Yes	No
**Design requirements for LFA detection probe**	25–40 nt AP with 5′ detection label and 3′ block	N/A	≥45 nt Nfo probe with internal abasic site, 5′ detection label, and 3′ block	6–10 nt Reporter probe (5′ detection label and 3′ biotin label, non-target specific)	N/A
**Additional oligonucleotide design requirements**	None	None	None	Target-specific gRNA (~ 60–70 nt total; ~28 nt target-complementary region)	Invasion oligonucleotide (> 55 nt total; 5′ non-homologous seeding region, 3′ 2-O-methyl RNA region, 3′ block)
**Auxiliary enzymes required for LFA detection**	None	N/A	Endonuclease IV (nfo)	Cas13a + T7 transcription	N/A
**LFA detection mechanism**	Direct AP-associated detection	N/A	Probe cleavage-dependent	Collateral reporter cleavage	N/A
**Probe contributes to reaction specificity**	Yes	N/A	No	No	N/A
**Analytical sensitivity demonstrated**	Attomolar to low-copy detection	Attomolar to low-copy detection reported	Attomolar to low-copy detection reported	Attomolar to low-copy detection reported	Low-copy detection reported
**Equipment required**	Does not require incubator	Typically requires incubator	Typically requires incubator	Typically requires incubator	Yes
**Compatible detection modalities**	LFA, fluorescence, imaging	Fluorescence, imaging	LFA, fluorescence, imaging	LFA, fluorescence, imaging	Fluorescence, imaging

^a^Operating conditions and time ranges represent typical assay conditions reported in the literature and/or demonstrated in this study.

^b^Amplification time may vary with reaction temperature and assay conditions.

ANINA’s modular architecture supports both instrument-free endpoint detection and real-time quantitative readout. In a fluorescence format, qANINA exhibits log-linear quantification across multiple orders of magnitude and substantially reduced detection times compared to matched qPCR assays, reflecting the absence of thermocycling constraints. These results demonstrate that ANINA can be easily integrated across laboratory and near-point-of-care workflows without sacrificing analytical performance.

ANINA’s integration into a fully equipment-free, 45-min sample-to-answer workflow for point-of-care detection of WSSV infection in shrimp further demonstrates its translational feasibility in a biologically and operationally relevant diagnostic setting. WSSV, the causative agent of White Spot Disease, is a highly virulent pathogen recognized by WOAH as a major threat to global aquaculture. Infection spreads rapidly within shrimp ponds and can result in near-complete mortality within 3–7 days, creating a narrow window for detection and intervention^49,51,52^. Although laboratory-based qPCR assays can detect WSSV as early as 24 h post infection (HPI), their deployment is constrained by centralized infrastructure and sample transport, delaying actionable results in field settings^52^. In contrast, existing rapid antigen tests typically lack sensitivity during early infection stages (< 48 HPI), limiting timely outbreak-control measures.

In shrimp infected under natural exposure conditions, the integrated sample-to-answer workflow using ANINA reliably detected early stages (24–48 h) of WSSV infection with sensitivity comparable to reference qPCR and improved performance relative to a commercial antigen test. This validation using naturally infected host samples under biologically realistic conditions—including crude tissue-derived nucleic acids, heterogeneous infection dynamics, lyophilized reagents, and ambient incubation—highlights practical deployability and alignment with the World Health Organization ASSURED framework for point-of-care diagnostics^1–4^. Diagnostic performance analysis using qPCR as the reference standard showed concordance between the integrated ANINA workflow and laboratory-based detection (Supplementary Table 2), although the limited cohort size currently supports only descriptive performance evaluation. Future work will involve experimental exclusivity testing against related shrimp pathogens, reproducibility, and larger-scale validation studies across independent field cohorts and broader environmental conditions.

Multiple observations distinguish annexation from established recombinase-based mechanisms. Standard primer concentrations were used and identical primer sets failed in the absence of an AP, which exclude primer overloading as an explanation. Systematic variation of primer length and position demonstrate amplification efficiency is governed primarily by the spatial distance between the 5′ ends of the AP and PP rather than primer thermodynamics alone. Increasing AP–PP separation reduces amplification and AP label chemistry influences annexation efficiency. Comparable amplification behavior across multiple enzyme suppliers indicates ANINA is orthogonal to enzyme source (Supplementary Fig. 8).

Lateral flow signal was observed only in target-containing reactions and persisted after enzyme removal, arguing against nonspecific aggregation or residual protein-mediated capture. Successful LFA detection was also achieved by direct application of unpurified WSSV amplification products to lateral flow strips using an optimized PBS-based detection buffer, demonstrating high-sensitivity detection can be achieved without organic extraction (Fig. 7). APs remain 3′-blocked and are not incorporated into amplicons, excluding probe cleavage or extension as contributing mechanisms.

Detection under conditions preserving non-canonical structures is consistent with formation of a higher-order, sequence-dependent AP-amplicon association potentially stabilized by steric, ionic, or protein-mediated interactions^37–41^. Although PAGE analyses predominantly showed discrete full-length amplicons, occasional higher-molecular-weight species were observed under conditions associated with accelerated amplification kinetics, including elevated template concentrations and temperatures. These species may represent transient partially extended or branched amplification intermediates generated during overlapping isothermal amplification cycles. The contribution of AP label chemistry to annexation efficiency further suggests productive annexation depends not only on sequence complementarity but also on the local structural and physicochemical properties of the AP–target complex. We hypothesize 5′ labels may influence recombinase loading, duplex accessibility, or local steric and electrostatic interactions that facilitate productive PP recruitment.

Collectively, these findings support annexation as a spatially organized primer recruitment mechanism distinct from established recombinase-assisted strand invasion or probe-cleavage strategies. We propose annexation begins when the recombinase-loaded AP invades the target duplex to generate a locally accessible AP-bound heteroduplex region adjacent to the PP-binding site. The AP-associated local environment may be further stabilized by label-dependent steric or physicochemical interactions that facilitate productive PP annealing and extension by the strand-displacing polymerase. This model explains why AP–PP proximity strongly influences amplification efficiency, shortening the PP can preserve amplification when AP proximity is maintained, and increasing AP–PP distance reduces efficiency even when primer length is unchanged. Further structural elucidation of annexation and the role of probe label chemistry will be investigated in future work.

Current limitations include the need for target-specific oligonucleotide design, broader validation of higher-order multiplexed amplification, and further mechanistic characterization of annexation. Although this study demonstrates feasibility of multiplexed endpoint detection and sensitivity to terminal primer mismatches, future work will evaluate higher-order multiplexing, tolerance to internal sequence variation, and performance across diverse pathogen strains and field settings. Broader inter-laboratory validation will be required to support large-scale deployment. Validation using clinical samples involving human pathogens is ongoing.

As with other high-sensitivity isothermal amplification systems, ANINA may remain susceptible to nonspecific amplification and carryover contamination under inappropriate assay or workflow conditions. In the present study, no detectable amplification was observed in no-template controls across the evaluated conditions, consistent with AP-guided spatial regulation contributing to low background amplification. Standard contamination-control practices, including closed-tube lyophilized reactions and physical separation of pre- and post-amplification workflows, remain important for minimizing carryover contamination risk. To facilitate adoption, standardized lyophilized reagents are available for external testing, and computational design tools encoding annexation-specific primer rules are under development.

In summary, ANINA introduces unified probe-regulated, recombinase-based amplification and detection within a simplified and generalizable architecture. By expanding primer design space and reducing reliance on instrumentation and cold-chain logistics, ANINA provides a scalable, cost-effective, and robust molecular diagnostic framework that supports deployment in decentralized and resource-limited settings while also offering opportunities to simplify existing laboratory workflows.

## Methods

### Ethical statement

Specific pathogen-free (SPF) Pacific whiteleg shrimp (*Litopenaeus vannamei*; juvenile, 1–5 g) were purchased from authorized vendors (Oceanic Institute of Hawaii Pacific University and Homegrown Shrimp USA) and shipped to Seek Labs. Shrimp importation and research use were authorized under a Utah Division of Wildlife Resources Certificate of Registration (COR No. 4POSS10845; effective 02/04/2022–02/03/2027). Husbandry and biosafety practices followed Utah state regulations, guidance from the USDA-accredited Aquatic Animal Health Laboratory (Florida Atlantic University), published literature^45–49^, and procedures reviewed by Seek Labs’ internal Quality and Safety Committee. Shrimp are invertebrates and are not regulated under the U.S. Animal Welfare Act; therefore, Institutional Animal Care and Use Committee approval was not required. Procedures for handling and disposal of WSSV-infected materials were reviewed by the Utah Department of Natural Resources Division of Wildlife Resources (DWR Fisheries Program). Shrimp sex was not determined and was not considered a biological variable.

De-identified human saliva samples used as background matrix for virus-spiked experiments were obtained from commercial vendors (Precision Biospecimen Solutions, Inc. and Innovative Research, Inc.). Samples were originally collected under vendor IRB-approved protocols with informed consent or IRB-approved waivers permitting secondary research use. Race, ethnicity, and other demographic metadata were not available or recorded. Because all samples were de-identified and no identifiable private information was accessible to the investigators, this work did not constitute human subjects research. All specimens were handled in accordance with applicable biosafety requirements and Seek Labs’ Quality Management System and Operational Safety Plan.

### Oligonucleotide design and sequences

All primers and probes used in this study were designed manually using molecular biology tools, SnapGene or ApE, and synthesized by Integrated DNA Technologies (IDT). Schematics showing ANINA architecture in Figs. 2a, e, 3a, 4a, c, and Supplementary Fig. 2a were created on Microsoft PowerPoint. AP, PP, and DP targeting the VP19 gene of WSSV were designed using consensus sequences generated by multiple sequence alignment (MAFFT) of representative WSSV genomes from NCBI, using the VP19 reference sequence (DQ902655.1). APs were designed to meet recombinase-loading requirements (≥ 29 nt) and positioned to spatially recruit adjacent primers. PP and DP sequences were intentionally varied in length and thermodynamic properties to evaluate annexation effects. All oligonucleotides were screened using NCBI BLAST to ensure broad strain coverage and to minimize cross-reactivity with host genomes.

Consensus-based oligonucleotide sets were similarly designed for the *BHRF1* gene region of EBV and the TIGR00645 family protein region of *Neisseria gonorrhoeae*. Oligonucleotides targeting RSV were designed against the *M* gene of the RSV A Long strain (ATCC VR-26). Computational design tools encoding annexation-specific primer rules are under development.

All ANINA and qPCR oligonucleotide sequences with their corresponding chemical modifications are provided in Tables 1 and 2. Oligonucleotide lengths in nucleotides (nt) refer to target-complementary bases and exclude terminal modifications. All expected amplicon sizes in base pairs (bp) for ANINA are provided in Supplementary Table 1. GC percentage, melting temperature (Tm), predicted folding energy (ΔG), and secondary structure melting temperatures (2° Tm) were calculated using IDT OligoAnalyzer under SpecSheet conditions for ANINA oligonucleotides and predicted ANINA amplicons (DNA target, 0.4 µM oligonucleotide concentration, 50 mM Na⁺, 17 mM Mg²⁺, 0.7 mM dNTP) and under qPCR default parameters for the qPCR oligos. Thermodynamic parameters are shown for comparative purposes and were not used as strict design constraints. Primer efficiency was verified using reference qPCR assays or by sequence analysis of amplicons verified using Nanopore sequencing (Plasmidsaurus).

To evaluate potential cross-reactivity of the WSSV ANINA oligonucleotide set used in the integrated sample-to-answer assay, NCBI nucleotide BLAST analysis was performed against the intended target pathogen (WSSV), target host (shrimp, *Litopenaeus vannamei*), and representative causative pathogens of common shrimp infectious diseases, including AHPND (*Vibrio* spp.), DIV1/SHIV, EHP, IHHNV, IMNV, NHP, TSV, and YHV (Supplementary Table 3). BLAST parameters were set to search standard databases and the program was selected to optimize for highly similar sequences (megablast). For each primer–accession comparison, the highest-ranking alignment was selected based on the lowest *E*-value and highest Max Score. Max Score, Total Score, *E*-value, Percent Identity, and Query Coverage from this representative alignment were recorded for downstream analysis. When multiple accessions were evaluated for a given organism, the strongest off-target alignment identified among all accessions was retained to represent the maximum potential cross-reactivity for that organism.

### Sample preparation and nucleic acid extraction

WSSV-infected (E-LT2002 isolate) shrimp tissue was either obtained from the Aquatic Animal Health Laboratory (AAHL) at Florida Atlantic University-Harbor Branch or from shrimp infected using protocol described here and stored in −80 °C until use.

Human gammaherpesvirus 4, commonly known as EBV (ATCC, VR-1492), Human RSV A strain Long (ATCC, VR-26), and *Neisseria gonorrhoeae* (Zopf) Trevisan strain F62 (ATCC, BAA-1837) were purchased as frozen stocks and stored in liquid nitrogen before use. Deidentified human saliva collected from healthy donors was purchased from Precision Biospecimen Solutions Inc. and Innovative Research (IRHUSL5ML). Pooled human saliva was used as a negative human matrix to generate contrived EBV and RSV samples by adding thawed virus directly to saliva (1:5).

*N. gonorrhoeae* was cultured in Fastidious Broth (ThermoFisher Scientific, R07664) at 37 °C in a 5% CO_2_ incubator for 48 h following manufacturer instructions. Culture (1 mL) was pelleted by centrifugation and resuspended in simulated human urine (Biochemazone, BZ404) to create contrived *N. gonorrhoeae* samples.

For standard laboratory assays, gDNA was extracted from ~20 to 25 mg of shrimp muscle tissue using the DNeasy Blood and Tissue Kit (Qiagen, 69504) according to the manufacturer’s protocol. DNA templates from EBV-spiked saliva and *N. gonorrhoeae*-spiked urine were extracted using the Monarch Genomic DNA Purification Kit (New England Biolabs, T3010). RNA templates from RSV-spiked saliva were extracted using the Monarch Total RNA Miniprep Kit (New England Biolabs, T2010). Manufacturer-recommended protocols specific to each sample matrix were followed.

Unless otherwise specified in the Figure legends, all amplification reactions contained ~10⁶ copies of target template per reaction. Extracted nucleic acids from blank matrices were used as negative background controls. All pathogen handling complied with institutional BSL-II guidelines and ATCC recommendations.

### Shrimp natural host infection model

SPF Pacific whiteleg shrimp (juvenile, 1–5 g) were obtained from Oceanic Institute of Hawaii Pacific University or Homegrown Shrimp USA and maintained in 20–40-gallon saltwater tanks under controlled laboratory conditions^45–49^. WSSV infection was established by oral exposure through feeding minced WSSV-infected shrimp tissue (100 mg tissue per gram live shrimp weight) and harvested by following previously described protocols^45–48^. Briefly, shrimp were collected from control tanks or at the indicated time points post infection and euthanized by hypothermia in ice water followed by cervical dislocation. Pleopods and de-shelled muscle tissue were collected, processed immediately, or stored at −80 °C until use. All handling of WSSV-infected samples was performed under institutional BSL-II conditions.

### Reference amplification assays

#### Reference PCR assay

PCR primer screening experiments were performed using Brilliant III Ultra-Fast SYBR Green Master Mix (Agilent, 600883) on an Agilent AriaMx 3000 real-time PCR instrument following manufacturer instructions.

#### Reference RPA assay

ANINA primer functionality was also tested with TwistAmp® Basic, a commercial RPA kit (TwistDx, TABAS03KIT) (Supplementary Fig. 4b) using manufacturer-recommended protocol but at a lower incubation temperature (25 °C).

### qPCR quantification assays

All qPCR assays were performed on an Agilent AriaMx 3000 real-time PCR instrument following manufacturer instructions. Cycling conditions consisted of 40 cycles of 95 °C for 5 s followed by 60 °C for 10 s, with fluorescence acquisition during the annealing/extension step.

#### WSSV quantification assay

WSSV plasmid standard was generated by amplifying the VP28 gene and cloning it into pCR2.1-TOPO using TOPO TA cloning kit (Thermo Fisher Scientific, K450001) following manufacturer protocol. The extracted plasmid (Promega Wizard Miniprep kit) was sequence verified and used to create serial dilutions ranging from 10^7^ to 10^1^ copies/µL, which were then used for WSSV quantification in infected shrimp using Brilliant III Ultra-Fast Master Mix (Agilent, 600880). The assay also incorporated a host control to verify extraction and assay consistency. The qPCR assay performance was verified using a commercial WSSV qPCR kit (Creative Biogene, MBSK-ZR311).

#### EBV quantification assay

Extracted EBV gDNA was used to create serial dilutions ranging from 10^6^ to 10^0^ copies/µL, which were then used for quantification of extracted EBV templates using Brilliant III Ultra-Fast SYBR Green Master Mix (Agilent, 600883). The qPCR assay performance was verified using a commercial EBV qPCR kit.

### ANINA reaction chemistry

All ANINA assays were performed using Seek Amplification™ chemistry (Seek Labs), a recombinase-based formulation optimized for robust performance across ambient temperatures and compatible with both liquid and lyophilized formats. The enzymatic components included the following: T4 UvsX recombinase; UvsY mediator protein; T4 GP32 single-stranded DNA binding protein; Bsu large fragment or an equivalent strand-displacing DNA polymerase lacking 5′ → 3′ and 3′ → 5′ exonuclease activity; and, where indicated, *E. coli* RNase H and M-MuLV–based RT. Functionally equivalent commercial enzymes from multiple suppliers (including Intact Genomics, Watchmaker Genomics, and Thermo Fisher Scientific) were found to perform comparably in ANINA reactions under identical reaction conditions, suggesting the source-agnostic nature of the formulation (Supplementary Fig. 8 and Supplementary Table 4).

The reaction mix also contained ATP, dNTPs, crowding agents (polyethylene glycol), stabilizers (trehalose), reducing agent (DTT), Tris acetate (pH 7.9), potassium acetate, magnesium acetate, and an ATP regeneration system (creatine kinase and phosphocreatine). To ensure reproducibility across targets, temperatures, and detection modalities, all experiments—unless otherwise specified—were performed using a single standardized formulation using reaction components sourced from Intact Genomics (UvsX, UvsY, GP32, Bsu), Thermo Fisher Scientific (ATP, RNase H, RT), and New England Biolabs (dNTPs), with all remaining reagents from MilliporeSigma (Supplementary Table 4). The standardized formulation in lyophilized format is available for external validation.

Primer concentrations in reactions were maintained at 420 nM for each primer and APs were used at 120 nM, unless otherwise indicated.

#### Liquid-format annexing amplification

Liquid-format reactions were assembled in 0.2 mL PCR tubes on ice or a cold block. Unless otherwise specified, standard reactions were performed at a final volume of 25 µL and incubated for 30 min at 25 °C in a temperature-controlled incubator (Benchmark). Reactions were prepared by combining pre-mixed reaction buffer components, enzyme mixture, assay-specific oligonucleotides, and template DNA or RNA, followed by initiation with magnesium acetate as the final step. For experiments involving multiple samples, master mixes were prepared prior to aliquoting to minimize pipetting variability. Reaction tubes were capped, briefly mixed, centrifuged, and incubated for 30 min at 25 °C in a temperature-controlled incubator.

#### Lyophilized annexing amplification

Lyophilized reactions were prepared using glycerol-free enzyme stocks. All reaction components, except Tris acetate, potassium acetate, and magnesium acetate, were combined in individual 0.2 mL tubes, frozen, and lyophilized overnight using a FreeZone 4.5 L freeze dryer (Labconco). Larger batches were manufactured as bead-format pellets by Argonaut Manufacturing Services using the same formulation. These lyophilized beads are available on request for external testing.

Lyophilized pellets were prepared either with pre-loaded assay-specific oligonucleotides or as primer-free formulations and stored at ambient temperature in sealed, light-protective packages. Pellets retained full amplification activity for at least 12 months. Prior to use, pellets were rehydrated with rehydration buffer (Tris and potassium acetate), template nucleic acid, oligonucleotides (if not pre-loaded), and magnesium acetate, then gently mixed and incubated under the specified conditions. All experiments used 50 µL lyophilized reactions. Lyophilized pellets in bead form are available on request.

### End-point detection and gel analysis

Unless stated otherwise, amplification reactions were terminated by mixing an equal volume of phenol–chloroform–isoamyl alcohol (25:24:1, MilliporeSigma, 77617). Samples were vortexed briefly and centrifuged at 10,000 × *g* for 1 min. The aqueous phase was recovered for downstream lateral flow or gel analysis. All uncropped images of lateral flow assay strips and gels, along with corresponding analyses, are included in the Source Data file accompanying this paper.

#### Lateral flow assay (LFA)

Endpoint detection was performed using Hybridetect lateral flow strips (Milenia Biotec, MGHD 1 and MGHD2 1) containing one (1 T) or two (2 T) test lines. For standard assays, 10 µL of extracted aqueous phase was diluted into 100 µL Milenia detection buffer prior to strip insertion. For the instrument-free workflow, amplification reactions were diluted directly without organic extraction or purification into an optimized PBS-based detection buffer without cleanup. The PBS buffer reduced background signal in template-negative reactions while preserving positive signal intensity. Test strips were removed from solution and imaged after 15 min using a smartphone camera or gel imaging system.

#### Gel electrophoresis and imaging

For polyacrylamide gel electrophoresis (PAGE), cleaned up post-amplification samples (5 µL) were mixed with 6× loading dye (New England Biolabs, B7025S) and resolved on 10% TBE precast PAGE gels (Thermo Fisher Scientific, EC62755BOX) using an XCell SureLock Mini gel chamber (Thermo Fisher Scientific, EI0001), run at 175 V for 45 min in 1× TBE running buffer. DNA ladders (0.2 µg/lane, New England Biolabs, N3236L) were included in all gels for size estimation. For the experiment in Supplementary Fig. 3c, the gel was run with chilled running buffer (temperature ~4–10 °C) and at lower voltage (80 V) for 3 h, following previously published protocol^42^.

Following electrophoresis, gels were imaged using a gel imaging system (UVP GelStudio). Images were first acquired using a fluorescence filter (513 nm excitation, 557 nm emission) to visualize FAM-labeled oligonucleotides. Gels were then post-stained with 3× GelRed nucleic acid stain (Biotium, 41003) for 1 min, rinsed with water, and imaged under UV illumination using a standardized acquisition protocol.

#### Image analysis

Unless stated otherwise, all gel and LFA images within each figure panel were derived from the same image, non-relevant gel lanes or LFA strips were cropped for clarity and consistency, non-adjacent figure sections show clear separation in figures, and sections were not reordered. All gel images had one or more molecular markers. All raw images are included in supplementary files.

Densitometric analysis of gel and LFA images was performed using VisionWorks software (Analytik Jena). Regions of interest (ROIs) corresponding to specific amplicon, ladder bands, or test lines were defined, and local background signal was subtracted. ROI position and size were determined based on the migration distance and expected band size of positive controls. Mean band density of each amplicon band on gel was normalized to ladder bands within the same gel to account for inter-gel variability, while band densities of LFA test lines were normalized to negative template control LFA. Reported values represent the average relative mean density across replicate experiments and were used for quantitative comparison of amplification yield. No data points were excluded from analysis unless explicitly stated in figure legends.

All data analysis was carried out in Microsoft Excel or GraphPad Prism 11.0.1.

### qANINA real-time fluorescence assays

For quantitative real-time ANINA (qANINA), EvaGreen DNA-binding dye (Biotium, 31000) was included at 1× concentration during rehydration of lyophilized pellets. Reactions were split into two technical replicates before adding template (final volume of 25 µL each) and run on an Agilent AriaMx 3000 instrument maintained isothermally at 39 °C. Fluorescence was recorded at 10-s intervals using a SYBR/FAM-compatible detection channel without thermal cycling. For qPCR and qANINA comparisons, identical serial dilutions of extracted viral gDNA were used to generate standard curves.

#### Data analysis for real-time assays

For qANINA and qPCR experiments, fluorescence data were analyzed using Agilent Aria software (version 2.1) to determine cycle threshold (Ct) values. For qANINA, detection time (Dt) was defined as the elapsed reaction time required for fluorescence to exceed a fixed threshold (ΔR = 100) above baseline. This threshold was selected such that no-template controls (NTCs) remained below threshold throughout the acquisition window.

For qPCR comparisons, Ct values were converted to Dt using a cycle duration of 1 min per cycle, consistent with manufacturer specifications and instrument run time, and doubling time was calculated analogously.

Standard curves were generated by plotting Dt (or Ct) versus log₁₀(N₀), where N₀ represents the initial template copy number per reaction. Error bars represent standard deviation of technical or biological replicates, as indicated in figure legends. Linear regression was performed on Microsoft Excel using ordinary least squares to fit:

*Dt* = *m* · log₁₀(N₀) + b

To keep standard-curve slope estimates comparable across methods, we fit slopes over the shared dynamic range; therefore, the single-copy point (detected only by qANINA) was excluded from slope fitting.

All data analysis was carried out in Microsoft Excel or GraphPad Prism 11.0.1.

#### Doubling-time calculation

Assuming exponential amplification kinetics, the number of amplified copies (*N*) at a specific time point (*t*) is:

*N*(*t*) = *N*₀ · 2^(*t*/Td)

where Td is the effective doubling time and *N*₀ is the number of target template copies at the start of reaction (*t* = 0).

At threshold crossing, the amplified copy number (N_threshold_) is constant across reactions.

Therefore:

*N*_threshold_ = *N*₀ · 2^(Dt/Td)

where *N*_threshold_ corresponds to ΔR = 100 under the assay’s fluorescence normalization.

Taking log₁₀ of both sides:

log₁₀(*N*_threshold_) = log₁₀(*N*₀) + (Dt/Td) · log₁₀(2)

Rearranging:

Dt = [log₁₀(*N*_threshold_)–log₁₀(*N*₀)] · Td/log₁₀(2)

Because log₁₀(*N*_threshold_) is constant across reactions, the slope (*m*) of Dt versus log₁₀(*N*₀) is:

*m* = −Td/log₁₀(2)

Thus, the effective doubling time was calculated as:

Td = −*m* · log₁₀(2)

Because Dt decreases with increasing *N*₀, *m* is negative. Td values obtained from regression (in minutes) are reported as a positive value and converted to seconds by multiplying by 60. All regression analyses and calculation files are provided in the Source Data.

### Simplified sample-to-answer molecular assay workflow

#### Instrument-free extraction workflow

For simplified workflow experiments (Fig. 7), shrimp tissue was processed using a 2-min, instrument-free extraction workflow containing single-use components^43,44^. The workflow consists of a Seek Extraction^TM^ (Velox) device, plastic pestle, sample vial, lysis buffer vial, and elution buffer vial (Fig. 7a). The workflow consisted of mechanically disrupting shrimp tissue using the pestle (~ 30 s) in the sample vial and mixing with lysis buffer. The sample vial is squeezed to filter out the lysate through the vial cap (containing a pre-filter) and then is transferred to the extraction device, where it interacts with a binding membrane before it is finally poured out and discarded. The elution buffer was subsequently added to the same extraction device and recovered as the final eluate.

#### Integrated instrument-free amplification and detection

For the complete instrument-free workflow (Fig. 7c), all steps were performed using disposable consumables without any powered instrumentation. Shrimp were collected at 24- and 48-h post-infection (HPI), and both muscle tissue and pleopods were collected and processed immediately. The first two pleopods of each shrimp were processed as the first replicate, while the next two pleopods were used as the second replicate. Two muscle tissue chunks cut with a disposable scalpel from the first segment of each shrimp were processed as duplicate samples. Replicates were also processed by different users.

Additionally, non-infected shrimp and no-template/positive-template controls (NTC/PTC) were run in parallel. During the 24 HPI workflow experiment, the NTC/PTC reactions processed after infected shrimp samples showed evidence of carryover contamination, as indicated by PAGE and LFA positivity (Supplementary Fig. 7c), and were excluded from interpretation. In the subsequent 48 HPI workflow experiment, NTC/PTC reactions were prepared prior to infected sample processing and remained negative throughout the workflow. All corresponding raw images are provided in the Source Data.

The ANINA rehydration buffer used to elute the gDNA also incorporated Mg acetate. A 50 µL single-use aliquoting pipette was used to transfer the eluate to the amplification tube and rehydrate the lyophilized bead (WSSV-specific). The tubes were mixed by gentle tapping and incubated on the lab benchtop (~ 20–25 °C) for 30 min. A part of each post-amplification reaction was pipetted out for gel analysis (for result verification only, not required for field assays). Using a transfer pipette, the remaining post-amplification reaction was transferred into the detection buffer. LFA strips were added immediately, and test lines were visually assessed and imaged after 10 min.

In parallel, gill samples were isolated from each shrimp and used to conduct the WSSV RP rapid test (InnoCreate Biosciences, P11-01-01-020) in duplicates following manufacturer-recommended instructions. The extracted shrimp samples were later analyzed by qPCR, and infection levels were quantified as genomic copies/µL eluate. Shrimp-level calls were determined by majority vote across replicate measurements. For qPCR and ANINA, four replicate measurements were available per shrimp (two tissues × two extraction replicates); for RP rapid test, two replicate measurements were available per shrimp. A shrimp was classified as positive if ≥50% of replicate measurements were positive ( ≥ 2/4 for qPCR/ANINA; ≥1/2 for RAT).

### Statistics and reproducibility

Randomization was not used because experimental conditions were defined a priori (e.g., primer/probe variants, amplification conditions, and target input concentrations). No statistical methods were used to predetermine sample size. Sample sizes, replicate numbers, and the nature of the replicates are indicated in the corresponding figure legends. For shrimp infection studies, lateral flow/antigen test results were interpreted qualitatively without prior knowledge of qPCR results. Blinding was not used for other assay-development experiments. Experiments were repeated independently as indicated throughout the paper. Representative gel and lateral flow images are shown and independent replicate experiments yield similar qualitative results. No data were excluded from analysis unless explicitly stated in the corresponding figure legend or “Methods” section.

### Reporting summary

Further information on research design is available in the Nature Portfolio Reporting Summary linked to this article.

## Supplementary information

Supplementary Information

Description of Additional Supplementary Information

Supplementary Data 1-7

Reporting Summary

Transparent Peer Review file

## Source data

Source Data

## Data Availability

All data supporting the findings of this study are available within the Article, Supplementary Information, and Source Data file. The Excel version of the Tables 1–3 and Supplementary Tables 1–4 are also available in the Supplementary Data file. The Raw data underlying all figures and tables are provided in the Source Data file accompanying this paper and include raw quantitative outputs, uncropped gel and lateral flow assay images, qPCR and qANINA fluorescence measurements, and associated analyses. Source data are provided with this paper. Lyophilized Seek Amplification™ beads and other proprietary materials described in this study are available from the corresponding author through Seek Labs Inc. upon request, subject to applicable material transfer agreements and intellectual property considerations. Source data are provided with this paper.
