# Supplementary material for: Annexing isothermal nucleotide amplification enables probe-guided amplification and direct detection at ambient temperature
Source: Nat Commun. 2026 Jul 14;17:8638. doi: 10.1038/s41467-026-75361-6 (PMC13486510; doi:10.1038/s41467-026-75361-6)
Supplement: Supplementary file 1 [file 41467_2026_75361_MOESM1_ESM.pdf]

Annexing Isothermal Nucleotide Amplification Enables Probe-guided Amplification and Direct Detection at Ambient Temperature

Authors: Ariana Human-McKinnon[1], Madisen Pyper[1], MiKenzee Frazier[1], Emily Plant[1], Harrison Piper[1], Tyler Archibald[1], Anindita Roy[1,*]

[1]Seek Labs, Inc. 350 W 800 N, Salt Lake City, UT 84103, United States

*Corresponding Author: annie@seeklabs.com

Supplementary Figures 1-8: Page 2-9

Supplementary Tables 1-4: Page 10-13

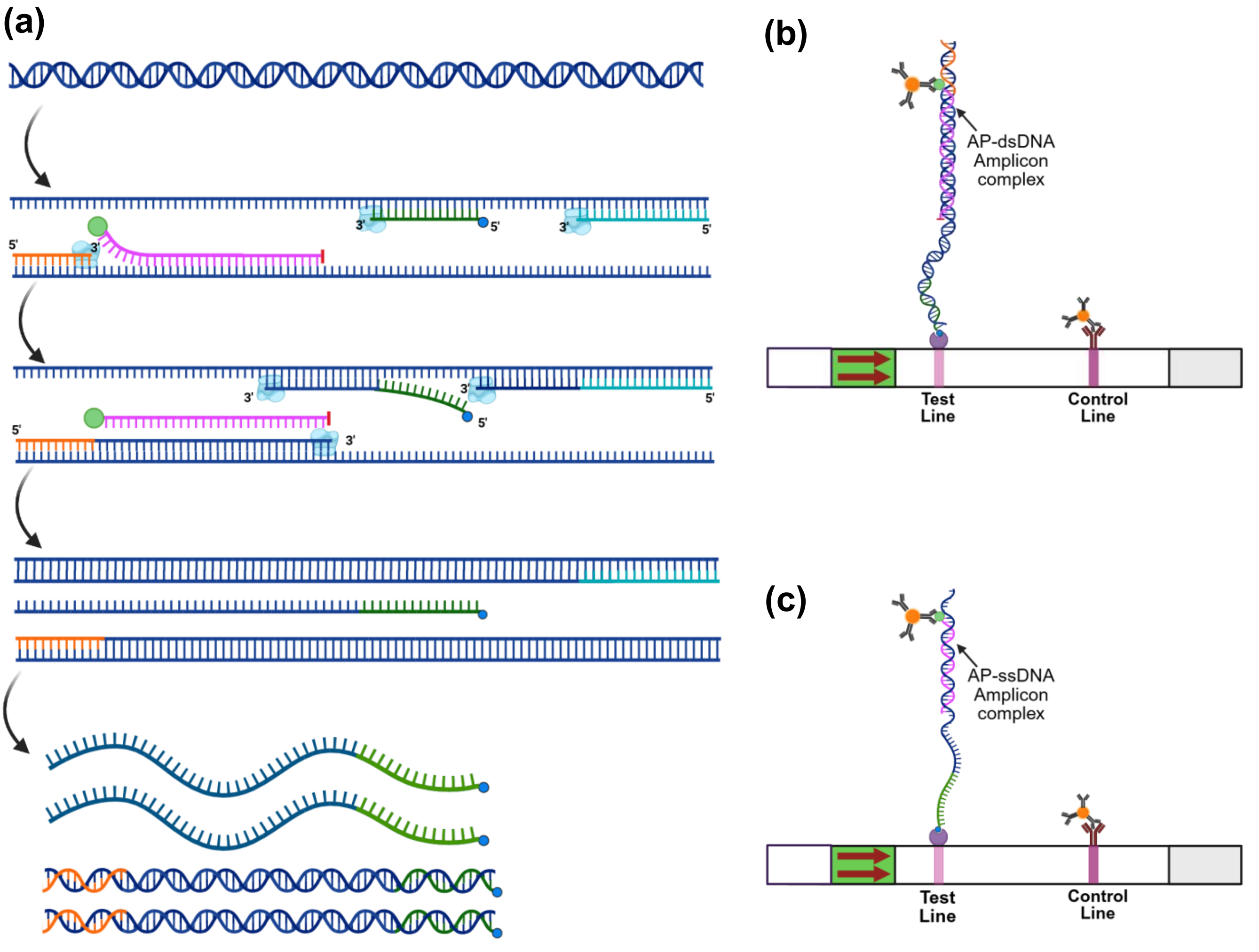

Supplementary Figure 1 | Schematic of Asymmetric, Semi-nested ANINA and LFA detection.

(a) In contrast to using only one DP as shown in Fig. 1, two DPs are used to create a semi-nested conformation with PP. The reaction proceeds in the same manner as described in Fig. 1a and leads to amplification of biotinylated dsDNA amplicons. However, the second DP creates a longer dsDNA amplicon that displaces the strand amplified from the first biotin-labelled DP, leading to accumulation of biotinylated ssDNA amplicons while also serving as template for additional biotinylated dsDNA amplicons. The LFA test line detects both (b) the AP-dsDNA amplicon complex and (c) the AP-ssDNA complex, leading to more intense detection.

Figure created in BioRender. Roy, A. (2026) https://BioRender.com/5jygk7z

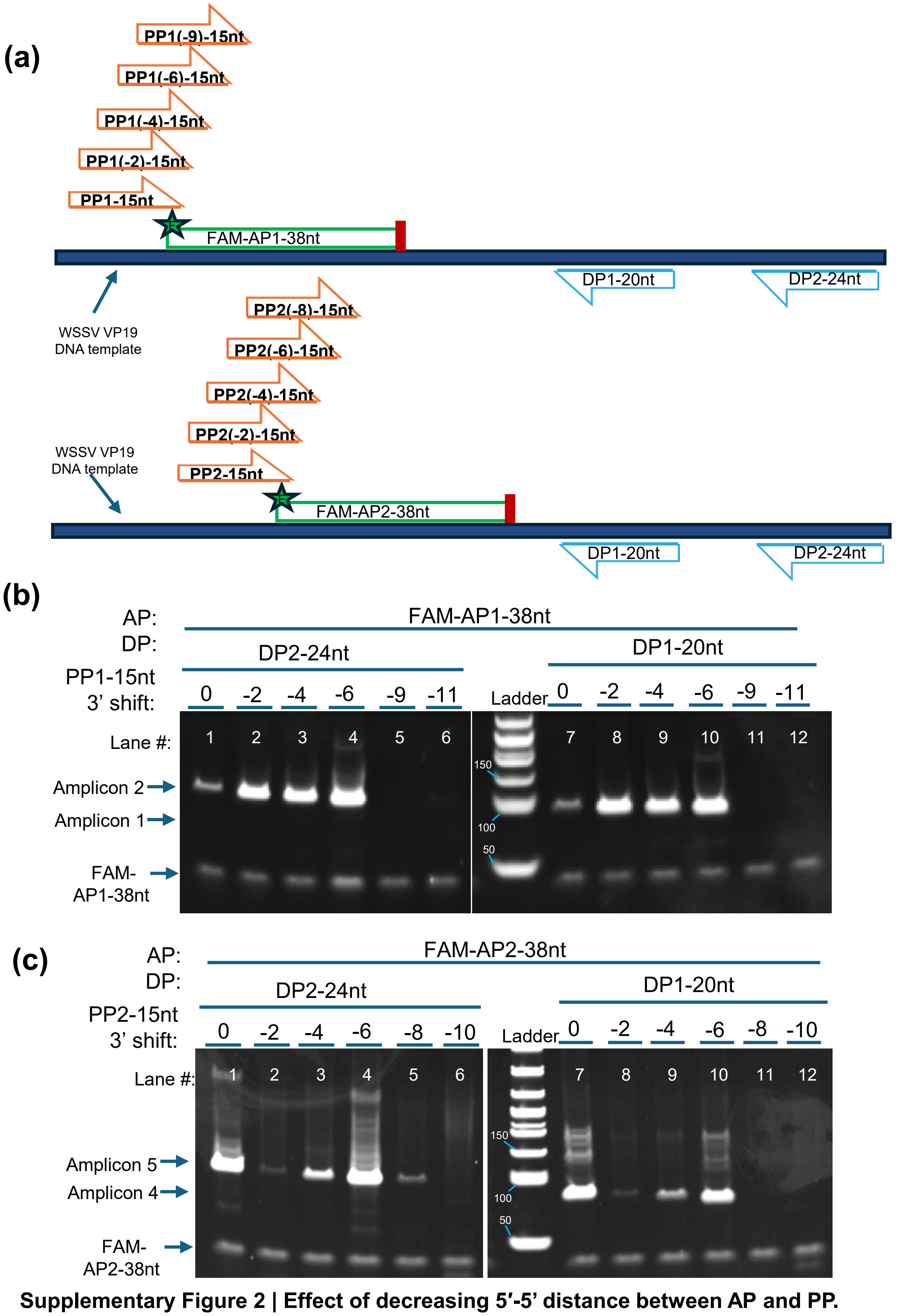

Supplementary Figure 2 | Effect of decreasing 5′-5′ distance between AP and PP.

(a) Schematic of PP1 and PP2 variants of constant-length (15 nt) primers shifted 3' along the target sequence. PAGE analysis of ANINA amplification using 3′-shifted (b) PP1 variants in presence of FAM-AP1-38nt, and (c) PP2 variants in the presence of FAM–AP2–38 nt, revealing dependence of annexation efficiency on AP–PP spatial positioning. Amplification was retained with both shorter DPs up to a 6-nt 3′ shift for both primer sets. Only PP2(-8) amplified with DP2-24nt; the other PPs did not amplify after 8th/9th nt shift. Representative results are shown. All amplification conditions were evaluated in at least two independent experimental runs with similar results.

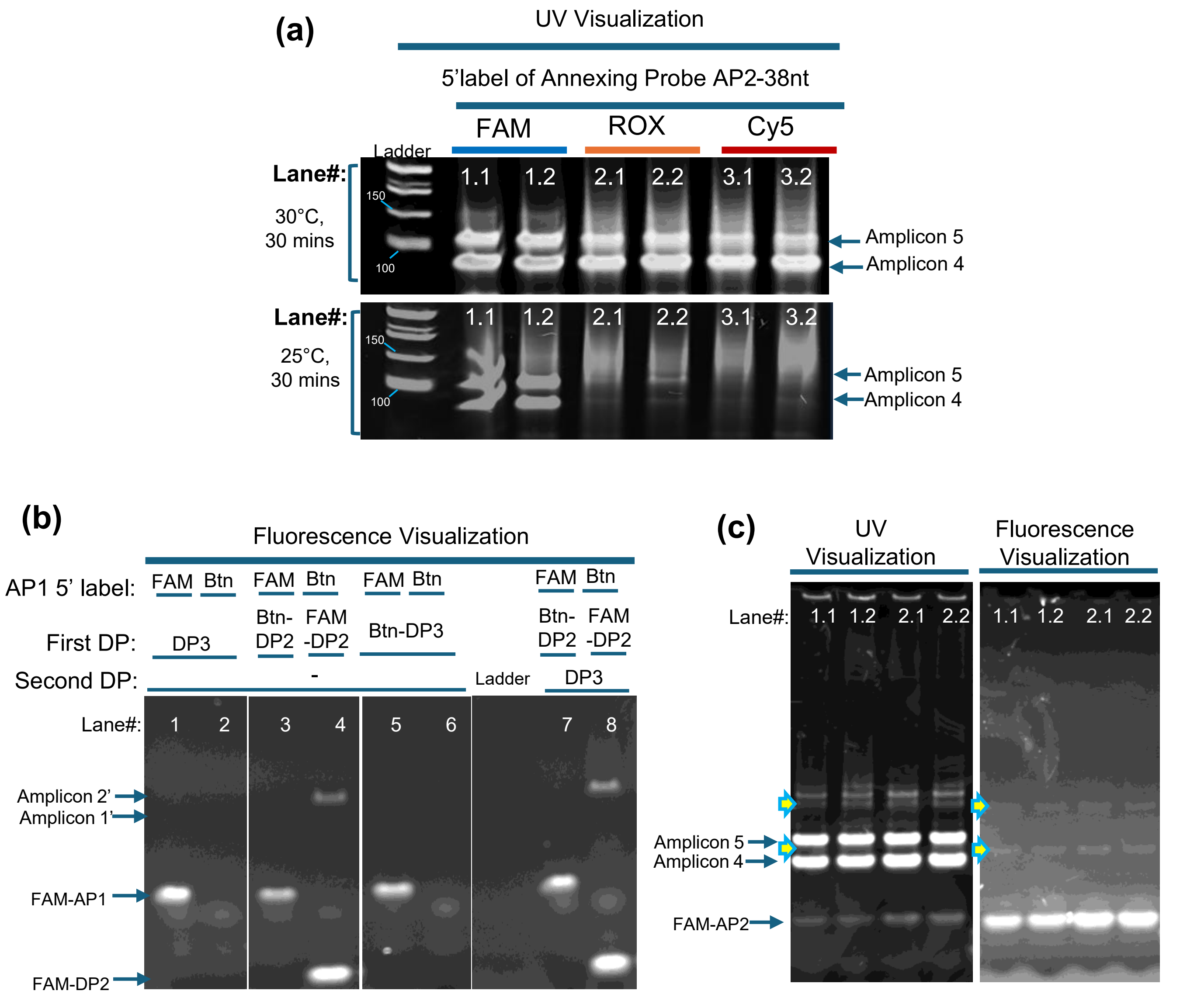

Supplementary Figure 3 | Effect of 5′ Annexing Probe modification on annexation efficiency and probe–amplicon association.

(a-c) show PAGE analysis of ANINA assays using WSSV gDNA.

(a) Effect of differences in aromatic structure and linker composition of 5′ label of Annexing Probe (AP2-38nt) on amplification of PP2-15nt, Btn-DP1-20nt, and DP2-24nt when incubated at 30°C vs 25°C for 30 minutes. 5' labels used were 6-carboxyfluorescein (FAM), Carboxy-X-Rhodamine (ROX), and Cyanine5 (Cy5). Experiments performed in duplicates. A 50 bp DNA ladder was used as the molecular size marker in both PAGE gels, and at least two reference sizes are indicated in each gel panel.

(b) Fluorescence (FAM filter) imaging of reactions using FAM- or biotin-labeled AP1–38 nt with FAM- or biotin-labeled DP2–24 nt (and post-stain UV image in Fig. 3b). AP migrates separately from amplicons under standard conditions.

(c) PAGE under non-standard conditions preserving higher-order structures. UV (left) shows dsDNA amplicons (PP2-15nt, Btn-DP1-20nt and DP2-24nt); fluorescence (right) reveals additional slower-migrating species associated with FAM-labeled AP2-38nt, consistent with probe–amplicon complexes. Lane 2 samples had higher concentration (200 nM) of AP. Experiments done in duplicates.

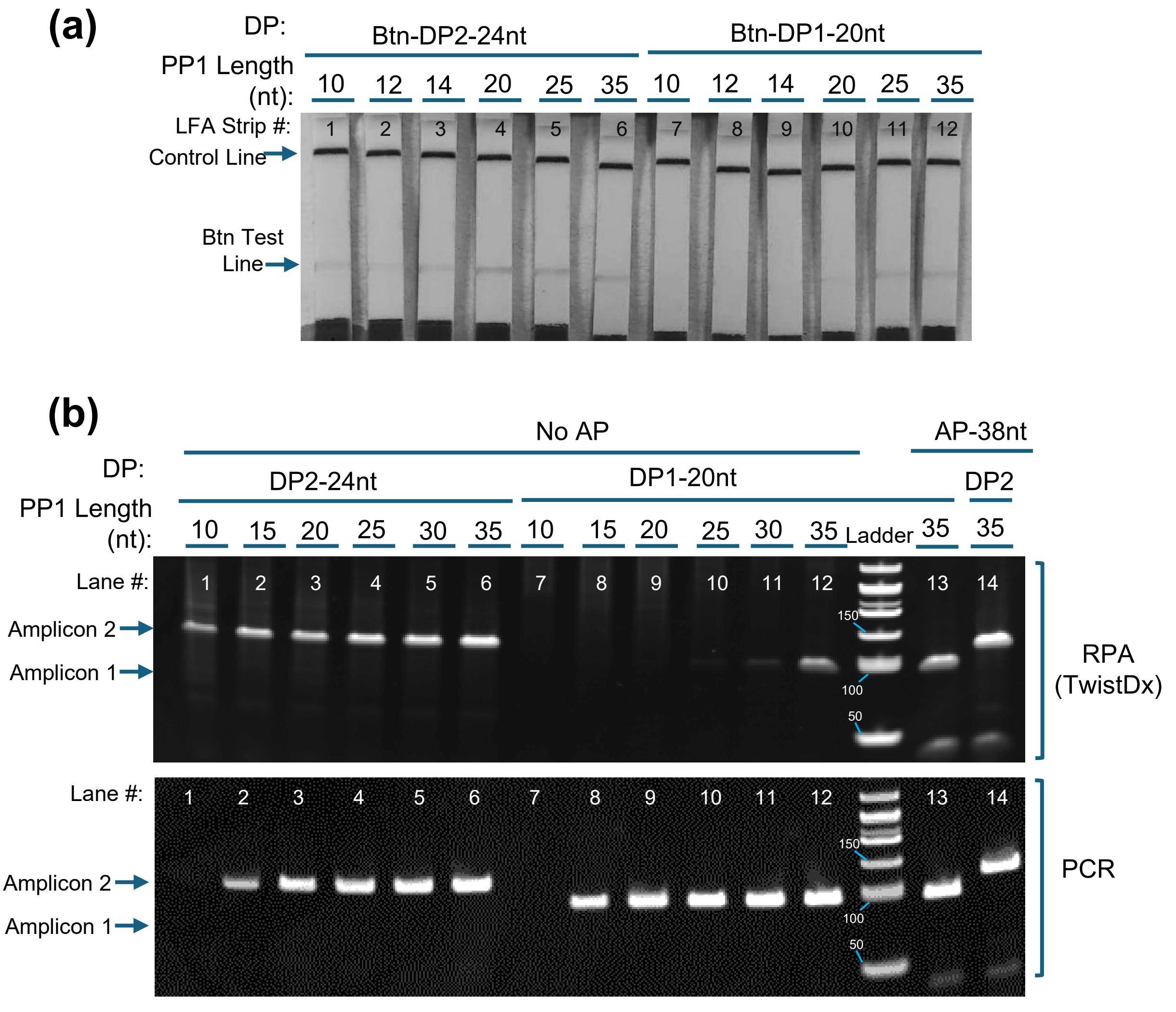

Supplementary Figure 4 | Annexation expands primer design flexibility of recombinase-based amplification and detection.

(a) LFA results using 1T LFA strips corresponding to PAGE results shown in Fig. 4a. Lower signal intensity with shorter primers reflects reduced amplification yield, not loss of specificity.

(b) PAGE results from RPA with TwistDx Basic reaction components (top panel) incubated at 25º C for 30 minutes, and PCR with 40 thermocycles (bottom panel). PP1 of various lengths shown in schematics in Fig. 2a and Fig. 4a were used as Forward Primers while DPs (DP1-20nt and DP2-24nt) were used as Reverse Primers. Lanes 1–12 were performed in the absence of AP, while lanes 13–14 contained AP1-38nt, demonstrating compatibility of AP-containing reactions with the commercial formulation. These lanes are not used to infer AP-dependent rescue of short-primer amplification. Corresponding ANINA reactions are shown in Fig. 2b and Fig. 4b. A 50 bp DNA ladder was used as the molecular size marker in PAGE gels, and at least two reference sizes are indicated in each gel panel.

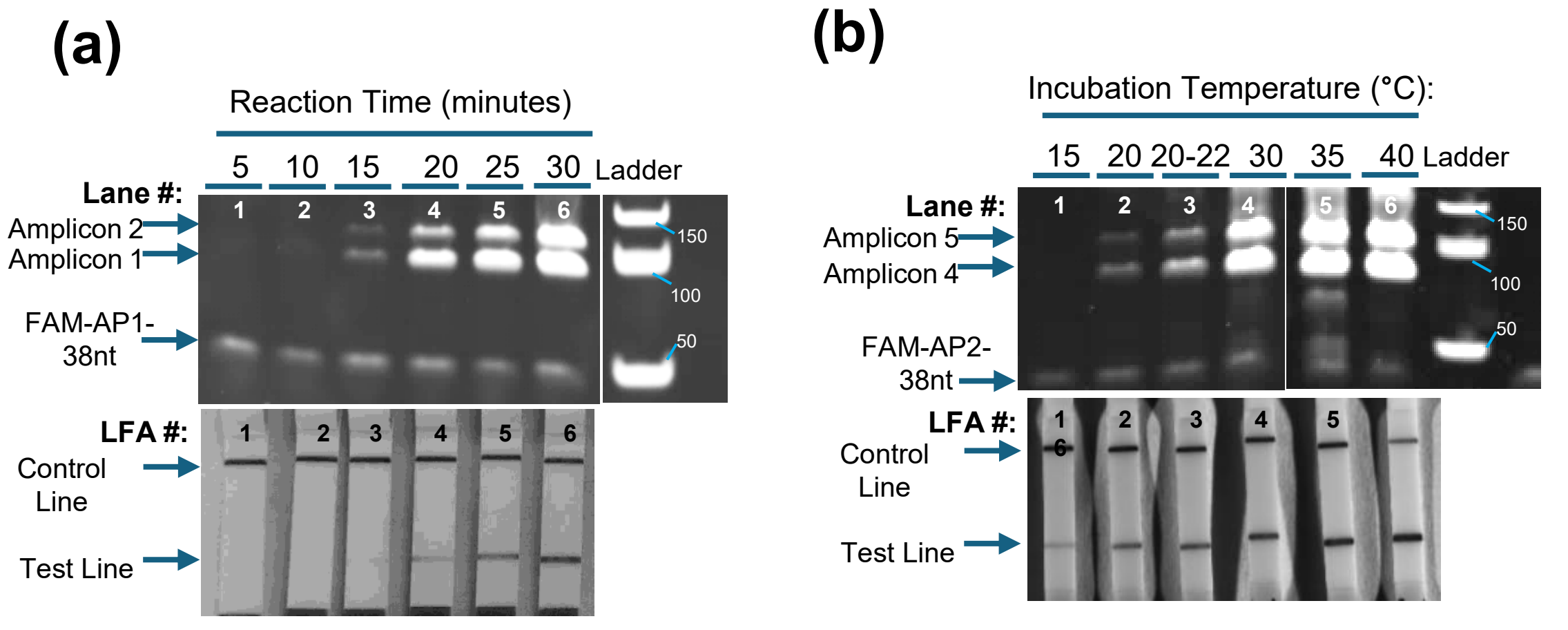

Supplementary Figure 5 | Time and temperature dependence of ANINA amplification and endpoint LFA detection.

PAGE and corresponding LFA detection of ANINA amplification products generated from ~10^6 copies of WSSV gDNA extracted from WSSV-infected shrimp.

(a) Reactions incubated for 30 minutes at 15°C, 20°C, ~20–22°C (benchtop ambient conditions), 30°C, 35°C, and 40°C using FAM-AP1-38 nt, PP1-15 nt, Btn-DP1-20 nt, and DP2-24 nt.

(b) Reactions incubated at 25°C and quenched at defined time points (5, 10, 15, 20, 25, and 30 minutes) using FAM-AP2-38 nt, PP2-15 nt, Btn-DP1-20 nt, and DP2-24 nt. Gel panels were derived from two PAGE gels from the same experiment run under identical conditions.

A 50 bp DNA ladder was used as the molecular size marker in PAGE gels, and at least two reference sizes are indicated in each gel panel. All amplification conditions were evaluated in at least two independent experimental runs with similar results.

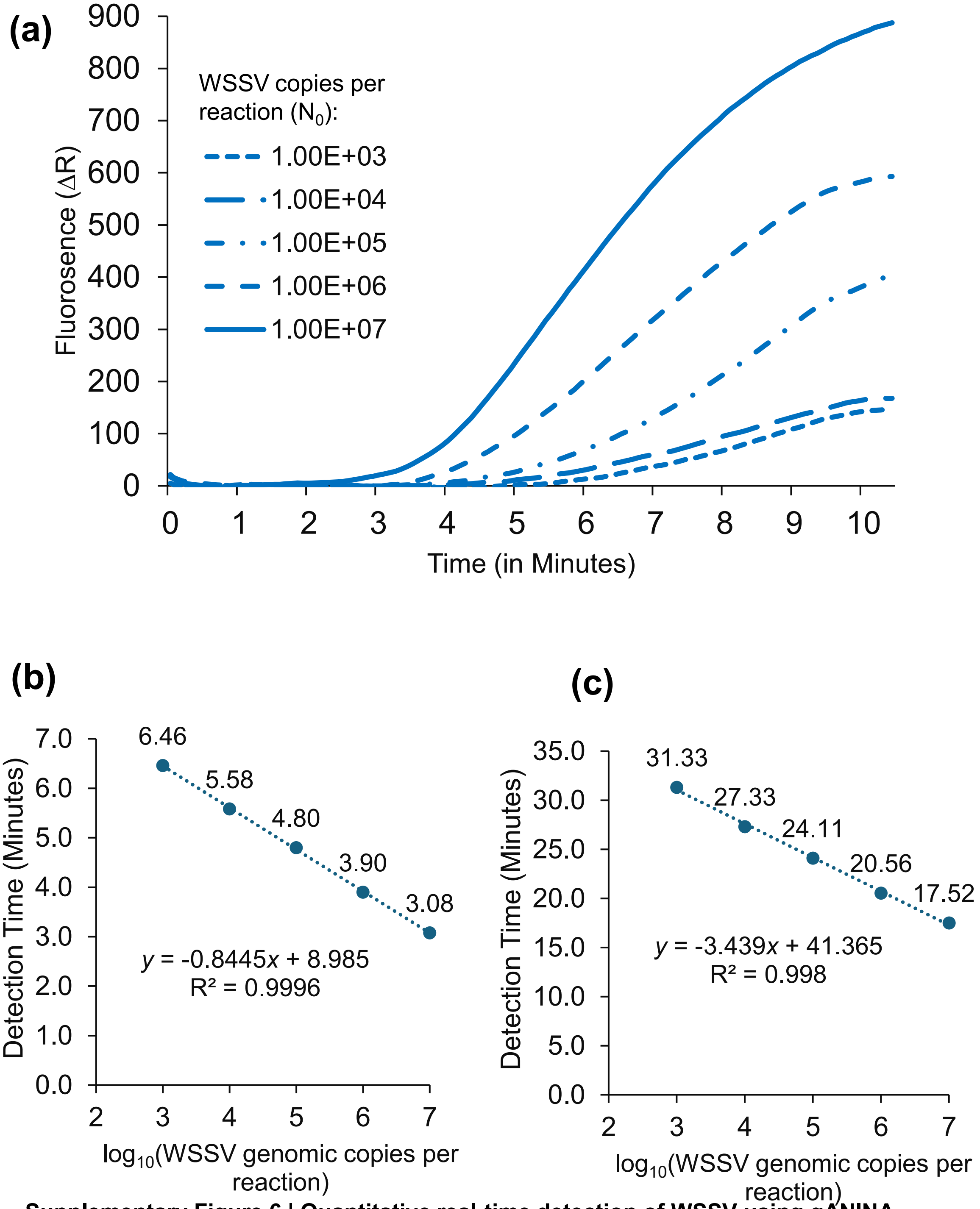

Supplementary Figure 6 | Quantitative real-time detection of WSSV using qANINA compared with qPCR.

(a) Real-time qANINA amplification curves for WSSV genomic standards (10^3–10^6 copies per reaction) and no-template controls (NTC), plotted as normalized fluorescence (ΔR) versus time. Plot is representative of two independent amplification reactions performed for each input concentration. A fixed fluorescence threshold (ΔR = 100) was selected such that NTCs remained below threshold throughout the run.

Detection time (Dt) vs $\log_{10}$(WSSV genomic copies per reaction) of (b) qANINA and (c) qPCR. Plotted Dt was average of two independent amplification replicates. Plots represent least-squares linear regression fits of Dt as a function of $\log_{10}$(WSSV copies per reaction). Regression equations and R^2 values are shown; R^2 indicates goodness of fit. Effective doubling time was calculated from the slope of the Dt versus $\log_{10}$(WSSV copies per reaction) regression as described in the Methods.

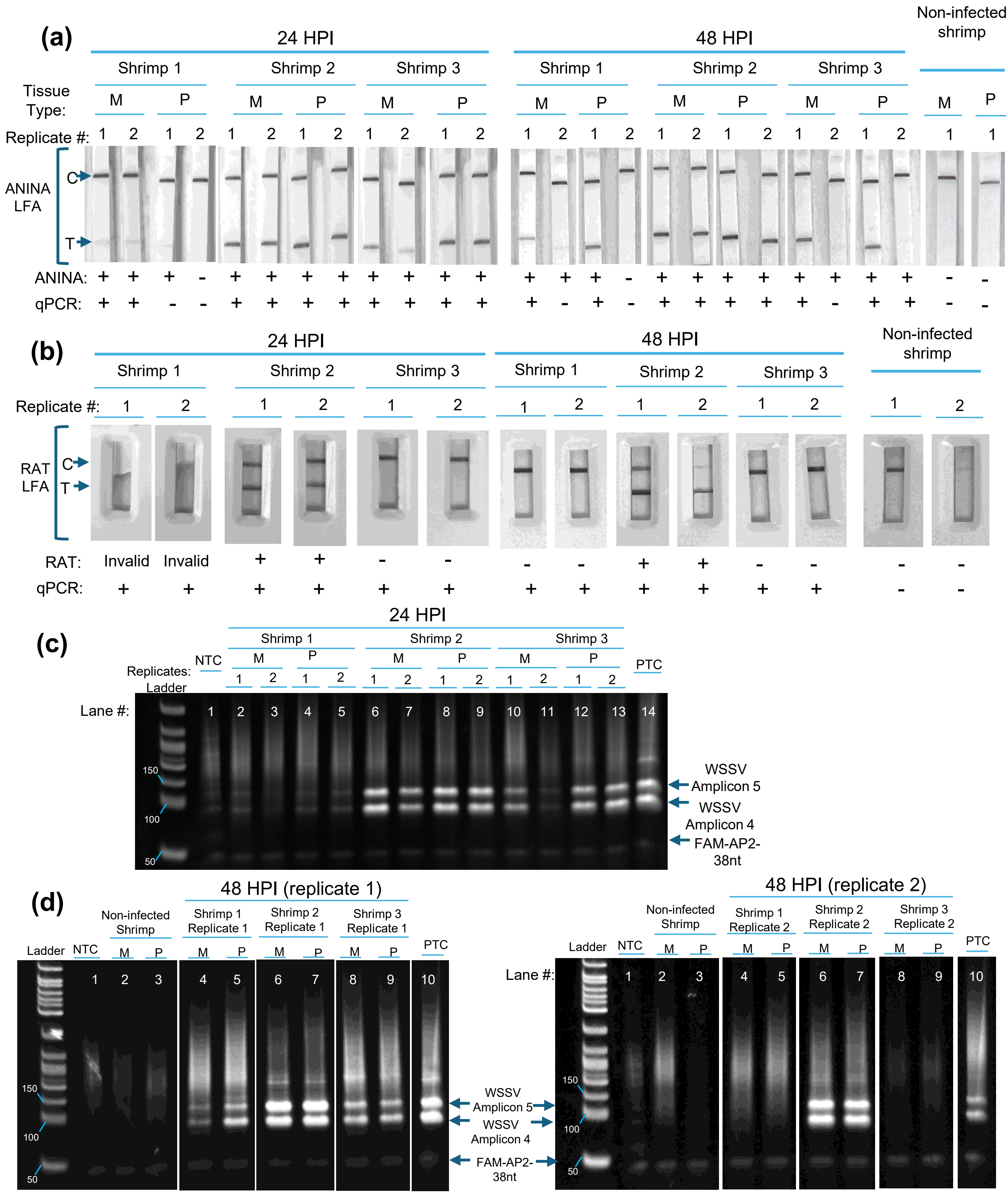

Supplementary Figure 7 | Replicate-level results of instrument-free ANINA detection from WSSV-infected shrimp.

(a-b) LFA images from all replicates for (a) the instrument-free ANINA workflow (four replicate measurements from each infected shrimp, two tissue types x two test replicates), and (b) the corresponding rapid antigen test (RAT) workflow (two test replicates per shrimp) shown in main text Figure 7d. Tissue types are M: Muscle, P: Pleopod. Tables report qualitative LFA interpretation alongside the matched qPCR infection status. qPCR results are shown in Supplementary Table 2.

(c-d) PAGE analysis of amplification products corresponding to samples shown in panel (a). (c) shows PAGE results from 24 hours post infection (HPI) and (d) shows PAGE results from 48 HPI. A 50 bp DNA ladder was used as the molecular size marker in the PAGE gel, and two reference sizes are indicated.

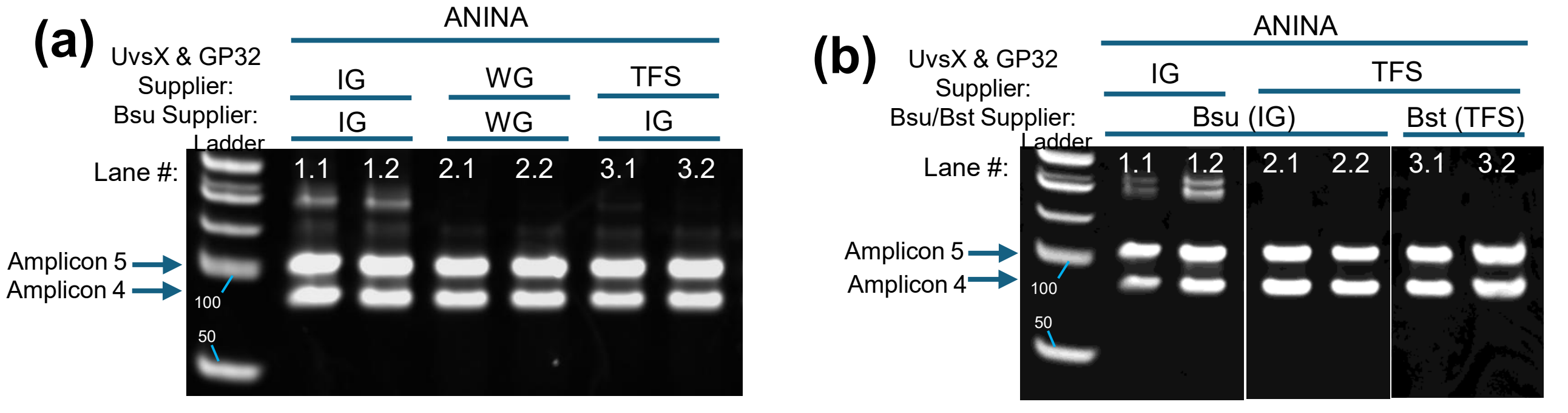

Supplementary Figure 8 | Comparable ANINA amplification across enzyme suppliers.

PAGE analysis of ANINA amplification products generated using FAM-AP2-38nt, PP2-15nt, Btn-DP1-20nt, and DP2-24nt. All reactions were run in duplicates using ~10^6 copies of WSSV gDNA and were incubated at 25°C for 30 minutes. The recombinase-based enzyme mix was prepared using consistent amounts of UvsX, GP32, and DNA polymerase from three different suppliers: Intact Genomics (IG), Watchmaker Genomics (WG), and Thermo Fisher Scientific (TFS), showing consistent amplification unaffected by enzyme supplier change. (a) Since TFS doesn't supply Bsu, IG Bsu was used with TFS UvsX and GP32. (b) Replacement of Bsu DNA polymerase with Bst did not affect target amplicon yield. A 50 bp DNA ladder was used as the molecular size marker in the PAGE gel, and two reference sizes are indicated.

Supplementary Table 1: List of expected ANINA amplicon sequences.

**Genomic Target**	**Amplicon Number and length**	**Sequence**	**GC %**	$T_{M,}$ (in °C)	**ΔG (kcal/mol)**	**2°** T_M (in °C)
WSSV *VP19* (DNA)	WSSV Amplicon 1 (97 bp)	TGGCTCGCATGGGTCTCTTTTTGATCGTTGCTATC<br>TCAATTGGTATCCTCGTCCTGGCCGTCATGAATG<br>TATGGATGGGACCAAAGAAGGACAGCGA	49.5	81.2	-7.9	38.1
	WSSV Amplicon 2 (123 bp)	TGGCTCGCATGGGTCTCTTTTTGATCGTTGCTATC<br>TCAATTGGTATCCTCGTCCTGGCCGTCATGAATG<br>TATGGATGGGACCAAAGAAGGACAGCGATTCTGA<br>CACTGATAAGGACACCGATG	48.8	80.5	-11.6	43.6
	WSSV Amplicon 3 (179 bp)	TGGCTCGCATGGGTCTCTTTTTGATCGTTGCTATC<br>TCAATTGGTATCCTCGTCCTGGCCGTCATGAATG<br>TATGGATGGGACCAAAGAAGGACAGCGATTCTGA<br>CACTGATAAGGACACCGATGATGATGACGACACT<br>GCCAACGATAACGATGATGAGGACAAATATAAGA<br>ACAGGACC	46.9	79.4	-17.6	44.5
	WSSV Amplicon 4 (81 bp)	CTTTTTGATCGTTGCTATCTCAATTGGTATCCTCG<br>TCCTGGCCGTCATGAATGTATGGATGGGACCAAA<br>GAAGGACAGCGA	46.9	80.2	-6.0	40.2
	WSSV Amplicon 5 (107 bp)	CTTTTTGATCGTTGCTATCTCAATTGGTATCCTCG<br>TCCTGGCCGTCATGAATGTATGGATGGGACCAAA<br>GAAGGACAGCGATTCTGACACTGATAAGGACACC<br>GATG	46.7	80.1	-10.3	43.4
	WSSV Amplicon 6 (163 bp)	CTTTTTGATCGTTGCTATCTCAATTGGTATCCTCG<br>TCCTGGCCGTCATGAATGTATGGATGGGACCAAA<br>GAAGGACAGCGATTCTGACACTGATAAGGACACC<br>GATGATGATGACGACACTGCCAACGATAACGATG<br>ATGAGGACAAATATAAGAACAGGACC	45.4	79.8	-16.2	44.4
EBV *BHRF1* (DNA)	EBV Amplicon 1 (107 bp)	CTTGCTGGACTGACTTTGAGTCTGTTAGTTATATG<br>TAGTTATTTATTTATCTCCAGAGGAAGACACTAAT<br>CTATACATTTTCTCAGCACTTTATATGAATCAGGG<br>TC	33.9	79.2	-5.7	40.8
	EBV Amplicon 2 (159 bp)	CTTGCTGGACTGACTTTGAGTCTGTTAGTTATATG<br>TAGTTATTTATTTATCTCCAGAGGAAGACACTAAT<br>CTATACATTTTCTCAGCACTTTATATGAATCAGGG<br>TCATTGGGCCTGCGGGGAACTGAGCCAGTAGGA<br>TATTAGGCAAGGGTGACACAG	41.0	80.4	-13.5	47.1
Ng TIGR00645 (DNA)	Ng Amplicon (118 bp)	CGATTTGTGCCTATAAGTTTTTGAAATCGTTGAAG<br>CATCTGGTCATGAATTTGGATGTGTCGGACGAAA<br>ACGCCATCATGCTCGCTGTTTTAAATCTGATTGAT<br>GTGGTTATGATTGC	39.8	81.0	-10.7	44.8
RSV-Long *M* gene (ssRNA)	RSV Amplicon (207 bp)	AAGGAGCATTCAAATACATAAAGCCGCAAAGTCA<br>ATTCATAGTAGATCTTGGAGCTTACCTAGAAAAAG<br>AAAGTATATATTATGTTACCACAAATTGGAAGCAC<br>ACAGCTACACGATTTGCAATCAAACCCATGGAAG<br>ATTAACCTTTTTCCTCCACATCAGTGAGTCAATTC<br>ATACAAACTTTCTACCTACATTCTTCACTTCACC	36.2	82.4	-16.0	41.7

Supplementary Table 2: qPCR assay results and Diagnostic performance evaluation of the WSSV-shrimp infection studies (Fig. 7 and Supplementary Fig. 7).
Pos: Positive for WSSV infection, Neg: Negative for infection.

Time-points	Shrimp #	Tissue Type	Extraction replicate #	qPCR Ct	WSSV copies/ µL	qPCR call (each replicate)	ANINA LFA call (each replicate)	Average copy numbers	qPCR call (each shrimp)[1]	ANINA call (each shrimp)[1]	RAT call (each shrimp)[1]
24 hours	1	Muscle	1	38.25	2.1E+02	Pos	Pos	4.4E+02	Pos	Pos	Invalid
			2	36.38	6.8E+02	Pos	Pos				
		Pleopod	1	No Ct	0.0E+00	Neg	Pos				
			2	No Ct	0.0E+00	Neg	Neg				
	2	Muscle	1	31.63	6.8E+03	Pos	Pos	3.4E+04	Pos	Pos	Pos
			2	30.06	1.9E+04	Pos	Pos				
		Pleopod	1	28.40	5.1E+04	Pos	Pos				
			2	28.26	5.8E+04	Pos	Pos				
	3	Muscle	1	36.62	3.1E+02	Pos	Pos	6.2E+03	Pos	Pos	Neg
			2	33.31	2.3E+03	Pos	Pos				
		Pleopod	1	30.69	1.3E+04	Pos	Pos				
			2	31.20	8.7E+03	Pos	Pos				
48 hours	1	Muscle	1	36.37	1.9E+01	Pos	Pos	5.0E+01	Pos	Pos	Neg
			2	No Ct	0.0E+00	Neg	Pos				
		Pleopod	1	35.92	8.0E+01	Pos	Pos				
			2	No Ct	0.0E+00	Neg	Neg				
	2	Muscle	1	21.82	3.4E+05	Pos	Pos	4.6E+06	Pos	Pos	Pos
			2	20.65	7.6E+05	Pos	Pos				
		Pleopod	1	17.29	7.3E+06	Pos	Pos				
			2	16.80	1.0E+07	Pos	Pos				
	3	Muscle	1	31.87	4.0E+02	Pos	Pos	2.7E+02	Pos	Pos	Neg
			2	No Ct	0.0E+00	Neg	Pos				
		Pleopod	1	32.33	3.0E+02	Pos	Pos				
			2	35.88	1.3E+02	Pos	Pos				
Non-infected	1	Muscle	1	No Ct	0.0E+00	Neg	Neg	0.0E+00	Neg	Neg	Neg
		Pleopod	1	No Ct	0.0E+00	Neg	Neg				
Template controls	PTC	N/A	N/A	17.22	8.0E+06	Pos	Pos	8.0E+06	Pos	Pos	N/A
	NTC	N/A	N/A	No Ct	0.0E+00	Neg	Neg	0.0E+00	Neg	Neg	N/A

[1] For qPCR and ANINA, four replicate measurements were available per shrimp (two tissues × two extraction replicates), whereas two replicate measurements were available per shrimp for the rapid antigen test (RAT). A shrimp was classified as positive if ≥50% of replicate measurements were positive (≥2 of 4 for qPCR/ANINA; ≥1 of 2 for RAT). A shrimp was classified as negative if <50% of replicate measurements were positive.

Diagnostic Sensitivity (Dse) = #(True Pos)/(#(True Pos) + #(False Neg))

Diagnostic Specificity (Dsp) = #(True Neg)/(#(True Neg) + #(False Pos))

		qPCR (Lab reference assay)					qPCR (Lab reference assay)		
		Pos	Neg	Total			Pos	Neg	Total
ANINA WSSV Integrated Assay	Pos	6	0	6	Innocreate WSSV RAT	Pos	2	0	2
	Neg	0	1	1		Neg	3	1	4
	Total	6	1	7		Total	5	1	6
	Relative Dx Sensitivity (Dse)			100.0%		Relative Dx Sensitivity (Dse)			40.0%
	Relative Dx Specificity (Dsp)			100.0%		Relative Dx Specificity (Dsp)			100.0%

Supplementary Table 3: *In-silico* cross-reactivity analysis of the WSSV *VP19* ANINA oligonucleotide set against WSSV, host (shrimp) and non-WSSV representative pathogens of common shrimp infectious diseases.

White Spot Syndrome Virus (WSSV; taxid: 342409)					
NCBI Accession: NC_003225.3					
Oligonucleotide	Max Score	Total Score	E-Value	Percent Identity	Query Cover
PP2-15nt	30.2	1506	3.50E-02	100%	100%
DP1-20nt	40.1	1023	1.00E-04	100%	100%
DP2-24nt	48.1	821	7.00E-07	100%	100%
AP2-38nt	71.3	71.3	5.00E-14	100%	100%

Pacific White Shrimp (*Litopenaeus vannamei*; taxid:6689)					
NCBI Accession: GCF_042767895.1					
Oligonucleotide	Max Score	Total Score	E-Value	Percent Identity	Query Cover
PP2-15nt	28.2	28.2	0.97	100%	93%
DP1-20nt	30.2	50.5	0.62	100%	75%
DP2-24nt	30.2	30.2	1.1	100%	63%
AP2-38nt	No similarity found				

Penaeid Shrimp Infectious Myonecrosis Virus (IMNV; taxid: 3426466)					
NCBI Accession: NC_007915.3					
Oligonucleotide	Max Score	Total Score	E-Value	Percent Identity	Query Cover
PP2-15nt	20.3	192	0.54	100%	100%
DP1-20nt	18.3	313	4.2	100%	95%
DP2-24nt	18.3	198	5.9	100%	92%
AP2-38nt	No similarity found				

Enterocytozoon hepatopenaei (EHP; taxid: 646526)					
NCBI Accession:					
Oligonucleotide	Max Score	Total Score	E-Value	Percent Identity	Query Cover
PP2-15nt	20.3	51	0.55	100%	67%
DP1-20nt	22.3	96.1	0.27	100%	70%
DP2-24nt	20.3	65.4	1.5	100%	83%
AP2-38nt	No similarity found				

Acute Hepatopancreatic Necrosis Disease (AHPND; *Vibrio parahaemolyticus*; taxid: 670)					
NCBI Accession: NZ_AP014859.1					
Oligonucleotide	Max Score	Total Score	E-Value	Percent Identity	Query Cover
PP2-15nt	28.2	489	8	100%	100%
DP1-20nt	30.2	285	4.1	100%	100%
DP2-24nt	28.2	103	28	100%	100%
AP2-38nt	*No similarity found*				

Necrotising Hepatopancreatitis (NHP; *Hepatobacter penaei*, taxid: 1274402)					
NCBI Accession: NZ_JQAJ00000000					
Oligonucleotide	Max Score	Total Score	E-Value	Percent Identity	Query Cover
PP2-15nt	18.3	32.7	0.62	100%	60%
DP1-20nt	18.3	106	0.97	100%	90%
DP2-24nt	18.3	47.1	1.3	100%	46%
AP2-38nt	No similarity found				

Decapod Iridescent Virus / Shrimp Hemocyte Iridescent Virus (DIV1/SHIV; taxid: 3428192)					
NCBI Accession: NC_055165.1					
Oligonucleotide	Max Score	Total Score	E-Value	Percent Identity	Query Cover
PP2-15nt	24.3	6344	0.12	100%	100%
DP1-20nt	16.4	118	37	100%	92%
DP2-24nt	18.3	153	14	100%	96%
AP2-38nt	No similarity found				

Taura Syndrome Virus (TSV; taxid: 3048357)					
NCBI Accession: NC_003005.1					
Oligonucleotide	Max Score	Total Score	E-Value	Percent Identity	Query Cover
PP2-15nt	16.4	16.4	17	100%	53%
DP1-20nt	16.4	145	31	100%	100%
DP2-24nt	18.3	94.1	11	100%	83%
AP2-38nt	No similarity found				

Infectious Hypodermal and Hematopoietic Necrosis Virus (IHHNV; taxid: 3052615)					
NCBI Accession: NC_002190.2					
Oligonucleotide	Max Score	Total Score	E-Value	Percent Identity	Query Cover
PP2-15nt	18.3	47.1	5.2	100%	100%
DP1-20nt	16.4	118	37	91.67%	100%
DP2-24nt	18.3	153	14	100%	96%
AP2-38nt	No similarity found				

Yellowhead Disease Virus (YHV; taxid: 3048455)					
NCBI Accession: NC_043505.1					
Oligonucleotide	Max Score	Total Score	E-Value	Percent Identity	Query Cover
PP2-15nt	18.3	440	3	100%	100%
DP1-20nt	22.3	563	0.38	100%	100%
DP2-24nt	20.3	596	2	100%	100%
AP2-38nt	No similarity found				

Supplementary Table 4: Reagent Sources used in ANINA reactions.

**Reagent name**	**Supplier**	**Catalog number**
T4 UvsX Recombinase	Intact Genomics	3567, 3567GF
	Watchmaker Genomics	7K0124
	ThermoFisher Scientific	A72124
T4 GP32 Protein	Intact Genomics	3518, 3519GF
	Watchmaker Genomics	7K0127
	ThermoFisher Scientific	A72123
Bsu DNA Polymerase, Large Fragment	Intact Genomics	3587, 3587GF
	Watchmaker Genomics	7K0128
T4 UvsY Protein (optional)	Intact Genomics	3577, 3577GF
	Watchmaker Genomics	7K0126
	ThermoFisher Scientific	A72125
Bst DNA Polymerase, Large Fragment	ThermoFisher Scientific	A56657
SuperScript IV Reverse Transcriptase	ThermoFisher Scientific	18090200
RNaseH	ThermoFisher Scientific	EN0202
Creatine Kinase	Millipore Sigma	C3755
Phosphocreatine	Millipore Sigma	CRPHO-RO/10621714001
Poly-ethylene glycol	Millipore Sigma	94646
Trehalose	Millipore Sigma	T5251
DTT	Millipore Sigma	43815
dNTPs	New England Biolabs	N0447L
ATP	ThermoFisher Scientific	R0441
