# Supplementary material for: Annexing isothermal nucleotide amplification enables probe-guided amplification and direct detection at ambient temperature
Source: Nat Commun. 2026 Jul 14;17:8638. doi: 10.1038/s41467-026-75361-6 (PMC13486510; doi:10.1038/s41467-026-75361-6)
Supplement: Supplementary file 2 [file 41467_2026_75361_MOESM2_ESM.pdf]

**Description of Additional Supplementary Files**

File Name: Supplementary Data 1
Description: WSSV VP19 ANINA oligonucleotide sequences and corresponding modification chemistries. (Table 1)

File Name: Supplementary Data 2
Description: ANINA and qPCR oligonucleotide sequences and corresponding modification chemistries for EBV, N. gonorrhoeae, RSV, and WSSV (only qPCR) targets. (Table 2)

File Name: Supplementary Data 3
Description: Comparison of ANINA with representative recombinase-based isothermal systems. (Table 3)

File Name: Supplementary Data 4
Description: List of expected ANINA amplicon sequences.

File Name: Supplementary Data 5
Description: qPCR assay results and Diagnostic performance evaluation of the WSSV-shrimp infection studies (Fig. 7 and Supplementary Fig. 7).

File Name: Supplementary Data 6
Description: In-silico cross-reactivity analysis of the WSSV VP19 ANINA oligonucleotide set against WSSV, host (shrimp) and non-WSSV representative pathogens of common shrimp infectious diseases.

File Name: Supplementary Data 7
Description: Reagent Sources used in ANINA reactions.
