# Supplementary material for: Annexing isothermal nucleotide amplification enables probe-guided amplification and direct detection at ambient temperature
Source: Nat Commun. 2026 Jul 14;17:8638. doi: 10.1038/s41467-026-75361-6 (PMC13486510; doi:10.1038/s41467-026-75361-6)
Supplement: Supplementary file 3 [file 41467_2026_75361_MOESM4_ESM.pdf]

nature portfolio

Corresponding author(s): Anindita Roy

Last updated by author(s): 6/22/2026

Reporting Summary

Nature Portfolio wishes to improve the reproducibility of the work that we publish. This form provides structure for consistency and transparency in reporting. For further information on Nature Portfolio policies, see our Editorial Policies and the Editorial Policy Checklist.

Statistics

For all statistical analyses, confirm that the following items are present in the figure legend, table legend, main text, or Methods section.

n/a	Confirmed	
☐	☒	The exact sample size (*n*) for each experimental group/condition, given as a discrete number and unit of measurement
☐	☒	A statement on whether measurements were taken from distinct samples or whether the same sample was measured repeatedly
☒	☐	The statistical test(s) used AND whether they are one- or two-sided<br>*Only common tests should be described solely by name; describe more complex techniques in the Methods section.*
☒	☐	A description of all covariates tested
☐	☒	A description of any assumptions or corrections, such as tests of normality and adjustment for multiple comparisons
☐	☒	A full description of the statistical parameters including central tendency (e.g. means) or other basic estimates (e.g. regression coefficient) AND variation (e.g. standard deviation) or associated estimates of uncertainty (e.g. confidence intervals)
☒	☐	For null hypothesis testing, the test statistic (e.g. *F*, *t*, *r*) with confidence intervals, effect sizes, degrees of freedom and *P* value noted<br>*Give P values as exact values whenever suitable.*
☒	☐	For Bayesian analysis, information on the choice of priors and Markov chain Monte Carlo settings
☒	☐	For hierarchical and complex designs, identification of the appropriate level for tests and full reporting of outcomes
☒	☐	Estimates of effect sizes (e.g. Cohen's *d*, Pearson's *r*), indicating how they were calculated

Our web collection on statistics for biologists contains articles on many of the points above.

Software and code

Policy information about availability of computer code

Data collection	Agilent AriaMx software v2.1 (Agilent Technologies) was used for real-time fluorescence acquisition and Ct determination for qANINA and qPCR experiments. Gel and lateral flow assay (LFA) images were acquired using VisionWorks software on a UVP GelStudio system (Analytik Jena).
Data analysis	Agilent AriaMx software v2.1 was used to analyze real-time fluorescence amplification assays (qPCR and qANINA) and determine Ct values. VisionWorks LS Analysis Software (v11.2.22322.8724, Analytik Jena) was used for analyzing gel and LFA images and for densitometric quantification of lateral flow test line intensity. Standard curve fitting and regression-based calculations (including doubling time from Dt vs log10 input) were performed using Microsoft Excel. All graphical analysis were carried out on Microsoft Excel or GraphPad Prism 11.0.1.

For manuscripts utilizing custom algorithms or software that are central to the research but not yet described in published literature, software must be made available to editors and reviewers. We strongly encourage code deposition in a community repository (e.g. GitHub). See the Nature Portfolio guidelines for submitting code & software for further information.

Data

Policy information about availability of data

All manuscripts must include a data availability statement. This statement should provide the following information, where applicable:

- Accession codes, unique identifiers, or web links for publicly available datasets
- A description of any restrictions on data availability
- For clinical datasets or third party data, please ensure that the statement adheres to our policy

All data supporting the findings of this study are available within the Article, Supplementary Information, and Source Data file. The Excel version of the Tables 1-3 and Supplementary Tables 1-4 are also available in the Supplementary Data file. The Raw data underlying all figures and tables are provided in the Source Data file accompanying this paper and include raw quantitative outputs, uncropped gel and lateral flow assay images, qPCR and qANINA fluorescence measurements, and associated analyses. Source data are provided with this paper.
Lyophilized Seek Amplification™ beads and other proprietary materials described in this study are available from the corresponding author through Seek Labs Inc. upon reasonable request, subject to applicable material transfer agreements and intellectual property considerations.

Research involving human participants, their data, or biological material

Policy information about studies with human participants or human data. See also policy information about sex, gender (identity/presentation), and sexual orientation and race, ethnicity and racism.

Reporting on sex and gender	Commercially obtained, de-identified pooled human saliva (Precision Biospecimen Solutions, Inc.; and Innovative Research, Inc.) was used as a background matrix. No donor metadata (including sex or gender) were recorded.
Reporting on race, ethnicity, or other socially relevant groupings	Race, ethnicity, and other demographic metadata were not available or recorded. Saliva was used only as a background matrix for contrived samples.
Population characteristics	No individual-level demographic data were available.
Recruitment	De-identified saliva was purchased from a commercial supplier (Precision Biospecimen Solutions, Inc.; and Innovative Research, Inc.). No recruitment was carried out for this research.
Ethics oversight	De-identified human saliva samples used as background matrix for virus-spiked experiments were obtained from commercial vendors (Precision Biospecimen Solutions, Inc. and Innovative Research, Inc.). Samples were originally collected under vendor IRB-approved protocols with informed consent or IRB-approved waivers permitting secondary research use. Race, ethnicity, and other demographic metadata were not available or recorded. Because all samples were de-identified and no identifiable private information was accessible to the investigators, this work did not constitute human subjects research. All specimens were handled in accordance with applicable biosafety requirements and Seek Labs' Quality Management System and Operational Safety Plan.

Note that full information on the approval of the study protocol must also be provided in the manuscript.

Field-specific reporting

Please select the one below that is the best fit for your research. If you are not sure, read the appropriate sections before making your selection.

☒ Life sciences ☐ Behavioural & social sciences ☐ Ecological, evolutionary & environmental sciences

For a reference copy of the document with all sections, see nature.com/documents/nr-reporting-summary-flat.pdf

Life sciences study design

All studies must disclose on these points even when the disclosure is negative.

Sample size	No statistical methods were used to pre-determine sample size. Sample sizes and replicate numbers were chosen based on standard assay-development practice and are reported in the figure legends and Methods. Key findings were reproduced across independent experiments as indicated.
Data exclusions	No data were excluded unless explicitly stated. For slope and doubling-time analyses, the single-copy EBV standard was excluded because it was detected by qANINA but not by qPCR. One 10-copy EBV data point was excluded from qANINA analysis as an outlier, and two matched EBV standard data points were excluded from qPCR analysis due to lack of detection. These are noted in figures and methods..
Replication	Experiments were repeated in independent runs as indicated in figure legends. Representative gel and lateral flow images are shown; replicate experiments yielded consistent qualitative results.
Randomization	Randomization was not used because samples were assigned to experimental conditions by design (e.g., defined primer/probe variants, defined spike-in concentrations).

Blinding	For shrimp infection studies, lateral flow/antigen test results were interpreted qualitatively without prior knowledge of qPCR results. Blinding was not used for other assay-development experiments.

Reporting for specific materials, systems and methods

We require information from authors about some types of materials, experimental systems and methods used in many studies. Here, indicate whether each material, system or method listed is relevant to your study. If you are not sure if a list item applies to your research, read the appropriate section before selecting a response.

Materials & experimental systems

n/a	Involved in the study
☐	☒ Antibodies
☒	☐ Eukaryotic cell lines
☒	☐ Palaeontology and archaeology
☐	☒ Animals and other organisms
☒	☐ Clinical data
☒	☐ Dual use research of concern
☒	☐ Plants

Methods

n/a	Involved in the study
☒	☐ ChIP-seq
☒	☐ Flow cytometry
☒	☐ MRI-based neuroimaging

Antibodies

Antibodies used	No antibodies were directly used as experimental reagents in this study. Endpoint detection was performed using commercially manufactured lateral flow devices (Milenia Biotec HybriDetect 1T and 2T), which contain proprietary anti-tag capture antibodies and gold nanoparticle conjugates integrated into the test strips by the manufacturer. A commercial WSSV rapid antigen test (Innocreate WSSV RP) was used as a comparator assay. Supplier and catalog information for all commercial lateral flow devices and antigen tests are provided in the Methods. As no antibodies were independently sourced, prepared, or experimentally optimized by the authors, antibody-specific details such as clone names, lot numbers, concentrations, or dilutions are not applicable.
Validation	No primary antibodies were directly used as experimental reagents in this study; therefore, antibody validation reporting for primary antibodies is not applicable. Antibody-containing lateral flow devices were used only as commercially manufactured diagnostic strips according to the manufacturers' instructions. The presence of the control line on each strip served as the manufacturer-built procedural control for valid test performance. No additional in-house antibody validation was performed.

Animals and other research organisms

Policy information about studies involving animals; ARRIVE guidelines recommended for reporting animal research, and Sex and Gender in Research

Laboratory animals	Specific pathogen-free (SPF) Pacific whiteleg shrimp (Litopenaeus vannamei; juvenile, 1-5 g) were purchased from authorized vendors (Oceanic Institute of Hawaii Pacific University and Homegrown Shrimp USA) and shipped to Seek Labs. Shrimp were maintained in 20–40-gallon saltwater tanks under controlled laboratory conditions. Shrimp importation and research use were authorized under a Utah Division of Wildlife Resources Certificate of Registration (COR No. 4POSS10845; effective 02/04/2022–02/03/2027). Husbandry and biosafety practices followed Utah state regulations, guidance from the USDA-accredited Aquatic Animal Health Laboratory (Florida Atlantic University), published literature, and procedures reviewed by Seek Labs' internal Quality and Safety Committee. Shrimp are invertebrates and are not regulated under the U.S. Animal Welfare Act; therefore, Institutional Animal Care and Use Committee (IACUC) approval was not required.
Wild animals	No wild animals were used.
Reporting on sex	Shrimp sex was not determined and was not considered a biological variable.
Field-collected samples	Samples were not collected from the field.
Ethics oversight	Shrimp importation and research use were authorized under a Utah Division of Wildlife Resources Certificate of Registration (COR No. 4POSS10845; effective 02/04/2022–02/03/2027). Husbandry and biosafety practices followed Utah state regulations, guidance from the USDA-accredited Aquatic Animal Health Laboratory (Florida Atlantic University), published literature45-49, and procedures reviewed by Seek Labs' internal Quality and Safety Committee. Shrimp are invertebrates and are not regulated under the U.S. Animal Welfare Act; therefore, Institutional Animal Care and Use Committee (IACUC) approval was not required. Procedures for handling and disposal of WSSV-infected materials were reviewed by the Utah Department of Natural Resources Division of Wildlife Resources (DWR Fisheries Program). Shrimp sex was not determined and was not considered a biological variable.

Note that full information on the approval of the study protocol must also be provided in the manuscript.

Plants

Seed stocks	N/A
Novel plant genotypes	N/A
Authentication	N/A
