# Supplementary material for: Annexing isothermal nucleotide amplification enables probe-guided amplification and direct detection at ambient temperature
Source: Nat Commun. 2026 Jul 14;17:8638. doi: 10.1038/s41467-026-75361-6 (PMC13486510; doi:10.1038/s41467-026-75361-6)
Supplement: Supplementary file 4 [file 41467_2026_75361_MOESM5_ESM.pdf]

nature portfolio

Peer Review File

Annexing Isothermal Nucleotide Amplification Enables Probe-guided Amplification and Direct Detection at Ambient Temperature

Corresponding Author: Dr Anindita Roy

This file contains all reviewer reports in order by version, followed by all author rebuttals in order by version.

Version 0:

Reviewer comments:

Reviewer #1

(Remarks to the Author)
• The ANINA method implemented during this study provides data to believe the promising potential for use of the technique for early detection of widespread infections and the possibility of point-of-care utility. This will help the industry.
• The data proves the concept that in instrument-free mode, the field level detection will be possible while the samples are treated at room temperature.
• The methodology and results are well explained.
• Since such methodologies are closely linked to widespread use in the trade, therefore, it needs to be proven on clinical samples in field conditions and generate robust data. This will add value to the work and will be of direct use. Such clinical samples can be corroborated with real-time PCR and provide a good comparative view on the sensitivity of the test, meaning how many viral copies the method is able to detect. Sensitivity will be the most important factor for the industry to believe in the application of the results, which otherwise show a great promise.
• Based on the actual field trials, the possible limitations of the test can also be defined.
• The manuscript should include a brief discussion on the risk of non-specific amplification, which is inherent to isothermal amplification. Furthermore, carryover contamination risks associated with recombinase-based amplification should be addressed.
• A critical comparison of ANINA with nfo-RPA, SHERLOCK, and related technologies should be included. This comparison should include potential limitations of the method, including probe design constraints, multiplexing capability, and operational challenges.
• The Specificity of the assay should be further evaluated against closely related pathogens (eg, for WSSV, closely related shrimp viruses) to eliminate cross-reactivity.
• To improve clarity and impact, it is recommended to include a comparative table summarizing key diagnostic parameters (LOD, assay time, equipment required, estimated cost per test) along with advantages and limitations in comparison with established methods.
• Statistical analysis, like Probit regression for determining LoD and Diagnostic performance evaluation (true positive, true negative, false positive, and false negative), diagnostic sensitivity, and specificity using a 2*2 contingency table, should be provided.

The paper deserves consideration for publication with the suggested additions and changes.

Reviewer #2

(Remarks to the Author)
This manuscript reports on a variation of RPA that overcomes many of the limitations of RPA, most importantly the requirement for very long primers whose performance is difficult to predict using simple rules. They introduce the novel concept of an annexing probe, where a DNA strand that is long enough for efficient recombinase-mediated pairing with the target DNA strand is used to first annex the territory. This allows the loading of the proximal primer to the region that was just opened up, followed by polymerase extension. I suppose the polymerase has strand displacement synthesis activity—the authors should mention this—and it will displace the annexing probe. Importantly, the annexing probe is protected from

extension by DNA polymerase by an end cap, so it cannot serve as a primer. The distal primer is subsequently loaded to complete the process via the synthesis of the other strand. They demonstrate the operation of the principle using a variety of clinically relevant nucleic acids, demonstrate compatibility with lateral flow assays (LFA), develop a qPCR-like real-time fluorescence readout for kinetic measurements, and show evidence for single-molecule detection sensitivity. They also showed that a modification included in the annexing probe can be used for downstream operations such as LFA immobilization. Overall, the work is interesting and important, and is a strong candidate for Nature Communications.

The principle for ANINA can be better explained in the main text, at the beginning of the Results section. It was not until I had a look at Figure 1 that I understood the concept and why it is referred to as annexing.

In Figure 2, there are multiple gels that show more than one band for the amplification products. Why?

For the lateral flow assay, except for the very last example, they used a step of organic extraction to remove proteins before running the LFA. If this is necessary for high sensitivity, I don't think this method will be as easy as antigen tests. In that case, there should be a tradeoff between sensitivity (requiring organic extraction) and ease of use (not requiring organic extraction). Please explain why organic extraction was used in some cases but not in others.

The operation seems highly sensitive to the nature of the modification on the annexing probe. The text is a little unclear about the importance of the modification—in most cases a fluorescent dye—but which dye is used matters for whether the reaction works with high yield or not. In this sense, their method may be difficult for others to adopt, as it would require a lot of trial and error before finding the right dye and the right length for the annexing probe. This is a concern when the authors are claiming the method as a powerful new approach to overcome existing limitations. They acknowledge that "These findings indicate probe label chemistry contributes directly to annexation efficiency," and this would render the technology's performance difficult to predict, while also causing strong temperature dependence, etc.

They also mention in line 120 that probe labeling contributes toward annexation efficiency. How would that work?

It is still unclear to me why the loading of the distal primer does not require its own annexing probe. Can the authors provide an explanation?

Also, I would like to see an explanation of how the annexing probe does its job. They wrote in line 115 about "reduced secondary structure propensity." I can guess what they mean, but not with confidence.

Regarding line 203: "In contrast, in the absence of an AP and using a commercial RPA formulation..." I think this comparison to examine the role of the AP is problematic because they change two things: they change the RPA formulation in addition to the removal of the AP. They need to change only one thing.

"Additionally, only qANINA was able to detect the single copy EBV standard (all replicates) at ~9.3 minutes, demonstrating single copy analytical sensitivity." This is impressive. I wonder if they considered performing digital ANINA to truly demonstrate single-copy sensitivity.

"From the slope of the Dt relationship, the effective doubling time for EBV qANINA was ~14.5 seconds." This number should be a function of amplicon size. Please indicate what the size was.

I am wondering why they are able to see well-defined amplicons when the method would allow reinitiation of replication before the previous round is completed. When they quench the reaction, I would imagine there will be a lot of intermediate products that are not complete, but they don't seem to see them. Please explain.

Reviewer #3

(Remarks to the Author)
I co-reviewed this manuscript with one of the reviewers who provided the listed reports. This is part of the Nature Communications initiative to facilitate training in peer review and to provide appropriate recognition for Early Career Researchers who co-review manuscripts.

Version 1:

Reviewer comments:

Reviewer #2

(Remarks to the Author)
I am satisfied with the responses to my questions. I recommend publication.

Reviewer #3

(Remarks to the Author)
I co-reviewed this manuscript with one of the reviewers who provided the listed reports. This is part of the Nature Communications initiative to facilitate training in peer review and to provide appropriate recognition for Early Career

Researchers who co-review manuscripts.

Open Access This Peer Review File is licensed under a Creative Commons Attribution 4.0 International License, which permits use, sharing, adaptation, distribution and reproduction in any medium or format, as long as you give appropriate credit to the original author(s) and the source, provide a link to the Creative Commons license, and indicate if changes were made.
In cases where reviewers are anonymous, credit should be given to 'Anonymous Referee' and the source.
The images or other third party material in this Peer Review File are included in the article's Creative Commons license, unless indicated otherwise in a credit line to the material. If material is not included in the article's Creative Commons license and your intended use is not permitted by statutory regulation or exceeds the permitted use, you will need to obtain permission directly from the copyright holder.
To view a copy of this license, visit https://creativecommons.org/licenses/by/4.0/

Point-by-point response to the reviewers of manuscript (NCOMMS- 26-017541-T)

We thank all the Reviewers for their careful reading and constructive feedback on our manuscript titled "Annexing Isothermal Nucleotide Amplification (ANINA) Enables Probe-guided Amplification and Direct Detection at Ambient Temperature for a Point-of-Care Molecular Platform". We are pleased that Nature Communications finds our research interesting and important. We have carefully revised this manuscript to address all reviewer comments (marked in blue text) with point-by-point responses, see below. Revisions to the manuscript and supplementary files in response to reviewer comments are identified in red headings and *"italicized text"*.

We thank Reviewer 1 for their positive assessment of ANINA's potential for point-of-care diagnostics and address their specific comments below as well as revised the manuscript based on their recommendations.

Reviewer 1:

1. The ANINA method implemented during this study provides data to believe the promising potential for use of the technique for early detection of widespread infections and the possibility of point-of-care utility. This will help the industry.
2. The data proves the concept that in instrument-free mode, the field level detection will be possible while the samples are treated at room temperature.
3. The methodology and results are well explained.
4. Since such methodologies are closely linked to widespread use in the trade, therefore, it needs to be proven on clinical samples in field conditions and generate robust data. This will add value to the work and will be of direct use. Such clinical samples can be corroborated with real-time PCR and provide a good comparative view on the sensitivity of the test, meaning how many viral copies the method is able to detect. Sensitivity will be the most important factor for the industry to believe in the application of the results, which otherwise show a great promise.
5. Based on the actual field trials, the possible limitations of the test can also be defined.

Response: ANINA was validated in a natural host infection model using WSSV-infected shrimp processed through a fully instrument-free sample-to-answer workflow under field-mimicking conditions. The assay detected viral loads as low as 20 copies/µL, in agreement with results from qPCR across all samples and outperformed against a WOAH-certified commercial antigen test. We have revised the Discussion **(pages 24-25 and 26)** to more explicitly frame this model as translational validation under biologically and operationally relevant deployment conditions, clarify the importance of WSSV as a stringent field-relevant test case, and acknowledge that larger-scale validation across independent cohorts and field settings will be required to better understand and resolve potential limitations prior to actual deployment. Validation studies involving human clinical samples are ongoing and will be reported in future work **(revised Discussion Page 26).**

Revised Discussion on Page 24-25 (Lines 395-420):

"ANINA's integration into a fully equipment-free, 45-minute sample-to-answer workflow for point-of-care detection of WSSV infection in shrimp further demonstrates its translational feasibility in a biologically and operationally relevant diagnostic setting. WSSV, the causative agent of White Spot Disease, is a highly virulent pathogen recognized by WOAH as a major threat to global aquaculture. Infection spreads rapidly within shrimp ponds and can result in near-complete mortality within 3 to 7 days, creating a narrow window for detection and intervention[49]. Although laboratory-based qPCR assays can detect WSSV as early as 24 hours post infection (HPI), their deployment is constrained by centralized infrastructure and sample transport, delaying actionable results in field settings[51]. In contrast, existing rapid antigen tests typically lack sensitivity during early infection stages (<48 HPI), limiting timely outbreak-control measures.

In shrimp infected under natural exposure conditions, the integrated sample-to-answer workflow using ANINA reliably detected early stages (24–48 hours) of WSSV infection with sensitivity comparable to reference qPCR and improved performance relative to a commercial antigen test. This validation using naturally

infected host samples under biologically realistic conditions, including crude tissue-derived nucleic acids, heterogeneous infection dynamics, lyophilized reagents, and ambient incubation, highlights practical deployability and alignment with the ASSURED framework for point-of-care diagnostics[1-4]*. Diagnostic performance analysis using qPCR as the reference standard showed concordance between the integrated ANINA workflow and laboratory-based detection (Supplementary Table 2), although the limited cohort size currently supports only descriptive performance evaluation. Future work will involve experimental exclusivity testing against related shrimp pathogens, reproducibility and larger-scale validation studies across independent field cohorts and broader environmental conditions."*

Revised Discussion on Page 26: (Lines 468-469):

"*Validation using clinical samples involving human pathogens is ongoing."*

Reviewer 1:

6. The manuscript should include a brief discussion on the risk of non-specific amplification, which is inherent to isothermal amplification. Furthermore, carryover contamination risks associated with recombinase-based amplification should be addressed.

Response: We agree that non-specific amplification and carryover contamination are important considerations for isothermal amplification systems. We have revised the Discussion (**page 26-27**) to state that in ANINA, amplification depends on AP-guided spatial recruitment of the probe-adjacent primer, which introduces an additional layer of target-dependent spatial organization beyond conventional primer-only recombinase amplification. Across the conditions tested in this study, no detectable amplification products were observed in no-template controls, supporting low background amplification under the evaluated assay conditions. Potential primer–primer interactions were further minimized through in-silico screening for self-dimer and heterodimer formation during assay design.

We have revised the Discussion (**page 26-27**) to also address carryover contamination risks. As with other high-sensitivity nucleic acid amplification systems, ANINA remains susceptible to carryover contamination from amplified products. To mitigate this risk, reactions were performed using lyophilized closed-tube reaction formats with physical separation of pre- and post-amplification workflows. We now explicitly discuss these considerations and current contamination-control practices in the revised Discussion.

> Revised Discussion on Page 26-27 (Lines 470-477):
>
> "*As with other high-sensitivity isothermal amplification systems, ANINA may remain susceptible to nonspecific amplification and carryover contamination under inappropriate assay or workflow conditions. In the present study, no detectable amplification was observed in no-template controls across the evaluated conditions, consistent with AP-guided spatial regulation contributing to low background amplification. Standard contamination-control practices, including closed-tube lyophilized reactions and physical separation of pre- and post-amplification workflows, remain important for minimizing carryover contamination risk.*"

Reviewer 1:

7. A critical comparison of ANINA with nfo-RPA, SHERLOCK, and related technologies should be included. This comparison should include potential limitations of the method, including probe design constraints, multiplexing capability, and operational challenges.
9. To improve clarity and impact, it is recommended to include a comparative table summarizing key diagnostic parameters (LOD, assay time, equipment required, estimated cost per test) along with advantages and limitations in comparison with established methods.

Response: We agree with the reviewer's recommendations and have added a comparative table (**Table 2 on Page 29 in the revised manuscript**) summarizing ANINA alongside representative recombinase-based and CRISPR-assisted isothermal

methods, including RPA, RPA-nfo, SHERLOCK-RPA, and SIBA. The comparison includes assay architecture, primer and probe requirements, operating temperature, reaction time, direct LFA compatibility, auxiliary enzymatic requirements, detection mechanism, detection modalities, and reported analytical sensitivity. This table contextualizes the key differentiators of ANINA, including ambient-temperature operation, short-primer compatibility, direct lateral flow detection without probe cleavage, and a simplified three-oligonucleotide architecture within a lyophilized reaction format. We have also expanded the Discussion to acknowledge current limitations, including the need for target-specific oligonucleotide design, broader validation of higher-order multiplexing, and further mechanistic characterization of annexation. A comprehensive cost-per-test estimate is beyond the scope of this platform-focused study and would depend on manufacturing scale, reagent sourcing, and deployment format. We note that the lyophilized reaction architecture requires no cold chain for storage or distribution, representing a potential operational and logistical cost advantage over methods requiring cold-chain-dependent reagents or auxiliary enzymatic processing.

Revised Discussion on Page 26 (Lines 462-469):

"*Current limitations include the need for target-specific oligonucleotide design, broader validation of higher-order multiplexed amplification, and further mechanistic characterization of annexation. Although this study demonstrates feasibility of multiplexed endpoint detection and sensitivity to terminal primer mismatches, future work will evaluate higher-order multiplexing, tolerance to internal sequence variation, and performance across diverse pathogen strains and field settings. Broader inter-laboratory validation will be required to support large-scale deployment. Validation using clinical samples involving human pathogens is ongoing*."

New Table 2 on Page 29:

Table 2: Comparison of ANINA with representative recombinase-based isothermal amplification systems

Comparison Parameter	ANINA	RPA[13,20]	RPA-nfo[13,20]	RPA+ SHERLOCK[27]	SIBA[22,28-30]
**Assay architecture**	2 primers + 1 Annexing Probe (AP)	2 primers	2 primers + nfo probe	2 primers + target-specific gRNA + reporter probe	2 primers + Invasion oligonucleotide
**Operating temperature[a]**	20–45 °C	Typically 35–45 °C	Typically 35–45 °C	Typically 35–45 °C	Typically 40–45 °C
**Reaction time[a,b]**	5–30 min	5–30 min	10–30 min	~45–60 min	~30–60 min
**Primer Length**	Supports 15–35 nt primers	Typically 30–35 nt primers	Same as RPA	Same as RPA	Supports 16–23 nt primers
**Direct endpoint LFA detection**	Yes	No	Yes	Yes	No
**LFA Detection Probe design requirements**	25–40 nt AP with 5′ detection label and 3′ block	N/A	≥45 nt Nfo probe with internal abasic site, 5' detection label, and 3′ block	6–10 nt Reporter probe (5' detection label and 3' biotin label, non-target specific)	N/A
**Additional oligonucleotide design requirements**	None	None	None	Target-specific gRNA (~60–70 nt total; ~28 nt target-complementary region)	Invasion oligonucleotide (~>55 nt total; 5' non-homologous seeding region, 3' 2-O-methyl RNA region, 3' block)
**Auxiliary enzymes required for LFA detection**	None	N/A	Endonuclease IV (nfo)	Cas13a + T7 transcription	N/A
**LFA Detection mechanism**	Direct AP-associated detection	N/A	Probe cleavage-dependent	Collateral reporter cleavage	N/A
**Probe contributes to reaction specificity**	Yes	N/A	No	No	N/A
**Analytical sensitivity demonstrated**	Attomolar to low-copy detection	Attomolar to low-copy detection reported	Attomolar to low-copy detection reported	Attomolar to low-copy detection reported	Low-copy detection reported
**Equipment Required**	Doesn't require incubator	Typically requires incubator	Typically requires incubator	Typically requires incubator	Yes
**Compatible Detection modalities**	LFA, fluorescence, imaging	Fluorescence, imaging	LFA, fluorescence, imaging	LFA, fluorescence, imaging	Fluorescence, imaging

FootNotes:

[a] Operating conditions and time ranges represent typical assay conditions reported in the literature and/or demonstrated in this study.

[b] Amplification time may vary with reaction temperature and assay conditions.

Reviewer 1:

8. The Specificity of the assay should be further evaluated against closely related pathogens (eg, for WSSV, closely related shrimp viruses) to eliminate cross-reactivity.

Response: To further assess potential assay cross-reactivity, we performed in silico specificity analysis of the WSSV ANINA oligonucleotide set against representative

pathogens associated with common infectious diseases in shrimp, including AHPND, DIV1/SHIV, EHP, IHHNV, IMNV, NHP, TSV, and YHV. The highest-scoring alignments for each oligonucleotide–accession comparison were analyzed and are summarized in **Supplementary Table S3**. Across all non-WSSV pathogens and shrimp, only weak partial alignments with limited query coverage and poor overall alignment statistics were identified for a subset of primers, while no meaningful similarity was detected for the Annexing Probe. Importantly, no non-target organism demonstrated coordinated complementarity across the full oligonucleotide set required for productive ANINA amplification. These analyses support predicted analytical specificity of the WSSV ANINA assay. Experimental exclusivity testing against related shrimp pathogens will be included in future validation studies. The corresponding analysis and description have been added to the revised manuscript (**Result on Page 22, Discussion on Page 25, Methods 1 on Page 40**).

Revised Results on Page 22 (Lines 345-351):

"*Additionally, in-silico cross-reactivity analysis of the WSSV ANINA oligonucleotide set against non-WSSV representative pathogens of common shrimp infectious diseases showed no coordinated complementarity across the complete set required for productive amplification (Supplementary Table S3). Only weak partial alignments with low query coverage and poor overall complementarity were observed for a subset of primers, whereas no similarity was identified for the AP, thereby supporting predicted assay specificity for WSSV.*"

Revised Discussion on Page 25 (Lines 417-420):

"Future work will involve experimental exclusivity testing against related shrimp pathogens, reproducibility and larger-scale validation studies across independent field cohorts and broader environmental conditions."

Revised Methods section 1:

"*To evaluate potential cross-reactivity of the WSSV ANINA oligonucleotide set used in the integrated sample-to-answer assay, NCBI nucleotide BLAST analysis*

was performed against the intended target pathogen (WSSV), target host (shrimp, Litopenaeus vannamei) and representative causative pathogens of common shrimp infectious diseases, including AHPND (Vibrio spp.), DIV1/SHIV, EHP, IHHNV, IMNV, NHP, TSV, and YHV (Supplementary Table 3). BLAST parameters were set to search standard databases and the program was selected to optimize for highly similar sequences (megablast). For each primer–accession comparison, the highest-ranking alignment was selected based on the lowest E-value and highest Max Score. Max Score, Total Score, E-value, Percent Identity, and Query Coverage from this representative alignment were recorded for downstream analysis. When multiple accessions were evaluated for a given organism, the strongest off-target alignment identified among all accessions was retained to represent the maximum potential cross-reactivity for that organism."

New Supplementary Table 3:

Supplementary Table 3: In-silico cross-reactivity analysis of the WSSV ANINA oligonucleotide set against WSSV, host (shrimp) and non-WSSV representative pathogens of common shrimp infectious diseases

White Spot Syndrome Virus (WSSV; taxid: 342409)

NCBI Accession: NC_003225.3

WSSV ANINA Oligonucleotide	Max Score	Total Score	E-Value	Percent Identity	Query Cover
VP19 PP2-15nt	30.2	1506	3.50E-02	100%	100%
VP19 DP1-20nt	40.1	1023	1.00E-04	100%	100%
VP19 DP2-24nt	48.1	821	7.00E-07	100%	100%
VP19 AP2-38nt	71.3	71.3	5.00E-14	100%	100%

Pacific White Shrimp (*Litopenaeus vannamei*; taxid:6689)

NCBI Accession: GCF_042767895.1

WSSV ANINA Oligonucleotide	Max Score	Total Score	E-Value	Percent Identity	Query Cover
VP19 PP2-15nt	28.2	28.2	0.97	100%	93%
VP19 DP1-20nt	30.2	50.5	0.62	100%	75%
VP19 DP2-24nt	30.2	30.2	1.1	100%	63%
VP19 AP2-38nt	*No similarity found*	*No similarity found*	*No similarity found*	*No similarity found*	*No similarity found*

Acute Hepatopancreatic Necrosis Disease (AHPND; *Vibrio parahaemolyticus*; taxid: 670)

NCBI Accession: NZ_AP014859.1

WSSV ANINA Oligonucleotide	Max Score	Total Score	E-Value	Percent Identity	Query Cover
VP19 PP2-15nt	28.2	489	8	100%	100%
VP19 DP1-20nt	30.2	285	4.1	100%	100%
VP19 DP2-24nt	28.2	103	28	100%	100%
VP19 AP2-38nt	*No similarity found*	*No similarity found*	*No similarity found*	*No similarity found*	*No similarity found*

Taura Syndrome Virus (TSV; taxid: 3048357)

NCBI Accession: NC_003005.1

WSSV ANINA Oligonucleotide	Max Score	Total Score	E-Value	Percent Identity	Query Cover
VP19 PP2-15nt	16.4	16.4	17	100%	53%
VP19 DP1-20nt	16.4	145	31	100%	100%
VP19 DP2-24nt	18.3	94.1	11	100%	83%
VP19 AP2-38nt	*No similarity found*	*No similarity found*	*No similarity found*	*No similarity found*	*No similarity found*

Decapod Iridescent Virus / Shrimp Hemocyte Iridescent Virus (DIV1/SHIV; taxid: 3428192)

NCBI Accession: NC_055165.1

WSSV ANINA Oligonucleotide	Max Score	Total Score	E-Value	Percent Identity	Query Cover
VP19 PP2-15nt	24.3	6344	0.12	100%	100%
VP19 DP1-20nt	16.4	118	37	100%	92%
VP19 DP2-24nt	18.3	153	14	100%	96%
VP19 AP2-38nt	*No similarity found*	*No similarity found*	*No similarity found*	*No similarity found*	*No similarity found*

Yellowhead Disease Virus (YHV; taxid: 3048455)

NCBI Accession: NC_043505.1

WSSV ANINA Oligonucleotide	Max Score	Total Score	E-Value	Percent Identity	Query Cover
VP19 PP2-15nt	18.3	440	3	100%	100%
VP19 DP1-20nt	22.3	563	0.38	100%	100%
VP19 DP2-24nt	20.3	596	2	100%	100%
VP19 AP2-38nt	*No similarity found*	*No similarity found*	*No similarity found*	*No similarity found*	*No similarity found*

Infectious Hypodermal and Hematopoietic Necrosis Virus (IHHNV; taxid: 3052615)

NCBI Accession: NC_002190.2

WSSV ANINA Oligonucleotide	Max Score	Total Score	E-Value	Percent Identity	Query Cover
VP19 PP2-15nt	18.3	47.1	5.2	100%	100%
VP19 DP1-20nt	16.4	118	37	91.67%	100%
VP19 DP2-24nt	18.3	153	14	100%	96%
VP19 AP2-38nt	*No similarity found*	*No similarity found*	*No similarity found*	*No similarity found*	*No similarity found*

Necrotising Hepatopancreatitis (NHP; *Hepatobacter penaei*, taxid: 1274402)

NCBI Accession: NZ_JQAJ00000000

WSSV ANINA Oligonucleotide	Max Score	Total Score	E-Value	Percent Identity	Query Cover
VP19 PP2-15nt	18.3	32.7	0.62	100%	60%
VP19 DP1-20nt	18.3	106	0.97	100%	90%
VP19 DP2-24nt	18.3	47.1	1.3	100%	46%
VP19 AP2-38nt	*No similarity found*	*No similarity found*	*No similarity found*	*No similarity found*	*No similarity found*

Penaeid Shrimp Infectious Myonecrosis Virus (IMNV; taxid: 3426466)

NCBI Accession: NC_007915.3

WSSV ANINA Oligonucleotide	Max Score	Total Score	E-Value	Percent Identity	Query Cover
VP19 PP2-15nt	20.3	192	0.54	100%	100%
VP19 DP1-20nt	18.3	313	4.2	100%	95%
VP19 DP2-24nt	18.3	198	5.9	100%	92%
VP19 AP2-38nt	*No similarity found*	*No similarity found*	*No similarity found*	*No similarity found*	*No similarity found*

***Enterocytozoon hepatopenaei* (EHP; taxid: 646526)**

NCBI Accession:

WSSV ANINA Oligonucleotide	Max Score	Total Score	E-Value	Percent Identity	Query Cover
VP19 PP2-15nt	20.3	51	0.55	100%	67%
VP19 DP1-20nt	22.3	96.1	0.27	100%	70%
VP19 DP2-24nt	20.3	65.4	1.5	100%	83%
VP19 AP2-38nt	*No similarity found*	*No similarity found*	*No similarity found*	*No similarity found*	*No similarity found*

Reviewer 1:

10. Statistical analysis, like Probit regression for determining LoD and Diagnostic performance evaluation (true positive, true negative, false positive, and false negative), diagnostic sensitivity, and specificity using a 2*2 contingency table, should be provided.

Response: Diagnostic performance metrics including true positive, true negative, false positive, and false negative calls, diagnostic sensitivity, and specificity have been calculated and reported in a 2x2 contingency table for the shrimp WSSV-infection studies using qPCR as the Reference Standard (**revised Supplementary Table S2**). Given the limited sample size of the natural host infection study, results are interpreted descriptively and this is explicitly noted in the Discussion (**Page 25**). Probit-based LOD determination will be performed in future larger-scale validation studies.

> Revised Discussion on Page 25 (Lines 414-420):
>
> "*Diagnostic performance analysis using qPCR as the reference standard showed concordance between the integrated ANINA workflow and laboratory-based detection (Supplementary Table 2), although the limited cohort size currently supports only descriptive performance evaluation. Future work will involve experimental exclusivity testing against related shrimp pathogens, reproducibility and larger-scale validation studies across independent field cohorts and broader environmental conditions*."
>
> Revised Supplementary Table 2:

Supplementary Table 2: qPCR assay results and Diagnostic performance evaluation of the WSSV-shrimp infection studies (Fig. 7 and Extended Data Fig. 7)

Time-points	Shrimp #	Tissue Type	Extraction replicate #	Ct	WSSV copies/uL	qPCR call (each replicate)	ANINA LFA call (each replicate)	Average copy numbers	qPCR call (each shrimp)[1]	ANINA call (each shrimp)[1]	RAT call (each shrimp)[1]
24 hours	1	Muscle	1	38.25	2.07E+02	Positive	Positive	4.41E+02	Positive	Positive	Invalid
			2	36.38	6.76E+02	Positive	Positive				
		Pleopod	1	No Ct	0.00E+00	Negative	Positive				
			2	No Ct	0.00E+00	Negative	Negative				
	2	Muscle	1	31.63	6.81E+03	Positive	Positive	3.36E+04	Positive	Positive	Positive
			2	30.06	1.87E+04	Positive	Positive				
		Pleopod	1	28.40	5.11E+04	Positive	Positive				
			2	28.26	5.79E+04	Positive	Positive				
	3	Muscle	1	36.62	3.11E+02	Positive	Positive	6.18E+03	Positive	Positive	Negative
			2	33.31	2.32E+03	Positive	Positive				
		Pleopod	1	30.69	1.34E+04	Positive	Positive				
			2	31.20	8.73E+03	Positive	Positive				
48 hours	1	Muscle	1	36.37	1.93E+01	Positive	Positive	4.95E+01	Positive	Positive	Negative
			2	No Ct	0.00E+00	Negative	Positive				
		Pleopod	1	35.92	7.97E+01	Positive	Positive				
			2	No Ct	0.00E+00	Negative	Negative				
	2	Muscle	1	21.82	3.44E+05	Positive	Positive	4.61E+06	Positive	Positive	Positive
			2	20.65	7.60E+05	Positive	Positive				
		Pleopod	1	17.29	7.30E+06	Positive	Positive				
			2	16.80	1.00E+07	Positive	Positive				
	3	Muscle	1	31.87	3.97E+02	Positive	Positive	2.75E+02	Positive	Positive	Negative
			2	No Ct	0.00E+00	Negative	Positive				
		Pleopod	1	32.33	3.00E+02	Positive	Positive				
			2	35.88	1.27E+02	Positive	Positive				
Non-infected	1	Muscle	1	No Ct	0.00E+00	Negative	Negative	0.00E+00	Negative	Negative	Negative
		Pleopod	1	No Ct	0.00E+00	Negative	Negative				
qPCR Template controls	PTC	PTC		17.22	8.03E+06	Positive	Positive	8.03E+06	Positive	Positive	N/A
	NTC	NTC		No Ct	0.00E+00	Negative	Negative	0.00E+00	Negative	Negative	N/A

[1] If ≥50% replicates (2/4 or 3/4 or 1/2) are positive on call, then shrimp is positive for that test. If <50% replicates is positive (1/4) then it is negative

Diagnostic Sensitivity (Dse) = #(True Positives)/(#(True Positives) + #(False Negatives))

Diagnostic Specificity (Dsp) = #(True Negatives)/(#(True Negatives) + #(False Positives))

ANINA WSSV Integrated Assay		qPCR (Lab reference assay) Positive	Negative	Total
	Positive	6	0	6
	Negative	0	1	1
	Total	6	1	7
	Relative Dx Sensitivity (DSe)			100.0%
	Relative Dx Specificity (DSp)			100.0%

Innocreate WSSV RAT		qPCR (Lab reference assay) Positive	Negative	Total
	Positive	2	0	2
	Negative	3	1	4
	Total	5	1	6
	Relative Dx Sensitivity (DSe)			40.0%
	Relative Dx Specificity (DSp)			100.0%

We acknowledge the accurate and detailed description of the ANINA mechanism from Reviewer 2 and their positive assessment of the work. We address each specific comment below.

Reviewer 2

Paragraph 1: This manuscript reports on a variation of RPA that overcomes many of the limitations of RPA, most importantly the requirement for very long primers whose performance is difficult to predict using simple rules. They introduce the novel concept of an annexing probe, where a DNA strand that is long enough for efficient recombinase-mediated pairing with the target DNA strand is used to first annex the territory. This allows the loading of the proximal primer to the region that was just opened up, followed by polymerase extension. I suppose the polymerase has strand displacement synthesis activity—the authors should mention this—and it will displace the annexing probe. Importantly, the annexing probe is protected from extension by DNA polymerase by an end cap, so it cannot serve as a primer. The distal primer is subsequently loaded to complete the process via the synthesis of the other strand. They demonstrate the operation of the principle using a variety of clinically relevant nucleic acids, demonstrate compatibility with lateral flow assays (LFA), develop a qPCR-like real-time fluorescence readout for kinetic measurements, and show evidence for single-molecule detection sensitivity. They also showed that a modification included in the annexing probe can be used for downstream operations such as LFA immobilization. Overall, the work is interesting and important, and is a strong candidate for Nature Communications.

Response: The reviewer is correct. We have revised the introduction (**Page 4**) to clarify that ANINA uses a strand-displacing DNA polymerase, and that primer extension results in displacement of the AP during amplification.

> Revised Introduction on Page 4 (Lines 75-77):
>
> "*The strand-displacing polymerase Bsu then extends the PP and displaces the non-extendible AP, allowing subsequent rounds of annexation.* "

Reviewer 2

Paragraph 2: The principle for ANINA can be better explained in the main text, at the beginning of the Results section. It was not until I had a look at Figure 1 that I understood the concept and why it is referred to as annexing.

Response: We agree that a clearer mechanistic description at the opening of the Results section improves comprehension. We have expanded the mechanism of ANINA in the main text in the Introduction (**on Page 4**) before Results.

> Revised Introduction on Page 4 (Lines 64-84):
>
> "*Central to ANINA is a three-oligonucleotide architecture (Fig. 1b) consisting of an Annexing Probe (AP) with a 3' polymerase block, a probe-adjacent primer (PP), and a downstream primer (DP). The PP and DP serve analogous functions to forward and reverse primers in other amplification systems, but their directionality is based on AP–primer spatial geometry. The AP, rather than being a passive reporter, actively participates in amplification through spatial annexation of the PP. The AP, designed to be long enough for efficient recombinase loading, interacts with recombinase (UvsX) and single-strand binding protein (GP32) to form a nucleoprotein complex that invades double-stranded DNA and binds its complementary sequence to generate a locally opened D-loop structure. We propose that this AP-mediated opening enables the adjacent PP to productively anneal, even when the PP itself is too short for efficient recombinase loading. The strand-displacing polymerase Bsu then extends the PP and displaces the non-extendible AP, allowing subsequent rounds of annexation. PP extension also exposes the complementary strand for efficient DP annealing and amplification progression. This spatial organization enables specific and efficient amplification under primer-length and temperature conditions poorly supported by conventional recombinase-based systems. Notably, ANINA supports efficient and specific amplification at 25°C with probe-adjacent primers (PPs) as short as 10 nt and downstream primers (DPs) as short as 15 nt, lengths that are substantially below those typically supported in recombinase-based systems.*"

Reviewer 2

Paragraph 3: In Figure 2, there are multiple gels that show more than one band for the amplification products. Why?

The additional bands observed in Fig. 2 occur consistently with target-containing reactions involving DP3 (e.g., Fig. 2b lanes 1P and 4P; Fig. 2c lanes 1, 4, and 7; Fig. 2d lanes 1 and 4; Fig. 2f lanes 1, 4, and 7; Fig. 2g lanes 3 and 6), and are absent in no-template controls (Fig. 3b,d). Thus, these are reproducible, and target-specific products rather than nonspecific background amplification and may likely arise from differences in conformation of the amplicons generated during amplification or due to altered PAGE mobility. Importantly, the primary interpretation of Fig. 2 relies on the presence or absence of amplification and the relative amplification efficiency across AP–PP spatial configurations, which remain clear despite these secondary bands. We now explicitly acknowledge this in **Figure 2 legend**.

> Revised Figure 2 legend on Page 6:
>
> *"Some reactions produced reproducible secondary amplicon bands, particularly in reactions involving DP3; these bands were target-dependent and absent in no-template controls."*

Reviewer 2

Paragraph 4: For the lateral flow assay, except for the very last example, they used a step of organic extraction to remove proteins before running the LFA. If this is necessary for high sensitivity, I don't think this method will be as easy as antigen tests. In that case, there should be a tradeoff between sensitivity (requiring organic extraction) and ease of use (not requiring organic extraction). Please explain why organic extraction was used in some cases but not in others.

Response: The reviewer raises an important practical point. Organic extraction was used in several early feasibility and mechanistic experiments for two reasons: (1) to test

whether AP-associated lateral flow detection persists after protein removal, and (2) to standardize initial lateral flow evaluation using the manufacturer-recommended workflow and detection buffer. Importantly, organic extraction or purification is not required for ANINA-based lateral flow detection, as demonstrated by the fully integrated equipment-free workflow in Fig. 7, where amplification products were applied directly to the lateral flow strips without purification.

During workflow integration, we observed that direct application of unpurified reactions using the manufacturer-provided detection buffer increased background signal in template-negative samples. This background was substantially reduced by replacing the kit buffer with an optimized PBS-based buffer while preserving positive signal intensity. These findings suggest that buffer composition, rather than protein removal, is the primary determinant of background signal, and that the earlier extraction step is not required for the ANINA detection mechanism itself. We now clarify this distinction in the revised Discussion (**on Page 25**) and Methods section 7a (**on Page 45**).

> Revised Discussion on Page 25 (Lines 430-435):
>
> *"Lateral flow signal was observed only in target-containing reactions and persisted after enzyme removal, arguing against nonspecific aggregation or residual protein-mediated capture. Successful LFA detection was also achieved by direct application of unpurified WSSV amplification products to lateral flow strips using an optimized PBS-based detection buffer, demonstrating that high-sensitivity detection can be achieved without organic extraction (Fig. 7)."*
>
> Revised Methods 7a section:
>
> *"For the instrument-free workflow, amplification reactions were diluted directly without organic extraction or purification into an optimized PBS-based detection buffer without cleanup. The PBS buffer reduced background signal in template-negative reactions while preserving positive signal intensity."*

Reviewer 2

Paragraph 5: The operation seems highly sensitive to the nature of the modification on the annexing probe. The text is a little unclear about the importance of the modification—in most cases a fluorescent dye—but which dye is used matters for whether the reaction works with high yield or not. In this sense, their method may be difficult for others to adopt, as it would require a lot of trial and error before finding the right dye and the right length for the annexing probe. This is a concern when the authors are claiming the method as a powerful new approach to overcome existing limitations. They acknowledge that "These findings indicate probe label chemistry contributes directly to annexation efficiency," and this would render the technology's performance difficult to predict, while also causing strong temperature dependence, etc.

Response: The reviewer raises a valid concern regarding assay adoptability and predictability. Across the conditions evaluated in this study, FAM-labeled APs consistently supported efficient amplification and lateral flow detection across multiple targets and temperature conditions. While our results indicate that 5′ AP label chemistry can influence annexation efficiency, likely through effects of aromatic structure and linker composition, we found fluorescein-based labels provide robust performance without requiring target-specific optimization. Because FAM is already widely used in commercial lateral flow systems, this also facilitates practical implementation and assay standardization. We now clarify these observations and explicitly discuss current AP design considerations and optimization constraints in the revised Discussion (**on Page 24**).

Revised Discussion on Page 24 (Lines 384-388):

"Notably, AP label chemistry itself contributes to productive annexation beyond serving as a detection handle, although 5' FAM-labeled APs consistently supported efficient amplification and lateral flow detection across targets and temperature conditions. These features position ANINA as a generalizable amplification framework rather than a target-specific assay."

Reviewer 2

Paragraph 6: They also mention in line 120 that probe labeling contributes toward annexation efficiency. How would that work?

Response: Our current hypothesis is that the 5′ AP label contributes to annexation by altering the local structural and physicochemical environment of the recombinase-AP–target complex. The label may influence recombinase loading, stabilize the invading AP strand, promote local duplex distortion near the AP terminus, or generate steric and electrostatic effects that facilitate productive PP recruitment. This interpretation is consistent with the observed dependence of annexation efficiency on AP labeling, label identity, temperature, AP length, and AP–PP spacing. We now clarify in the revised Discussion (**on Page 26**) that further structural and mechanistic elucidation of annexation and the role of probe label chemistry will be important directions for future work. Further mechanistic investigation is beyond the scope of the present study but will be addressed in future development studies.

> Revised Discussion on Page 26 (Lines 444-449):
>
> "*The contribution of AP label chemistry to annexation efficiency further suggests that productive annexation depends not only on sequence complementarity but also on the local structural and physicochemical properties of the AP–target complex. We hypothesize that 5′ labels may influence recombinase loading, duplex accessibility, or local steric and electrostatic interactions that facilitate productive PP recruitment.*"
>
> Revised Discussion on Page 26 (Lines 460-461):
>
> "*Further structural elucidation of annexation and the role of probe label chemistry will be investigated in future work.*"

Reviewer 2

Paragraph 7: It is still unclear to me why the loading of the distal primer does not require its own annexing probe. Can the authors provide an explanation?

Response: Annexation is required primarily during amplification initiation, particularly for short probe-adjacent primers (PPs) that are inefficient at independently mediating recombinase-assisted strand invasion. Strand-displacing extension from the PP generates an accessible single-stranded region on the opposite strand, allowing even short downstream primers (DPs) to anneal productively without requiring AP-guided recruitment. If the DP is itself recombinase-compatible in length, it may also participate in conventional recombinase-mediated strand invasion, which could further improve amplification kinetics and yield, consistent with our observations when using longer DPs. Thus, ANINA appears to involve a division of roles between initiation and propagation: the AP is important for productive initiation at the PP-binding site, whereas subsequent amplification cycles generate accessible amplicon templates that permit DP annealing and amplification progression without requiring a second annexing probe. We have clarified this distinction in the revised Discussion (**on Page 23**).

> Revised Discussion on Page 23 (Lines 371-375):
>
> *"A single AP expands usable PP lengths from 10–35 nt and DP lengths from 15–35 nt while reducing dependence on primer thermodynamic parameters and retaining specificity at 25°C within 30-minute assay times. This likely occurs because PP extension generates a partially displaced amplicon intermediate that exposes the complementary strand for DP annealing without requiring AP-mediated recruitment."*

Reviewer 2

Paragraph 8: Also, I would like to see an explanation of how the annexing probe does its job. They wrote in line 115 about "reduced secondary structure propensity." I can guess what they mean, but not with confidence.

Response: We apologize for the lack of clarity and address two distinct aspects of this comment.

Firstly, to more clearly explain our working model for how annexation may occur, we have revised the Results and Discussion sections to distinguish the experimental observations from the mechanistic model they support. Based on our observations that probe–amplicon associations persist after enzyme removal and depend on AP length, AP–PP spacing, and 5′ label chemistry, we propose that annexation involves higher-order interactions beyond canonical Watson–Crick hybridization alone. In this model, the AP first undergoes recombinase-mediated pairing with the target duplex to generate a locally accessible AP-bound region adjacent to the PP-binding site. Because the AP is longer and recombinase-compatible, it can nucleate strand invasion under conditions where the short PP alone is insufficient. The adjacent PP can then anneal within this locally destabilized region and be extended by polymerase. We further hypothesize that AP label chemistry contributes to annexation by influencing the local structural and physicochemical properties of the AP–target complex, including steric, ionic, or protein-mediated interactions. This model is also consistent with the observation that annexation depends on AP–PP geometry because the accessible region generated by the AP is local and decays with distance from the AP boundary. We now clarify that the structural basis of annexation remains unresolved (**Discussion on Page 26**).

Secondly, with respect to “reduced secondary structure propensity,” this refers to reduced self-complementarity and hairpin-forming tendency of the primer itself. The shifted PP1(−2) variant has a substantially lower predicted secondary structure melting temperature than the original PP1-15nt primer (3.5°C versus 19.7 °C; Table 1), indicating reduced intramolecular folding of PP1(-2) at 25 °C. We therefore interpret the improved amplification efficiency of PP1(−2) as consistent with greater primer accessibility for annealing and extension. The manuscript has been revised to state this more directly (**Results 1 on Page 7**).

Revision Discussion on Page 26 (Lines 450-461):

"*Collectively, these findings support annexation as a spatially organized primer recruitment mechanism distinct from established recombinase-assisted strand invasion or probe-cleavage strategies. We propose that annexation begins when the recombinase-loaded AP invades the target duplex to generate a locally accessible AP-bound heteroduplex region adjacent to the PP-binding site. The AP-associated local environment may be further stabilized by label-dependent steric or physicochemical interactions that facilitate productive PP annealing and extension by the strand-displacing polymerase. This model explains why AP–PP proximity strongly influences amplification efficiency, why shortening the PP can preserve amplification when AP proximity is maintained, and why increasing AP–PP distance reduces efficiency even when primer length is unchanged. Further structural elucidation of annexation and the role of probe label chemistry will be investigated in future work."*

Revised Results on Page 7 (Lines 116-123):

"*To separate primer-length effects from spatial positioning, we evaluated shorter PP1 variants (15 nt and 12 nt) and a 3'-shifted variant, PP1(−2), which preserves primer length but alters AP–PP spacing. In the presence of AP, both 15 nt and 12 nt PPs supported efficient amplification with all DPs (Fig. 2c). The shifted PP1(−2) improved yield with the shortest DP, which is potentially because it has a substantially lower predicted secondary structure melting temperature than the PP1-15nt primer (3.5°C versus 19.7°C; Table 1), indicating reduced intramolecular folding at 25°C and greater primer accessibility for annealing and extension*."

Reviewer 2

Paragraph 9: Regarding line 203: "In contrast, in the absence of an AP and using a commercial RPA formulation..." I think this comparison to examine the role of the AP is problematic because they change two things: they change the RPA formulation in addition to the removal of the AP. They need to change only one thing.

Response: The reviewer correctly identifies a confound in the original comparison. The experiment in Supplementary Fig. 4b was not intended as a strict single-variable mechanistic comparison. The purpose was to demonstrate that failure of short primers to amplify efficiently under ambient conditions in the absence of an AP is not unique to the recombinase-based formulation used in this study and is also observed in a widely used commercial RPA system.

We have revised Supplementary Fig. 4b to include the full gel image, including commercial RPA reactions performed in the presence of the AP (lanes 13–14). These lanes show that AP-containing reactions are compatible with the commercial RPA formulation. We have revised the figure legend (**on Page 31**) and Results 3 text (**on Page 13**) accordingly.

Revised Results on Page 13 (Lines 208-212):

"*Using a commercial RPA formulation, we show that detectable amplification with the short DP was restricted to longer PPs (≥30 nt), highlighting the intrinsic length constraints of conventional recombinase-based amplification at ambient temperatures are not unique to the formulation used in this study (Supplementary Fig. 4b)*."

Revised Supplementary Fig 4b:

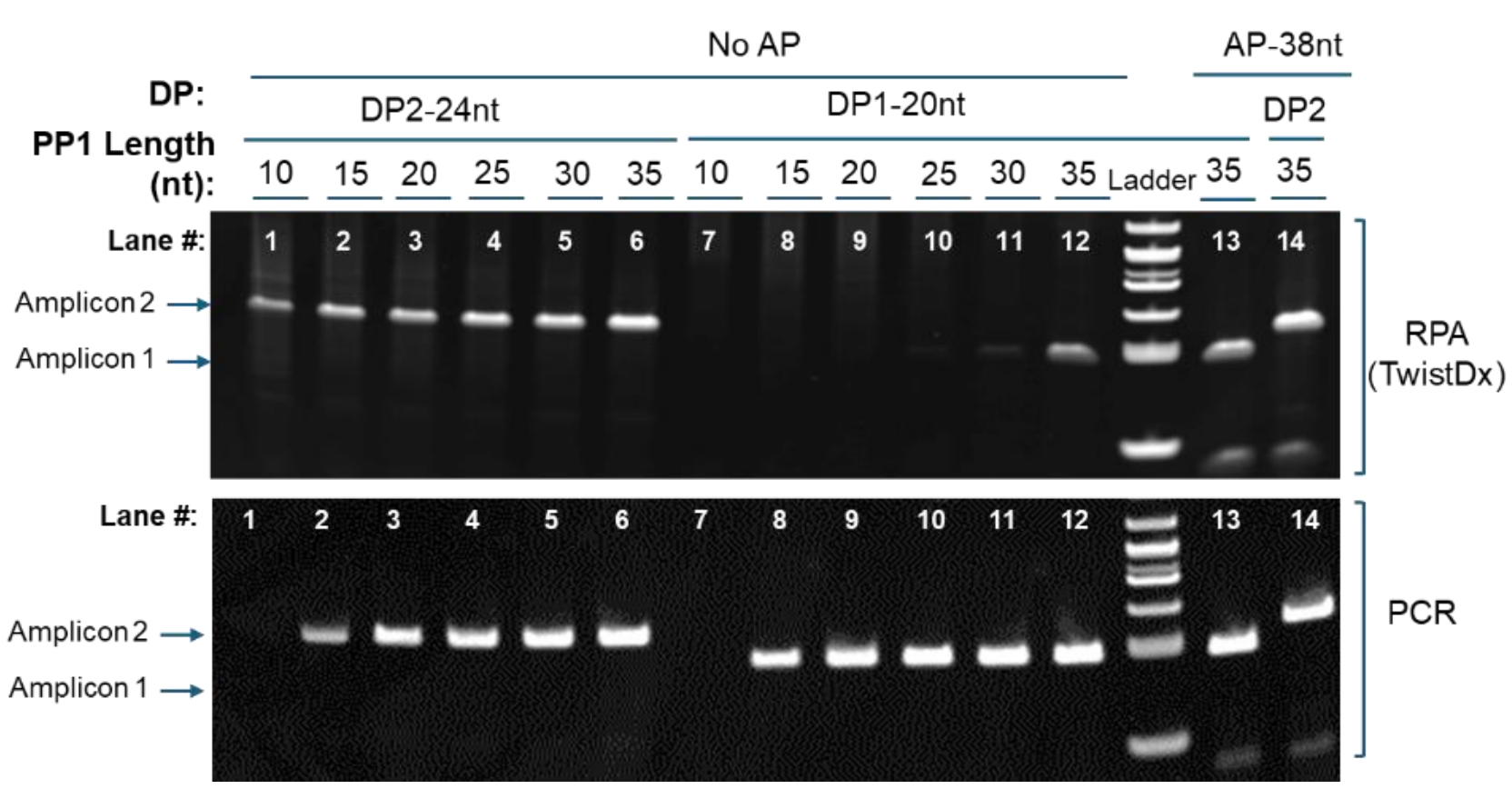

Revised legend for Supplementary Fig 4b:

“*PAGE results from RPA with TwistDx Basic reaction components (top panel) incubated at 25º C for 30 minutes, and PCR with 40 thermocycles (bottom panel). PP1 of various lengths shown in schematics in Fig. 2a and Fig. 4a were used as Forward Primers while DPs (DP1-20nt and DP2-24nt) were used as Reverse Primers. Lanes 1–12 were performed in the absence of AP, while lanes 13–14 contained AP1-38nt, demonstrating compatibility of AP-containing reactions with the commercial formulation. These lanes are not used to infer AP-dependent rescue of short-primer amplification. Corresponding ANINA reactions are shown in Fig. 2b and Fig. 4b.*”

Reviewer 2

Paragraph 10: "Additionally, only qANINA was able to detect the single copy EBV standard (all replicates) at ~9.3 minutes, demonstrating single copy analytical sensitivity." This is impressive. I wonder if they considered performing digital ANINA to truly demonstrate single-copy sensitivity.

Response: We agree that digital ANINA would provide a rigorous framework for absolute single-molecule quantification and represents a compelling future iteration of the platform. In the current study, our claim is limited to analytical detection of low-copy inputs under bulk reaction conditions, where all replicates containing the single-copy EBV standard generated detectable amplification signals. While this supports highly sensitive low-copy detection, digital partitioning approaches would enable more definitive characterization of single-molecule occupancy and stochastic amplification behavior. Digital ANINA is being explored as a next-generation platform and will be reported in future work.

Reviewer 2

Paragraph 11: "From the slope of the Dt relationship, the effective doubling time for EBV qANINA was ~14.5 seconds." This number should be a function of amplicon size. Please indicate what the size was.

Response: The effective doubling time reported for EBV qANINA was determined using the EBV ANINA oligonucleotides that generate a 161 bp amplicon. We agree that amplification kinetics, including effective doubling time, may vary with amplicon size and assay architecture; however, systematic evaluation of amplicon-length dependence was beyond the scope of the present study. We have added the amplicon size information to the relevant Results text (**on Page 18**) and Fig. 6 legend (**on Page 17**).

> Revised Result on Page 18 (Lines 275-277):
>
> *"Reactions using ANINA sets targeting EBV (EBV PP-12nt, DP2-29nt, and AP-40nt amplifying a 161 bp amplicon) were run isothermally at 39°C on a qPCR instrument without thermocycling, with fluorescence recorded every 10 seconds."*
>
> Revised legend for Fig. 6 on Page 17:
>
> *"qANINA reactions were performed by adding EvaGreen dye to EBV-specific ANINA reactions (161 bp target amplicon) and monitoring fluorescence isothermally at 39°C on an Agilent AriaMx instrument, with signal acquisition every 10 seconds for 10 minutes."*

Reviewer 2

Paragraph 12: I am wondering why they are able to see well-defined amplicons when the method would allow reinitiation of replication before the previous round is completed. When they quench the reaction, I would imagine there will be a lot of intermediate products that are not complete, but they don't seem to see them. Please explain.

Response: The reviewer raises an important mechanistic point. Because ANINA is an isothermal amplification process with multiple concurrent amplification events, transient partially extended intermediates are expected to occur during the reaction. However, full-length amplicons predominate in the PAGE analyses, likely because Bsu is a highly processive strand-displacing polymerase and most initiated extension events rapidly proceed to completion before subsequent strand invasion events occur. In addition,

PAGE preferentially visualizes the most abundant and discrete accumulated products at the reaction endpoint, whereas transient intermediate species are expected to be heterogeneous and lower abundance.

Consistent with the reviewer's expectation, we occasionally observed higher-molecular-weight products under conditions associated with faster amplification kinetics, including higher template concentrations (Fig. 3f lanes 8–9; Fig. 5d lanes 8–10) and elevated amplification temperatures (Supplementary Fig. 3a lanes 1.1–1.2; Supplementary Fig. 5e). These species may represent partially extended or branched amplification intermediates generated during overlapping amplification cycles. We have added a brief clarification regarding this point to the revised Discussion (**on Page 25-26**).

Revised Discussion on Page 25-26 (Lines 439-444):

"*Although PAGE analyses predominantly showed discrete full-length amplicons, occasional higher-molecular-weight species were observed under conditions associated with accelerated amplification kinetics, including elevated template concentrations and temperatures. These species may represent transient partially extended or branched amplification intermediates generated during overlapping isothermal amplification cycles.*"

Reviewer 3

I co-reviewed this manuscript with one of the reviewers who provided the listed reports. This is part of the Nature Communications initiative to facilitate training in peer review and to provide appropriate recognition for Early Career Researchers who co-review manuscripts.

Response: We appreciate the reviewer's careful co-evaluation of our manuscript.
